# Visualisation of J-type counter-current chromatography: A route to understand hydrodynamic phase distribution and retention

**DOI:** 10.1016/j.chroma.2012.03.039

**Published:** 2012-05-25

**Authors:** Yue Hugh Guan, Remco N.A.M. van den Heuvel, Ying-Ping Zhuang

**Affiliations:** a State Key Laboratory of Bioreactor Engineering, & The College of Biotechnology, East China University of Science & Technology, Shanghai 200237, China; b Brunel Institute for Bioengineering, Brunel University West London, Uxbridge, Middlesex UB8 3PH, UK

**Keywords:** ATPS, aqueous two-phase system, CCC, counter-current chromatography, H, head, HSCCC, high-speed counter-current chromatography, LP, lower phase, S, supplementary material, T, tail, UP, upper phase, Counter-current chromatography, Aqueous two-phase systems, Spiral column holders, Helical column, Stroboscopic imaging, Stationary phase retention

## Abstract

This paper has addressed decade sought-after questions on phase bilateral distribution and stationary phase retention in any J-type high-speed counter-current chromatographic (CCC) centrifuge. Using a 2-D spiral column operated on such a CCC device and an aqueous two-phase system, this work systematically observed the phase interaction during transitional period and at dynamic equilibration under stroboscopic illumination. The experimental results thus obtained were used to examine the effects of the liquid–solid friction force, tangential centrifugal force, and physical properties of the two-phase system on hydrodynamic phase behaviour. We identified that (a) density difference between lower and upper phases is the critical factor to cause unusual phase bilateral distribution in the 2-D spiral column and (b) interfacial tension (manifested primarily as phase settling time) of any two-phase system is the critical factor in explaining inability to retain stationary phase in 3-D helical column and, for certain flow modes, in the 2-D spiral column. This work thus has extended or modified the well-established rule-of-thumb for operating J-type CCC devices and our conclusions can accommodate virtually all the anomalies concerning both hydrophobic and hydrophilic phase systems. To this end, this work has not only documented valuable experimental evidences for directly observing phase behaviour in a CCC column, but also finally resolved fundamentally vital issues on bilateral phase distribution orientation and stationary phase retention in 2-D spiral and 3-D helical CCC columns. Revised recommendations to end users of this technology could thus be derived out of the essence of the present work presumably following further experimental validation and a consensus in the CCC R&D and manufacturing circle.

## Highlights

► Phase behaviour of an aqueous two-phase system was visualised in counter-current chromatography. ► High density difference causes phase bilateral distribution anomalies. ► Low interfacial tension or settling time makes columns not retain stationary phase. ► Mobile phase flow orientation rule is modified.

## 1 Introduction

Any form of counter-current chromatography (CCC) is featured by a liquid stationary phase, through which the mobile phase flows, and thus chromatographic separation of dissolved components is achieved through numerous partition steps between the two liquid phases along a CCC column. There is an established portfolio of seal-free flow-through CCC centrifuge schemes, in which J-type has been the most popular due primarily to its construction simplicity, operation robustness and scalability [1,2].

The invention of high-speed CCC (HSCCC) by Ito [3] was the most significant milestone for impacting on a steadily increasing range of applications and for driving commercialisation of CCC apparatus. HSCCC is characterised by both fast mobile phase flow and high stationary phase retention. By all means, the liquid “stationary” phase can in certain situations be made to flow either simultaneously or intermittently with the “mobile” phase in CCC. Compared to HSCCC, the scope of (gravity-driven) slow speed CCC has been limited to rather specific applications.

**Fig. 1:**
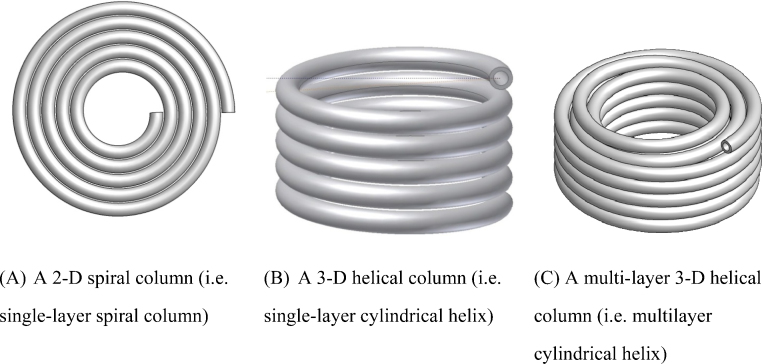
Illustration for the three column (coil) geometries discussed and compared in this work.

On a macro scale, a chromatographic process usually entails continuous and thus gradual concentration profile development for each component along the column longitudinal path [4]. Shortly ensuing the invention of HSCCC, Ito used a 2-D model [5] to show that the separation process under s dynamic J-type centrifuge scheme is governed by a discrete pattern of phase mixing and settling cycle over each rotation. Indeed, it was this understanding that led to some to adapt Craig's counter-current distribution model for discrete processes to HSCCC which possesses genuine chromatographic profiles [6]. Amongst other restrictions, such adaptation can become possible only when thorough phase mixing and settling over each rotation has been achieved. As a guidance and clarification, throughout this paper readers are referred to Fig. 1 for 3 column geometries to be discussed and compared.

In the period 1984–1986, Conway and Ito [7], Conway et al. [8,9] and Sutherland and Heywood-Waddington [10] reported stroboscopic observation results on 2-D spiral columns (Fig. 1A) in a few conferences, with abstracts being restrictively available. In Conway's CCC monograph of 1990 were published 4 original black-and-white photos taken under stroboscopic illumination for chloroform-acetic acid-water (2:2:1) two-phase solvent system [11]. For illustrating Conway's findings, Sutherland et al. disclosed 4 original photos for an unknown two-phase system in 2000 [12]. Overall, photographic results for phase behaviour of HSCCC have been sparse in the public domain. Speculatively, the less satisfactory quality of photos taken in the past and hence consideration for their suitability in printed version may well have contributed to this situation.

In 2007, Guan et al. reported digital images taken under stroboscopic illumination to show the anticipated mixing and settling pattern for a PEG-phosphate aqueous two-phase system (ATPS) in a 2-D spiral column undergoing J-type CCC planetary motion [13]. This work was conducted mindful that the 3-D helical column on J-type CCC has a great difficulty in retaining satisfactorily any chosen stationary phase out of polar two-phase systems (typically ATPSs) [14]. In line with existing experimental results, the 2-D spiral model published in 2007 [15] confirmed theoretically that certain flow modes for a 2-D spiral column have the potential in achieving sound stationary phase retention for polar two-phase solvent systems. However, at that time neither experimentally nor theoretically were we able to differentiate the intensity of phase mixing between the 3-D helical and the 2-D spiral columns.

The most compelling feature of HSCCC is the use of a centrifuge to establish hydrodynamic and hydrostatic forces for retaining a liquid stationary phase. Our understanding on the physical working of HSCCC has been improved considerably in recent years [15,16], and we are now able to explain why 3-D helical columns have difficulties in retaining stationary phase for more polar two-phase systems like ATPS and this knowledge advancement inevitably sheds light on application scope that the solvent selection approach per se could achieve (e.g. ref. [17]).

Against such a backdrop, the objective of the present work was to observe, using stroboscopic illumination, the dynamics of an ATPS for a 2-D spiral column under all the 8 flow modes. It was further endeavoured to address the following aspects, (a) to observe the effect of J-type planetary motion for hydrodynamic phase distribution in the 2-D spiral column, (b) to observe the hydrodynamic phase behaviour during a transitional period, which leads to dynamic balance between stationary phase retention and mobile phase flow, (c) based on existing experimental results, to make systematic observations for the mixing and settling pattern, and (d) finally to amend and generalise the presently existing rule-of-thumb for determining mobile phase flow orientation based on head and tail locations. Most of colour digital images taken will be left as Supplementary materials at Elsevier Publisher website in JPEG format and thus further uses of these original experimental results are possible.

## 2 Materials and methods

### 2.1 The ATPS

The ATPS was composed of 18% (w/w) polyethylene glycol (PEG) 1000 (Sigma–Aldrich P3515) and 18% (w/w) K_2_HPO_4_ (Sigma–Aldrich P3786) in deionised water. This ATPS was prepared by dissolving 180 g of PEG 1000 and 180 g of anhydrous dibasic potassium phosphate in 640 g of distilled water aided by a magnetic stirrer at 30 °C. The lower phase is rich in phosphate and the upper phase is rich in PEG. The ATPS was equilibrated to 25 °C, mixed and allowed to phase separation.

The phase system has a volume ratio of the upper phase to the lower phase close to one. For this ATPS, the settling time is 51 s, density difference of the two phases 130 kg/m^3^, interfacial tension 2.76 mN m^−1^, upper phase viscosity 18.3 mPa s, and lower phase viscosity 2.14 mPa s [13,18]. The upper PEG phase was coloured blue using Cibacron blue 3G-A (Sigma C9534) alone or green with a combination of Cibacron blue 3G-A and crocin (Fluka 17304). Because of the unilateral partitioning of most dyes in this type of ATPS, the lower phase appeared to be colourless under daylight and greyish in the colourful digital images taken under the stroboscopic lighting described below.

### 2.2 The J-Type CCC system

**Fig. 2:**
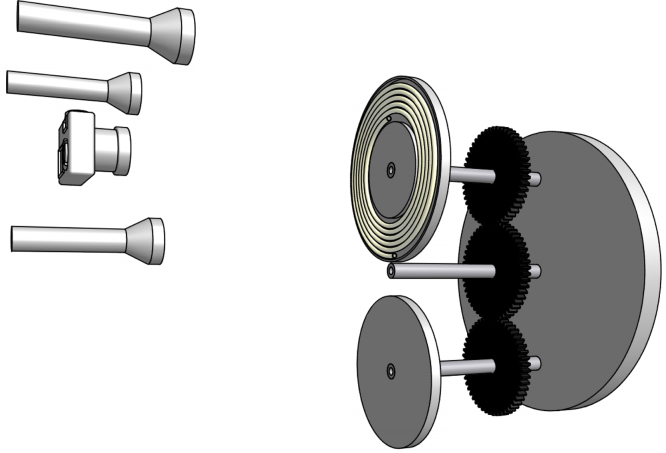
Experimental set-up for studying the phase behaviour of an aqueous two-phase system in a J-type CCC centrifuge with a 2-D spiral column. This CCC centrifuge was kept in a dark room, and the 2-D spiral column or a part of it in motion become static by synchronising the flashing frequency of 3 synchronised strobe lights. A digital camera then took those static images.

The experimental set-up highlighting key hardware parts is illustrated in Fig. 2. A custom-made J-Type CCC device was made by modifying an MSE benchtop centrifuge. An electrical heater kept the space inside the centrifuge casing at 25 °C, with the temperature sensor being located on the internal side of the metal casing. The centrifuge was turned 90° on its axis so that the originally vertical rotor shaft became horizontal and the top of the original centrifuge became the vertical front face of the present J-Type CCC centrifuge. This front was covered by a 15-mm thick transparent Perspex panel to separate the rotor from its surrounding. A solid metal base was welded to the lower part of the present CCC centrifuge for stability during rotating operation. An in-house made grooved aluminium disc covered by a 5-mm thick Perspex panel tightly housed 2.37-m long, 5.5-turn spirally wound semi-transparent fluorinated ethene propene (FEP) tubing (I.D. 5 mm, O.D. 6 mm), which covered the *β* value from 0.55 to 0.90. The *β* value is the ratio between the radial distance from the column to the axis of the planetary disc (*r*) and the distance from the planetary axis to the axis of the rotor (*R*). The radius from the solar axis to the planetary axis is *R* = 10.2 cm [13,19]. The 2-D spiral column for this work expands clockwise. Another aluminium disc with the same weight was used as the counterbalance to this CCC column holder.

Two Gilson 100-ml HPLC pumps were used to deliver the stationary phase before and during the column rotation. With the centrifuge rotating at 800 rpm either clockwise or counter-clockwise, the designated mobile phase was pumped into the column either from centre or from periphery of the 2-D spiral column. Dynamic equilibrium between the two phases (if any) is achieved following a transitional period, which ends when stationary phase stops being co-eluted with mobile phase. The displaced stationary phase by mobile phase was collected in a measuring cylinder and the stationary phase volume retained in the column was thus calculated using the prior measured column volume, extra-column volume, and the stationary phase volume displaced by mobile phase. Stationary phase retention (*S*_f_) is thus quantified as the percentage, to the CCC column volume, of the stationary phase volume retained in the column under a dynamic rotating condition. Alternatively, the *S*_f_ values were estimated by analysing static column images shortly after the centrifugal rotation and mobile phase flow were simultaneously terminated, either manually or with the aid of software AutoCAD^®^ for a specific flow mode and a mobile phase flow rate.

**Fig. 3:**
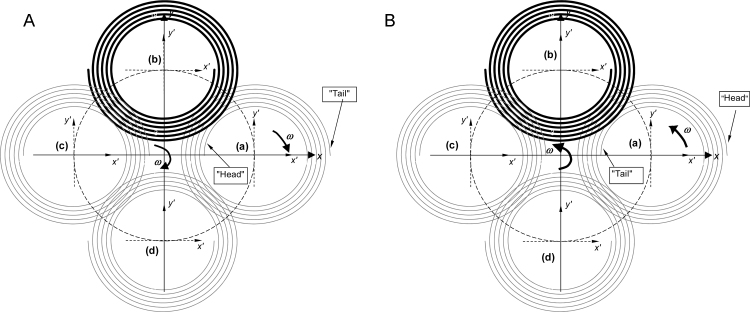
An illustration of the 2-D spiral column undergoing J-type synchronous planetary motion. For the present work, the stroboscopic images were taken from position (b), (A) with clockwise rotation, and (B) with counter-clockwise rotation.

**Fig. 4:**
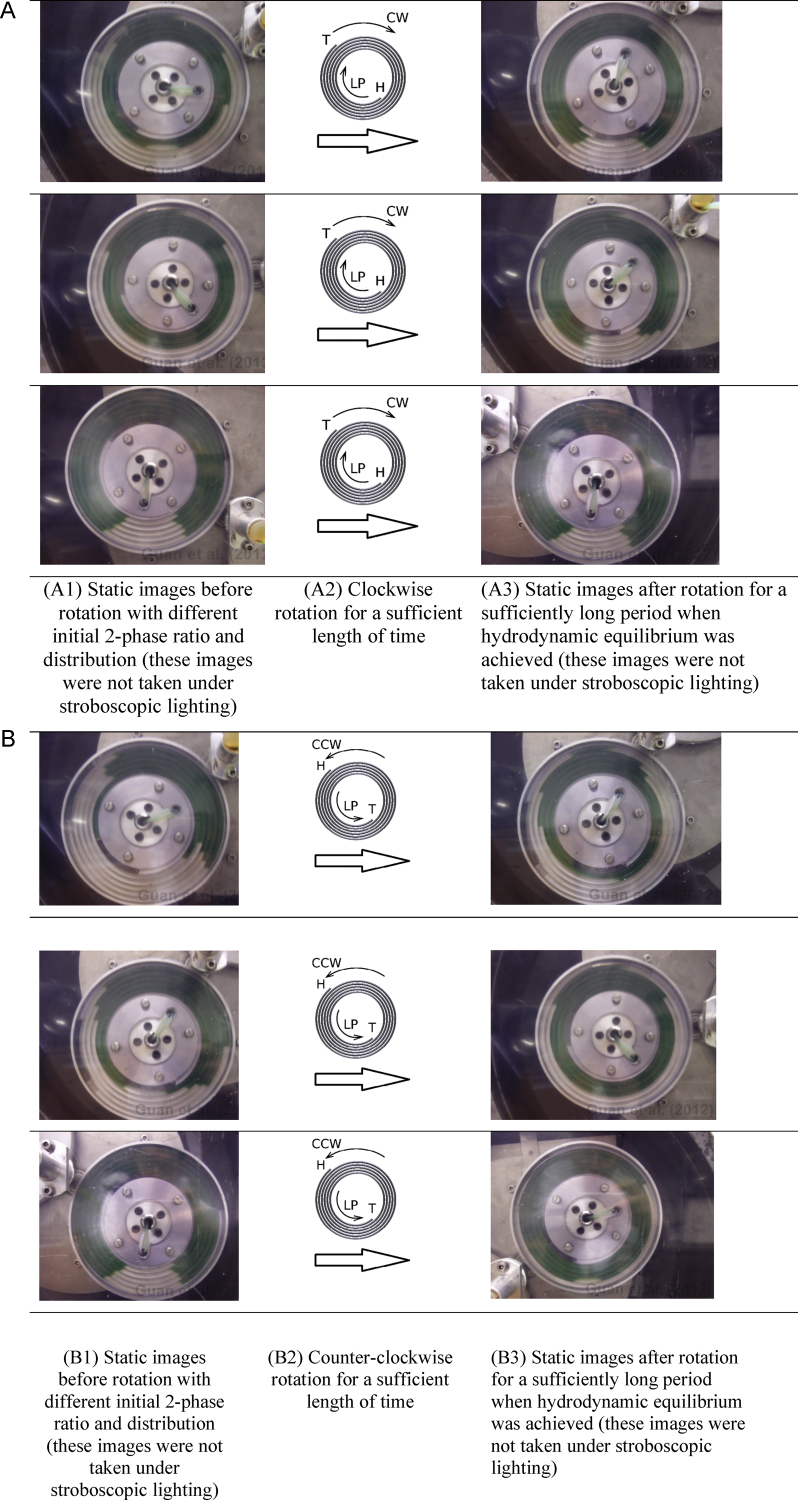
Six sets of observation of the effect of J-type planetary motion alone on hydrodynamic distribution of the PEG-phosphate ATPS in a 2-D spiral column. (A) clockwise rotation (tail at periphery), and (B) counter-clockwise rotation (tail at centre). The ATPS system is composed of 18% (w/w) PEG 1000 and 18% (w/w) K_2_HPO_4_ in water. Rotation speed of the CCC centrifuge leading to hydrodynamic re-distribution of the ATPS was at 800 rpm. There was no external pumping of either phase of the ATPS. The electronic version of this paper has coloured photos, in which the upper PEG phase is in green and the lower phosphate phase in whitish grey, and so the results are more discernible in the PDF colour version.

Table 1 details the 8 elution modes unique to the 2-D spiral column (see Figs. 3 and 4). Different to the 3-D helical column, a 2-D spiral column can have its “head” (or “tail”) terminal either at “centre” and “periphery”, and therefore can have 8 flow modes. In contrast, a 3-D helical column possesses only 4 flow modes for selection.

### 2.3 The lighting and imaging system

The CCC centrifuge was placed in a dark room and 3 strobe lights (Flash Tac transistored stroboscopes, manufactured by Electronic Applications Commercial Limited, London, England) were used for making static observation of the liquid phases inside the rotating column. A set of professional lighting frame was used to position the 3 stroboscopes so that they were located roughly 120° apart on a vertical plane in the front of the CCC centrifuge. Each stroboscope illuminated the cyclically moving target inside the column at an observation angle of 45° and using a flash frequency synchronised with the centrifuge rotation frequency (i.e. ca. 13.3 Hz). An aluminium marker flag was mounted onto the rotor of the centrifuge and was paired with a reflective infrared proximity sensor located on the internal side of the centrifuge casing for automatically detecting the rotation speed. This detection assembly generated an electronic pulse at each rotor revolution and the pulse signal was then amplified via an output stage for automatically triggering the master stroboscope at the exact moment when this “flag” was detected. The other two stroboscopes were synchronised with this master stroboscope through passive signalling control. As the centrifuge was rotated at 800 rpm, the strobe lights illuminated the column using intermittent short flashes at a frequency of 800 times per min (Fig. 2). Consequently, the column or a focussed part of it then appeared to be stationary to eyes and can be visually recorded as static images by a digital camera. When consecutive digital images were taken at a fixed time interval (0.5 or 1-min interval), the hydrodynamic phase re-distribution process for reaching a dynamic equilibration can thus be observed visually.

A RICOH Caplio R1v digital CCD camera (with a shutter release time lag of 0.05 s) was positioned on a tripod and digital images were typically taken at a viewing angle of 0° from a distance of 17 cm to the centrifuge front. Under stroboscopic lighting, the camera focussed either on the entire 2-D spiral column or on a section of it. Use of the in situ camera flash was not permitted in this work. When necessary, images were automatically captured by the camera at an interval of 30-s or 1-min for up to 1 h.

## 3 Results

Where possible, digital images under the room lighting were taken to record the effect of clockwise or counter-clockwise rotation on hydrodynamic phase distribution of the ATPS in the absence of mobile phase flow. The so-called room lighting is much less satisfactory than a daylight condition (see e.g. Fig. 4 A1, A3, B1 and B3). This is partially because our emphasis was given to an environment suitable to stroboscopic illumination. Rotation direction of the centrifuge determines the location of the CCC “head” and “tail” terminals. Digital images were taken for all the 8 flow modes to observe the transitional process of stationary phase replacement (either partially or completely) by mobile phase. For the L-I-H flow mode which well retains the stationary phase, a close observation using digital images was made on virtually all the parts of the 2-D spiral column for the two phase interaction.

### 3.1 Effect of centrifuge rotating direction on phase distribution in the absence of mobile phase flow

Fig. 4 shows that rotating direction (i.e. either clockwise or counter-clockwise) of this J-type CCC centrifuge results in different outcome of hydrodynamic phase distribution. All three A3 photos of Fig. 4 show that, when the head is at the centre, some lower phase (clear) is present in all loops of the column (coil) at the end of the rotation period, thereby implying bilateral spreading of the lower phase. In contrast, the three B3 photos of Fig. 4, with the tail at the centre, show that the inner loops of the column (coil) are completely filled with upper (blue) phase, whilst the outer loops (head) contain only lower (clear) phase. Counter-clockwise rotation clearly pushes the lower phosphate phase to the periphery of the 2-D spiral column (Fig. 4B). During clockwise rotation, the lower phase clearly occupies the periphery, but it cannot be removed from the upper phase occupying the region near the central terminal (Fig. 4A). In Fig. 4 the initial phase loading pattern and amounts of each phase were deliberately made different, but their re-distribution patterns subsequent to a sufficiently long period of J-type planetary rotation have not been affected by these initial conditions.

### 3.2 Effect of flow mode and mobile phase flow rate on stationary phase retention

**Fig. 5:**
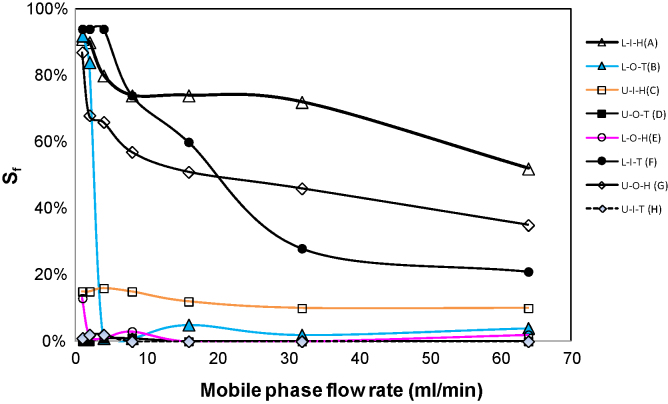
Experimentally measured stationary phase retention (*S*_f_) for different flow modes and at different mobile phase flow rates for the 2-D spiral column with the ATPS. Results for different flow modes are more discernible in the PDF colour version.

Measurements were conducted for stationary phase retention for all the 8 flow modes described in Table 1 at mobile phase flow rates of 1 ml/min, 2 ml/min, 4 ml/min, 8 ml/min, 16 ml/min, 32 ml/min and 64 ml/min respectively. Over the flow rates tested (with the results being summarised in Fig. 5), decent stationary phase retention was obtained for the L-I-H, U-O-H and L-I-T flow modes. The L-I-H flow mode has the highest stationary phase retention in the flow rate range tested. Between the L-I-T and U-O-H flow modes, stationary phase retention for the former is much more sensitive to the mobile phase flow rate. When mobile phase flow rate is less then ca. 20 ml/min, the L-I-T has stationary phase retention higher than the U-O-H flow mode, but this is reversed when the mobile phase flow rate becomes greater than ca. 20 ml/min.

The L-O-T flow mode has a peculiar performance: at mobile phase flow rate up to 2 ml/min its stationary phase retention has been very satisfactory. However, somewhere over mobile phase flow rate 2–4 ml/min, stationary phase retention tumbles to merely a few percent. The U-I-H flow mode is associated with a low yet robust level of stationary phase retention. The U-O-T, L-O-H and U-I-T flow modes show no sign for stationary phase retention regardless of the mobile phase flow rate might be reduced.

### 3.3 Mobile phase replacing stationary phase in transitional period: the dynamics

**Fig. 6:**
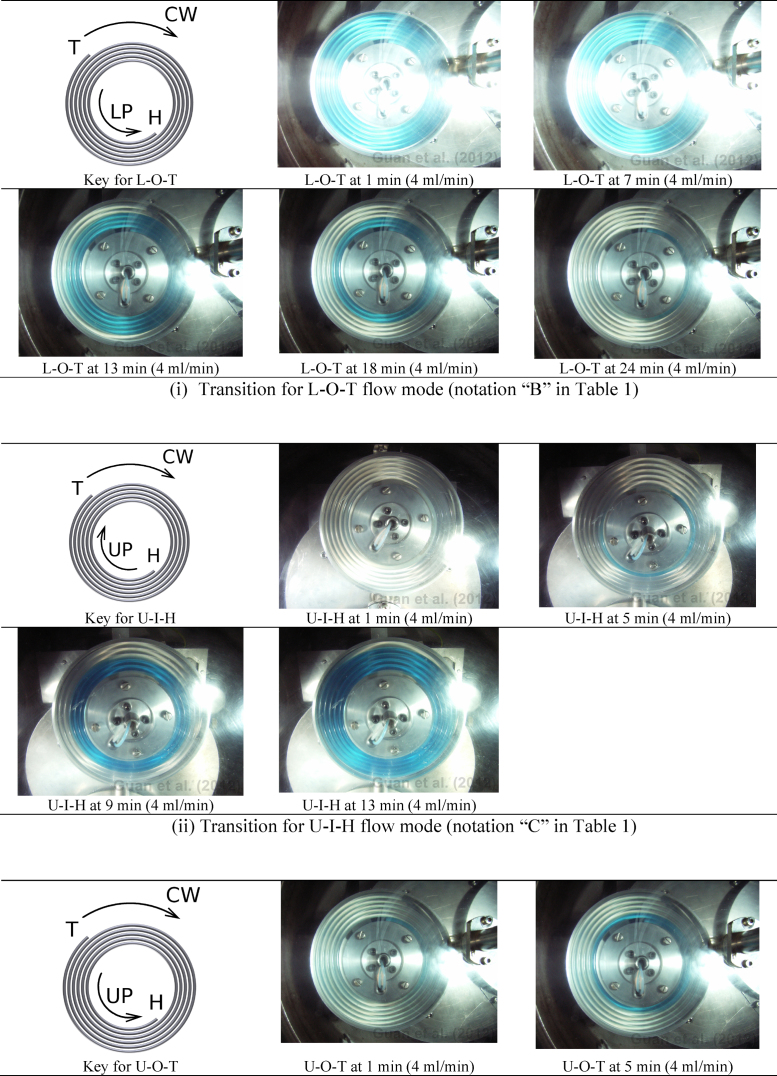

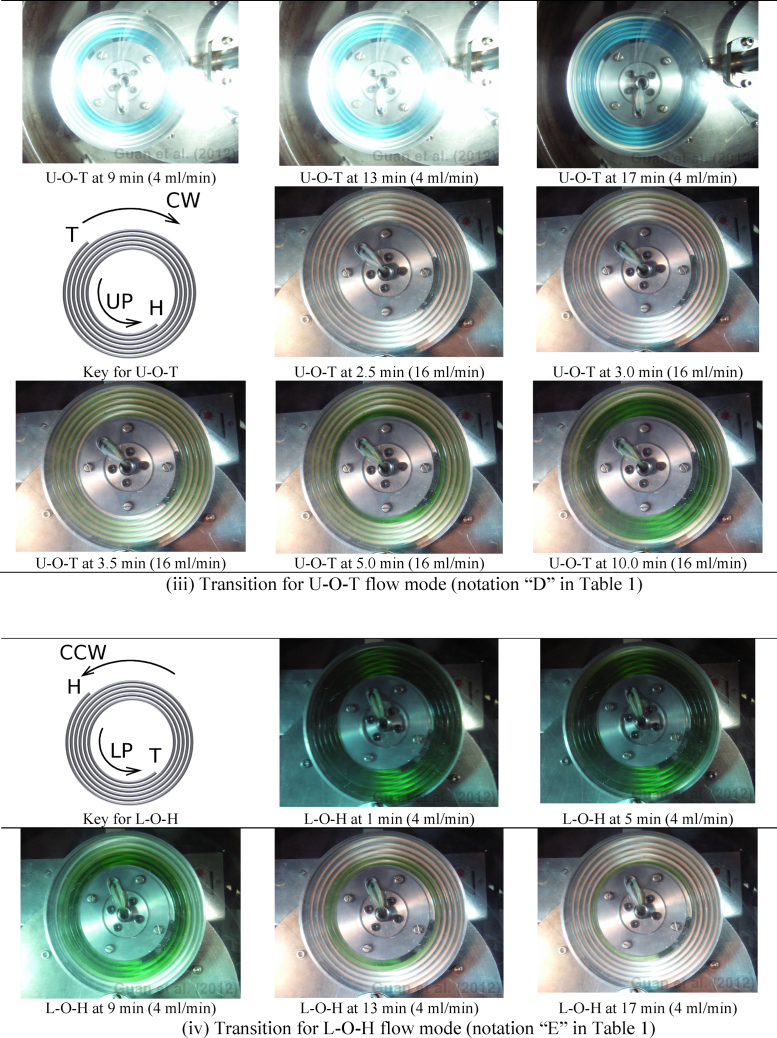

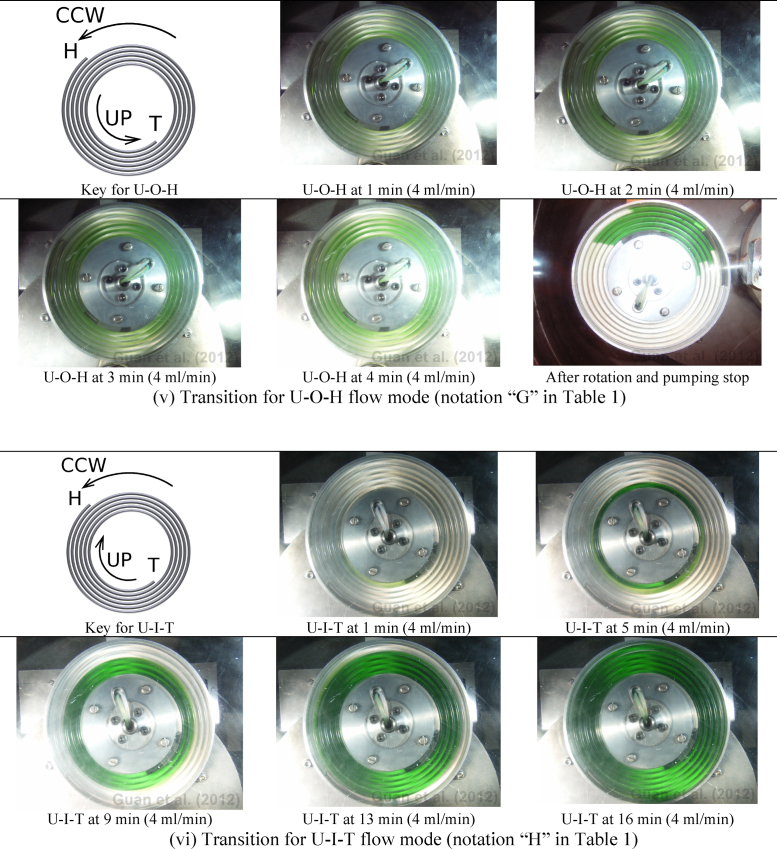
Snapshots at different times of the transitional periods with 4 ml/min mobile phase flow rate for 6 flow modes where at least majority of the intended stationary phase was replaced by the mobile phase in the 2-D spiral column. At the beginning of a transitional period, the 2-D spiral column was filled with the intended stationary phase. Rotation of the J-type CCC centrifuge at 800 rpm was shortly followed by initiation of the mobile phase flow using an external HPLC pump. Results are more discernible in the PDF colour version. All the detailed results are shown respectively in S2, S3, S4(part1), S4(part2), S5, S7 and S8 (Supplementary material).

The phase replacement dynamics for L-O-T, U-I-H, U-O-T, L-O-H, U-O-H and U-I-T flow modes were briefed in Fig. 6. At the beginning of a dynamic process, the 2-D spiral column was invariably filled with stationary phase. Observation was made using digital images which were taken under the stroboscopic illumination at a constant time interval. More imaging results in JPEG format were given in Supplementary materials 1 through 8 (i.e. S1–S8) to the present paper (including the results for L-I-H and L-I-T flow modes). It is hoped that these JPEG-format results will withstand further analyses and/or comparisons.

When the lower phase was pumped into the 2-D spiral column from periphery, Fig. 6(i) (for L-O-T) and Fig. 6(iv) (for L-O-H) show that the upper phase is squeezed out of the column in the periphery-to-centre direction. When the upper phase is pumped from the central terminal into the column initially filled with the lower phase, Fig. 6(ii) (for U-I-H) and Fig. 6(vi) (for U-I-T) show that the lower phase (as stationary phase) is squeezed out of the 2-D spiral column in the centre-to-periphery direction. Fig. 6(iii) (for U-O-T) and 6(v) (for U-O-H) show that, when the upper mobile phase is pumped into the 2-D spiral column from the peripheral terminal, it swiftly traverses the column longitudinally to reach the central terminal. For the U-O-T flow mode, once the upper phase reaches the central terminal, it then expands its volume in the centre-to-periphery direction (i.e. opposite to its flow direction) and the lower stationary phase is gradually yet eventually completely squeezed out of the column in the periphery-to-centre direction. In this phase replacement process, the lower stationary phase is removed from the central terminal which is predominantly occupied by the upper mobile phase. For the U-O-H flow mode, the phase movement pattern is similar to that of the U-O-T mode except that the lower stationary phase is not proportionally removed by the incoming upper mobile phase and hence decent stationary phase retention is achieved [Fig. 6(v) and S7].

The L-I-H and L-I-T flow modes give rise to sound stationary phase retention and as such it is not easy to visualise the greyish lower mobile phase even on colourful digital images, which are documented in S1 and S6 (Supplementary material) respectively.

### 3.4 Phase mixing and settling observation

**Fig. 7:**
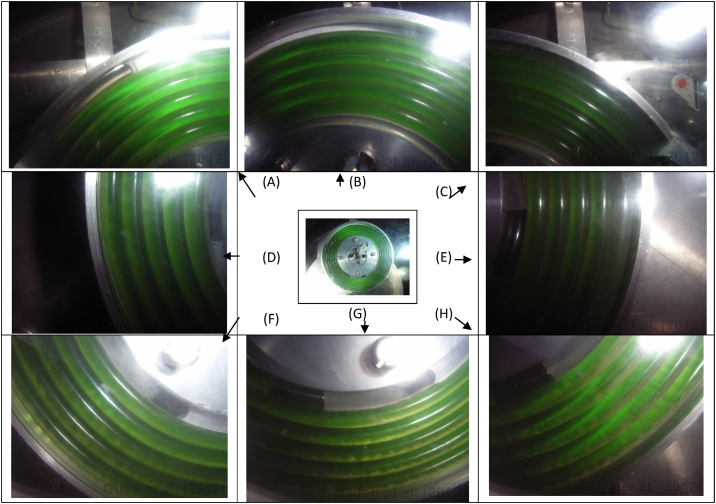
Snapshots at different locations on the 2-D spiral column for L-I-H flow mode. The image in the middle shows the entire 2-D spiral column, and (A) through (B) show selected, focussed parts of the column. Results are more discernible in the PDF colour version.

For the L-I-H flow mode, a series of images were taken to look into details for the dynamic mixing and settling patterns of the ATPS (Fig. 7). In general, phase separation occurs near the distal key nodes (i.e. on the vertically top region of the 2-D spiral column) and wavelike phase mixing occurs near the proximal key nodes (i.e. on the vertically bottom region of the 2-D spiral column) (see the photo in the middle of Fig. 7). Fig. 7 shows only a selection of the results and its complete set is given in S9 (Supplementary material). Near the proximal key nodes, the wavelike mixing teeth appear to be deeper for the external 3 turns of the 2-D spiral than for the internal 2 turns (Fig. 7F–G, or S9 (Supplementary material) at nos. 15, 18, 19, 20, 21 and 23 for more results). In the region clockwise passing the proximal key nodes, the wavelike teeth almost disappear and the two phases seem to be somewhat penetrative with each other.

## 4 Discussion

*The Archimedean screw force and the solid–liquid friction force: a clarification*  

Before unfolding the Discussion, we wish to make a clarification for the terminology, Archimedean screw force. It becomes clear very recently that the definition of Archimedean screw force used by Guan et al. [15] and Guan and van den Heuvel [16] has been different to that by Ito [21]. What Ito meant could be termed as an Archimedean screw effect, and is generated by combination of coil rotation and the forces acting nearly perpendicular to the axis of the rotating coil. This effect (or force) is exactly the physical effect for lifting water from a low position to a high, as documented for a device allegedly designed by Archimedes in Greece some a few thousand years ago. Ito's definition concerns more to the net effect rather than to a clearly defined physical force. For the avoidance of confusion, the present paper has adopted a new terminology “the solid–liquid friction force” to replace the previous terminology “Archimedean screw force” that was used in refs. [15,16].

For the 3-D helical coil with both ends blocked and performing self-rotation, there are hydrostatic and hydrodynamic forces: (a) gravity, (b) centrifugal force (both hydrostatic forces), and (c) solid–liquid friction force (a hydrodynamic force). When self rotation speed of the coil is very slow (to say, 1 rpm), centrifugal force [Force (a)] and the solid–liquid friction force [Force (c)] can be neglected. This leaves gravity [Force (a)] to be the dominant one. When both ends are blocked, the main hydrodynamic force is the solid–liquid friction force, which is caused by the movement of the column. When there is a mobile phase flow, there will be a second major friction, the liquid–liquid friction force, which is caused by the movement of mobile phase.

When ATPS is constrained in the 2-D spiral column, lower phase tends to move to periphery whereas upper phase to centre and this is so regardless of the rotational directions of the CCC centrifuge (Fig. 4). A change in rotational direction alters the direction of the liquid–solid friction force (a hydrodynamic force), but not the centrifugal force (a hydrostatic force). These results in Fig. 4 thus show that the phase distribution effect caused by the centrifugal force can mostly overcome that by the liquid–solid friction force. Indeed, the present results confirm our previous finding that the centrifugal force (more specifically, the tangential centrifugal force) plays a pivotal role in causing unilateral hydrodynamic phase distribution in 2-D spiral columns [15]. However, the present results (Fig. 4) show that the strength of this phase distribution tendency is markedly different between the two rotational directions (i.e. whether the CCC tail is located at centre or periphery). When the CCC tail is located at the 2-D spiral centre (Fig. 4B), there is a strong bilateral hydrodynamic phase distribution tendency, and so, as expected, both L-I-T and U-O-H flow modes give rise to sound stationary phase retention. Clockwise rotation (i.e. tail at periphery, Fig. 4A) results in lower phase being distributed unilaterally into the periphery, but at the same time and in a consistent fashion cannot result in lower phase depletion from upper phase which prevails in the inner spiral turns starting from the central terminal. These results have indeed implicitly explained the seemingly unexplainable stationary retention fates, as shown in Fig. 5.

In another angle, certain retention results for the 2-D spiral column may appear to be anomalies based on the existing rule-of-thumb for operating CCC. With tail at periphery, when lower phase is used as mobile phase, excellent stationary phase retention can be achieved. However, when upper phase as mobile, the expected U-O-T flow mode cannot retain any level of stationary phase yet unexpectedly U-I-H has robustly retained a low level (≥10%) of stationary phase at mobile phase flow rate up to 64 ml/min. Interestingly, with tail at centre, experimental results have been exactly what one would expect. Amongst the other results, the contrasts for stationary phase retention between U-O-T and U-O-H, and between U-O-T and U-I-H, (a) have utterly breached the well established rule for running 3-D helical column CCC [1] and (b) perhaps indicate contradictions with the present understanding on the effect of tangential centrifugal force on the 2-D spiral column [15]. In the following, we wish to examine these two perspectives.

(a) Evaluation of the prevailing CCC head-and-tail rule  
  This rule-of-thumb for operating HSCCC can be summarised as follows. For the 3-D helical column, “a lower (heavier) mobile phase should be introduced through the head towards the tail, and an upper (lighter) mobile phase in the opposite direction” [1]. For the 2-D spiral column, it was further suggested that, “if the lower phase is mobile, pump from head (centre) to tail (periphery) and if the upper phase is mobile, pump from tail (periphery) to head (centre)” [22]. It is worth pointing out that the multi-layer 3-D helical column (Fig. 1C) is equivalent to its single-layer counterpart (Fig. 1B), except that there is a small (thus usually negligible) 2-D spiral column element at both ends where one layer of 3-D helical layer moves to the next. The foundation for these rules has been a long-held hypothesis that the Archimedean screw force (effect) exerts on the lower phase in the head-to-tail direction whereas on the upper phase in the tail-to-head direction. However, it is not difficult to select two-phase systems to breach this empirical rule. For operating J-type CCC centrifuge possessing 2-D spiral columns, this rule has completely failed in providing practical guidance to end users.
(b) Evaluation of the 2-D spiral model by Guan et al.  
  Based on the 2-D spiral model developed earlier [15], when the lower phase flows in the centre-to-periphery direction (i.e. the L-I-H and L-I-T flow modes), the lower mobile phase is expected to quickly move to the periphery. Experimental results in Fig. 6(ii) (for the U-I-H flow mode) as well as in S. 6 (Supplementary material) clearly support such mechanism. It is worth pointing out that observing such results for L-I-T flow mode is difficult, because high stationary phase retention allows only a small percentage of coloured stationary upper phase being replaced by the colourless lower phase [see S. 6 (Supplementary material)]. In the same vein, when the upper phase flows in the periphery-to-centre direction (i.e. U-O-T and U-O-H flow modes), the upper phase is expected to quickly move to the centre. Experimental results in Fig. 6(iii) (for U-O-T) and Fig. 6(v) (for U-O-H) unambiguously allow us to visualise this process. Experimental results for L-O-T, U-I-H, L-O-H and U-I-T can also be explained apropos by the effect of tangential centrifuge force. To summarise, whether upper phase or lower phase is pumped into the 2-D spiral column from spiral centre or periphery, invariably lower phase immediately and predominantly occupy the spiral periphery terminal, and upper phase the central terminal. These results (including those in Fig. 5) show not only the role that tangential centrifugal force plays inside the 2-D spiral column [15], but also indicate a delicate interplay of the other forces (most significantly the solid–liquid friction force) in determining hydrodynamic stationary phase retention.

We maintain that the 2-D spiral model remains intact in interpreting the experimental results hitherto, but the conventional didacticism on Archimedean screw force has not focussed on all the important physical forces. The previous analyses elucidated a differentiated effect of the solid–liquid friction force on upper and lower phases respectively, and on the working of the tangential centrifugal force [15]. The present work allows us to visualise the detailed phase replacement dynamics. We have taken this opportunity to reconcile most (if not all) of the conflicting experimental results in the literature where the integrity of the existing rule-of-thumb fails to be upheld. In the following, we intend to provide a unified explanation to embrace all the experimental results documented.

The solid–liquid friction force exerts on both lower and upper phases in the head-to-tail direction, but usually more on the lower phase owing to the effect of normal centrifugal force. This difference results in a relatively strong drag force on lower phase in the head-to-tail direction yet a weak drag force on upper phase in the same direction. For the 3-D helical column, this normal centrifugal force is nearly identical to net centrifugal force resulted from the J-type CCC planetary motion (but see ref. [16]). For the 2-D spiral column, centrifugal force can be separated orthogonally into normal and tangential centrifugal forces. A considerable difference in density between lower and upper phases is required in order to differentiate the effect of the solid–liquid friction force on the two phases. Furthermore, low interfacial tension is expected to reduce such differentiation on the two phases. Liquid phase slippery along the inner wall surface of the CCC column under a high rotational speed means that both phases have the tendency for being thrown out the column from the head terminal! This is a most confusing concept in this research field. An outcome of the existing understanding has unsurprisingly left anomalies for certain two-phase systems unexplainable.

For physical property of phase systems, we regard two factors, density difference between lower and upper phases and interfacial tension, as being the most important. The latter is related to polarity of phase systems and manifested by settling time for phase separation.

For 3-D helical columns, retention of a designated stationary phase relies entirely on the solid–liquid friction force. As such, anomalies for phase distribution orientation and stationary phase retention can occur only if a discernible differentiation of normal centrifugal force on upper and lower phases cannot be realised. For 2-D spiral columns, the solid–liquid friction force (a drag force) can work in the same direction as the tangential centrifugal force, however these two forces can also work against each other. When the two forces act in the same direction (i.e. tail at periphery of the 2-D spiral column), the tangential centrifugal force pushes lower phase preferentially to periphery of the spiral column and usually the solid–liquid friction force also drags lower phase preferentially to periphery. Consequently, lower phase should usually flow in head (centre) to tail (periphery) direction (i.e. the L-I-H flow mode), whereas upper phase in tail (periphery) to head (centre) direction (i.e. the U-O-T flow mode). Whilst the first part of this statement can be upheld by experimental results, the second part cannot (Fig. 5).

A reduction in density difference can have two effects. First, it reduces the influence of the tangential centrifugal force and so leads to a weakened tendency for lower phase to move to periphery and upper phase to centre. Second, it reduces the effect of normal centrifugal force and therefore weakens the differentiation of the solid–liquid friction force between upper and lower phases. As usually the normal centrifugal force is much larger than the tangential centrifugal force for 2-D spiral columns [15,16], it is possible that at certain points the solid–liquid friction force can overcome the tangential centrifugal force. The prerequisite for this situation to occur is likely to be for more hydrophobic two-phase systems where interfacial tension is high. For hydrophilic two-phase systems where interfacial tension is very low, the solid–liquid friction force can apply considerably to upper phase and lead to difficulties in separating the two phases due to this drag force.

Based on the presently emphasised nature of the solid–liquid friction force and our understanding for the two physical properties of any two-phase system, certain anomalies for 2-D spiral columns are exemplified and explained in Table 2. The phase systems showing the anomalies concern both hydrophobic and hydrophilic two-phase systems, with the former having longer settling time and the latter shorter settling time. For hydrophobic two-phase systems, these anomalies occur when the solid–liquid friction and tangential centrifugal forces work with each other. For hydrophilic two-phase systems, the anomalies occur when the solid–liquid friction and the tangential centrifugal forces work against each other. Existing and the present experimental results support the following hypotheses:

(a) Density difference (or density ratio) is the key factor leading to “reversed” unilateral hydrodynamic phase distribution.
(b) Interfacial tension, as manifested by settling time, is the key factor resulting in complete loss of stationary phase from a situation where a decent level of stationary phase would have been retained.

For 2-D spiral columns, our reasoning in the present paper well explains why the U-O-T flow mode fails to retain stationary phase for more hydrophilic two-phase systems (Table 2). For 3-D helical columns, this situation simply means that it is not possible to retain a stationary phase out of any phase systems with long settling time. Indeed, panoply of experimental results over the past 3 decades corroborates this reasoning. Results in Fig. 6(iii) for the 2-D spiral column show unambiguously that for ATPS it is the settling time rather than density difference that accounts for the U-O-T retention outcome.

The effect of 3-D helical column bore size on stationary phase retention has been claimed [28], but systematic experimental results have not been available. Our speculation on this factor is as follows. When the internal diameter (i.e. bore size) of a CCC column increases, the effect of the solid–liquid friction force on both phases will proportionally reduce and therefore the relative effect of the tangential centrifugal force becomes more significant. As a more extreme situation for enlarged bore size, tangential centrifugal force might completely separate lower and upper phases irrespective of the tail being at centre or periphery, and indeed the results in Fig. 4 largely show this situation. However, it is less straightforward for the effect of bore size in multi-layer 3-D helical columns. If this effect does exist, our speculation is directed to the 2-D spiral column elements at both ends of the column holder where 2 3-D helical columns inter-connect.

The effect of viscosity has already been reflected in the settling time of a two-phase system. If viscosity is too high, the suitability of a two-phase system for CCC columns will nonetheless prevent it being selected as a working solvent system.

For the sake of simplicity, the above discussion has focussed on high *β* values (i.e. *β* ≥ 0.5) on both the 2-D spiral and 3-D helical columns. Extension of *β* values to a low-value range should not affect the validity of the tangential and normal centrifugal forces, but can slightly complicate the effect of the solid–liquid friction force. The present study does not intend to address it in detail. By all means, the hydrostatic gravity force can be convincingly ignored for HSCCC and as a rough indication this typically means centrifugal rotational speed over 200 rpm [4].

In order to widen applications for the CCC technology, a considerable devotion in the CCC research area has been given to rationalise two-phase solvent systems for not only matching the polarity of the target compounds to be separated but also for searching for adequate stationary phase retention. This work has unravelled inherent limitations of J-type CCC for the presently popular column geometries. For certain flow modes of the 2-D spiral column, stationary phase retention is always low regardless of how much the mobile phase flow rate might be reduced. Likewise, multi-layer 3-D helical columns on HSCCC cannot retain any stationary phase of an ATPS to a satisfactory level at whatever mobile phase flow rate. It is therefore inconceivable that these limitations can be overcome by selecting a “super” solvent system or by manoeuvring to an optimised operation condition.

## 5 Conclusions

CCC technology has advanced considerably using less polar aqueous-organic two-phase systems in the past decades, but suffered from persistent failure when adopting polar two-phase systems (typically ATPSs). Using an ATPS, this work made observation on phase distribution orientation and stationary phase retention processes in a 2-D spiral column operated on J-type HSCCC.

Based on the results of this work and those in the literature, we have examined the roles that each relevant physical force plays in contributing to phase distribution and retention in CCC columns. This in-depth analysis led us to identify that the effects of these concerned forces (the solid–liquid friction force and tangential centrifugal force) can be related to two physical properties of any chosen two-phase system, namely density difference between lower and upper phases and interfacial tension.

This finding then enabled us to connect virtually all the experimental results to this reasoning on the working of J-type CCC on both 2-D spiral and 3-D helical columns. Subject to critiques from within and beyond this research area, it is hoped that we have been close to fully understand the physical nature of the CCC technology invented by Ito some 40 years ago. Conclusions of this work allow us to eventually understand certain boundaries that experimental effects endeavoured to break over the past 20–30 years.

On one hand, based on both theoretical and experimental efforts in the past, this work advanced our knowledge on the physical mechanism of the CCC technology. With minor modifications, the methodology developed here can be extended to understand the other situations for the CCC technology. On the other hand, whilst defining the application boundary for the presently popular forms of the CCC columns, this work may have opened a new avenue for applying this technology to a wide range of applications in bio-science and bio-industry (e.g. [29]).

## Supplementary materials

Supplementary materials 1 through 8 record the transitional phase replacement processes at mobile phase flow rate at 4 ml/min for flow modes L-I-H, L-O-T, U-I-H, U-O-T (for both 4 ml/min and 16 ml/min), L-O-H, L-I-T, U-O-H, U-I-T. Supplementary material 9 records focussed dynamic phase interaction details for L-I-H flow mode at various column locations. We maintain that the results given as Supplementary material are an integral part of this paper. In view of the length and their nature as colour photographs, we feel that the present means for showing these results has been adequate and effective.

## Figures and Tables

**Table 1:** Experimental coverage for all the elution modes and selected mobile phase flow rates for the spiral column on a J-type CCC centrifuge for conducting CCC in the present work.

Detail of flow modes for a spiral column on type-J CCC	“Tail” at the Peripheral (clockwise rotation)				“Head” at the Peripheral (counter-clockwise rotation)			
	Stationary phase: upper PEG; mobile phase: lower phosphate		Stationary phase: lower phosphate; mobile phase: upper PEG		Stationary phase: upper PEG; mobile phase: lower phosphate		Stationary phase: lower phosphate; mobile phase: upper PEG	
	Mobile phase flow centre → periphery (i.e. H→T)	Mobile phase flow periphery → centre (i.e. T→H)	Mobile phase flow centre → periphery (i.e. H→T)	Mobile phase flow periphery → centre (i.e. T→H)	Mobile phase flow periphery → centre (i.e. H→T)	Mobile phase flow centre → periphery (i.e. T→H)	Mobile phase flow periphery → centre (i.e. H→T)	Mobile phase flow centre → periphery (i.e. T→H)
Illustration								

Ito's notation [20]	L-I-H	L-O-T	U-I-H	U-O-T	L-O-H	L-I-T	U-O-H	U-I-T
Conway's notation [11]	L-(H)→T	L-T→(H)	U-(H)→T	U-T→(H)	L-H→(T)	L-(T) →H	U-H→(T)	U-(T)→H
Mobile phase: 1 ml/min	A1	B1	C1	D1	E1	F1	G1	H1
Mobile phase: 2 ml/min	A2	B2	C2	D2	E2	F2	G2	H2
Mobile phase: 4 ml/min	A3	B3	C3	D3	E3	F3	G3	H3
Mobile phase: 8 ml/min	A4	B4	C4	D4	E4	F4	G4	H4
Mobile phase: 16 ml/min	A5	B5	C5	D5	E5	F5	G5	H5
Mobile phase: 32 ml/min	A6	B6	C6	D6	E6	F6	G6	H6
Mobile phase: 64 ml/min	A7	B7	C7	D7	E7	F7	G7	H7

**Table 2:** Example and explanation of hydrodynamic phase distribution anomalies in spiral columns on J-type CCC.^a^

Phase system	Physical property of phase system	Anomaly in hydrodynamic phase distribution	Explanation
(i) Heptane/water (1:1); (ii) Heptane/ethyl acetate/methanol/water (1.4:0.6:1:1); (iii) Heptane/ethyl acetate/methanol/water (1.4:2:1:1) see ref. [12]	(i) More hydrophobic, density difference 340 kg/m^3^, density ratio 1.52, interfacial tension (*τ*) 52 mN m^−1^ (ii) More hydrophilic, density difference 230 kg/m^3^, density ratio 1.33, *τ* = 6.2 mN m^−1^ (iii) More hydrophilic, density difference 138 kg/m^3^, density ratio 1.18, *τ* = 2.1 mN m^−1^	For phase system (i), J-type planetary motion makes upper phase move to the head and lower phase to the tail irrespective of head/tail being at periphery or centre. However for phase systems (ii) and (iii), phase distribution results are consistent with the existing head and tail rule.	The solid–liquid friction force in phase system (i) is able to overcome the effect of tangential centrifugal force when the two forces counteract, and hence determines phase distribution outcome. High density difference ensures high solid–liquid friction force, as coordinated by high normal centrifugal force. High interfacial tension ensures that the solid–liquid friction force exerts preferentially to lower phase.

PEG1000-K_2_HPO_3_ (18% w/w–18% w/w) ATPS (this work)	Very hydrophilic, density difference 130 kg/m^3^, density ratio 1.13, interfacial tension 2.76 mN m^−1^	(a) With tail at periphery, J-type planetary motion moves LP to the tail at periphery and UP to the head at centre (Fig. 4). However, the LP in the UP region cannot be depleted. With tail at Centre, the LP and UP is completely separated with the former being at head (periphery). (b) At U-O-T flow mode, stationary LP is not retained at all (Fig. 5). Similar results occur for a hydrophilic two-phase system n-butanol/acetic acid/water (4:1:5) as documented in ref. [26].	(a) The ATPS has low interfacial tension and so the solid–liquid friction force applies considerably to UP as well. With tail at periphery, whilst tangential centrifugal force pushes LP to the periphery and UP to the centre, the solid–liquid friction force drags both phases towards periphery and hence fails to completely separate the two phases. With tail at centre, UP is subject to the 2 forces towards centre whereas LP to the 2 forces in the opposite directions. Provided tangential centrifugal force can overcome the solid–liquid friction force, the two phases can then be completely separated. This situation indicates that the ATPS has a sufficiently high density difference. In this vein, one can explain all the results shown in Fig. 4. (b) As shown in Fig. 6(v) and Section 3.3, UP coming from periphery moves quickly to centre due apparently to tangential centrifugal force and against the solid–liquid friction force. Because of the solid–liquid friction force, UP accumulated near the central terminal cannot be expelled and so LP is instead repelled slowly with the upper mobile phase. Eventually, the entire LP in the column is gradually replaced by the UP. Indeed, similar experimental results were reported earlier in refs. [13,22–26]. This outcome results from striking a fine balance for retaining both LP and UP. For n-butanol/acetic acid/water (4:1:5) phase system, addition of NaCl into the phase forming water has reduced the settling time from 38.5 s to 26.5 s [26,27].

a Further examples and the associated phase physical properties are given in refs. [12,18,26,27].

## Appendix A Supplementary data

The authors maintain that the results given as Supplementary material are an integral part of this paper. In view of the length and their colour photographic nature, we feel that the present means for showing these results has been adequate and effective.
