# Supplementary material for: Visualisation of J-type counter-current chromatography: A route to understand hydrodynamic phase distribution and retention
Source: J Chromatogr A. 2012 May 25;1239(15):10–21. doi: 10.1016/j.chroma.2012.03.039 (PMC3405518; doi:10.1016/j.chroma.2012.03.039)
Supplement: Supplementary file 5 [file mmc5.doc]

**Supplementary material 4 (Part 2):**

Dynamic phase replacement visualisation for a PEG-phosphate ATPS, with flow mode U-O-T and at a mobile phase flow rate of 16 ml/min, for the spiral tubing undergoing type-J synchronous centrifugal planetary motion. This experimental condition is denoted as D5 in Table 1.

For the dynamic situation of the spiral column illustrated, the central terminal of the spiral is the CCC column head (head), whereas the peripheral terminal of the column is the CCC column tail (tail). With the lower phosphate phase of the ATPS being used as the stationary phase, the upper PEG phase was pumped externally in the direction from the periphery (i.e. tail) to the centre (i.e. head).

The ATPS constituted was formed by 18% (w/w) PEG 1000 and 18% (w/w) K_2_HPO_4_ in deionised water. The rotation speed of the centrifuge was at 800 rpm.

An illustration is shown in the left column for explaining the dynamic situation where the image in the right column was taken.

| S4-pt2-2  The dynamic image for the whole column  At time t=0.5 min  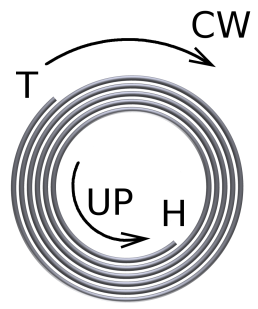 | 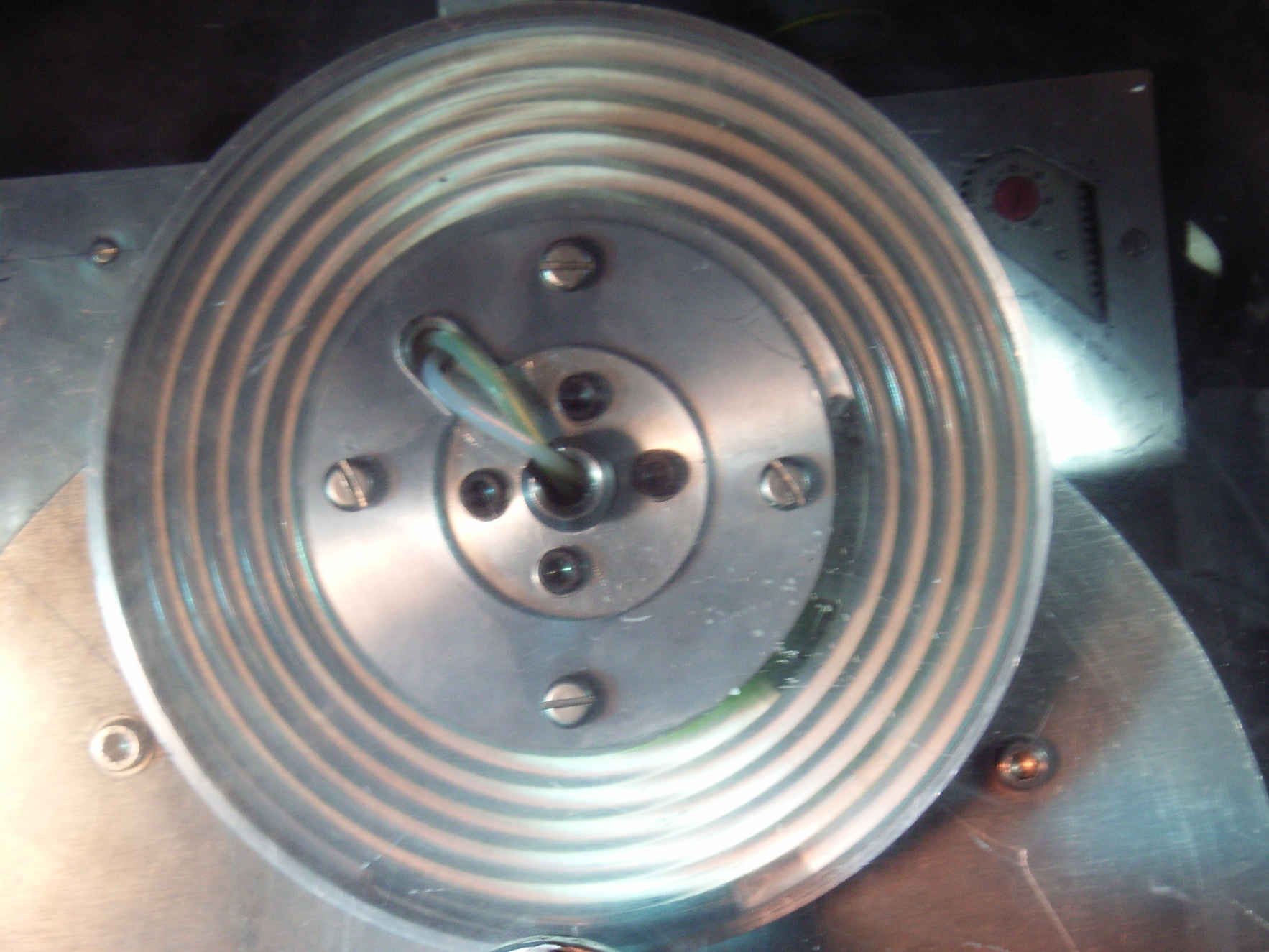 |
| --- | --- |
| S4-pt2-3  The dynamic image for the whole column  At time t=1.0 min  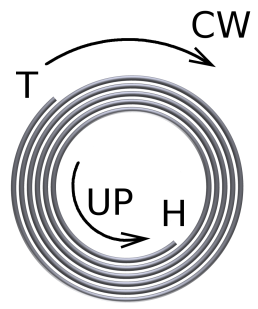 | 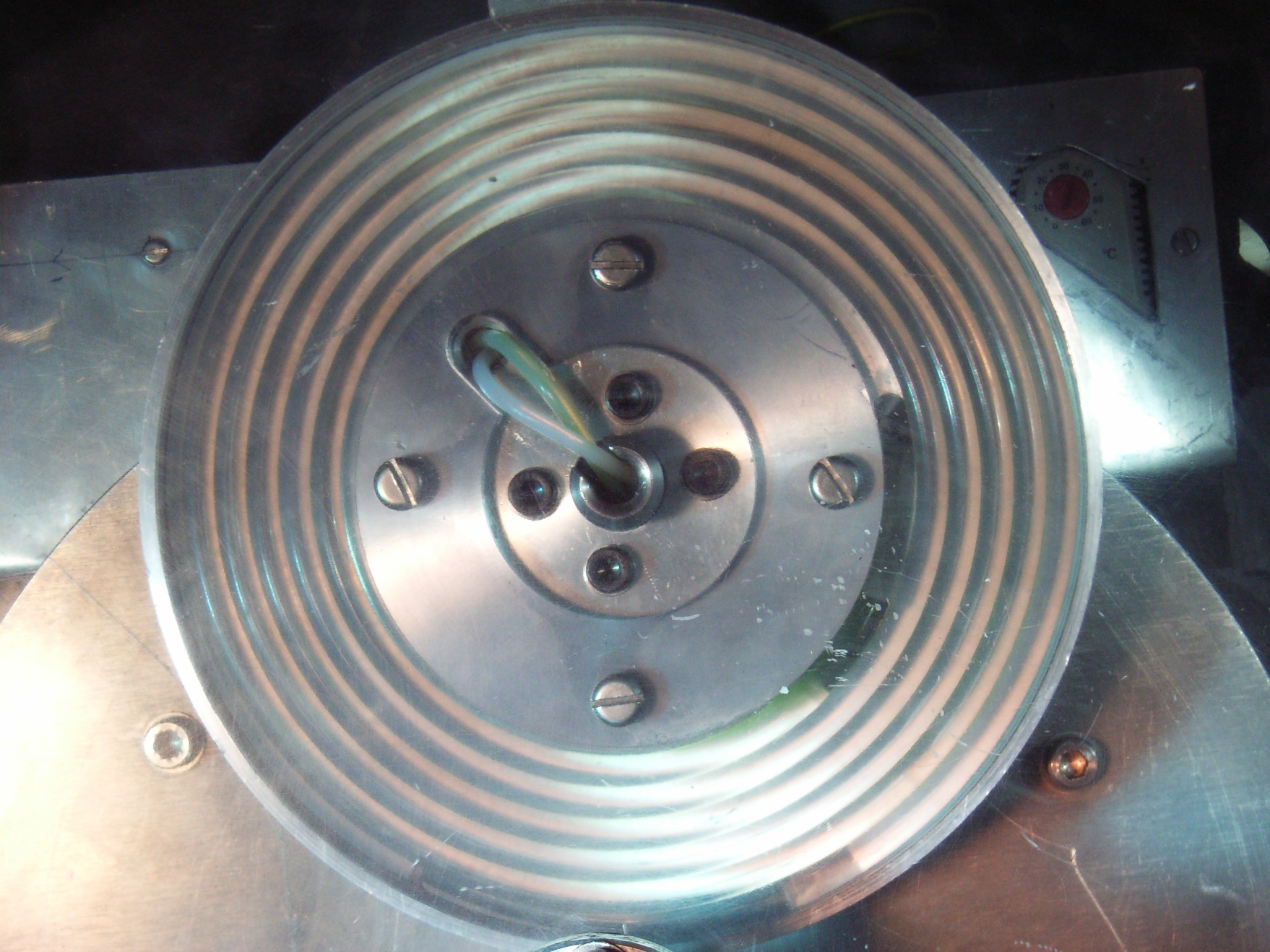 |
| S4-pt2-4  The dynamic image for the whole column  At time t=1.5 min  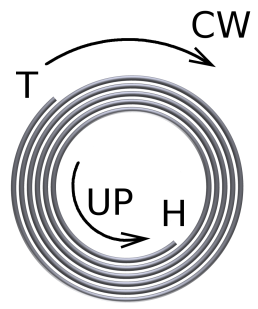 | 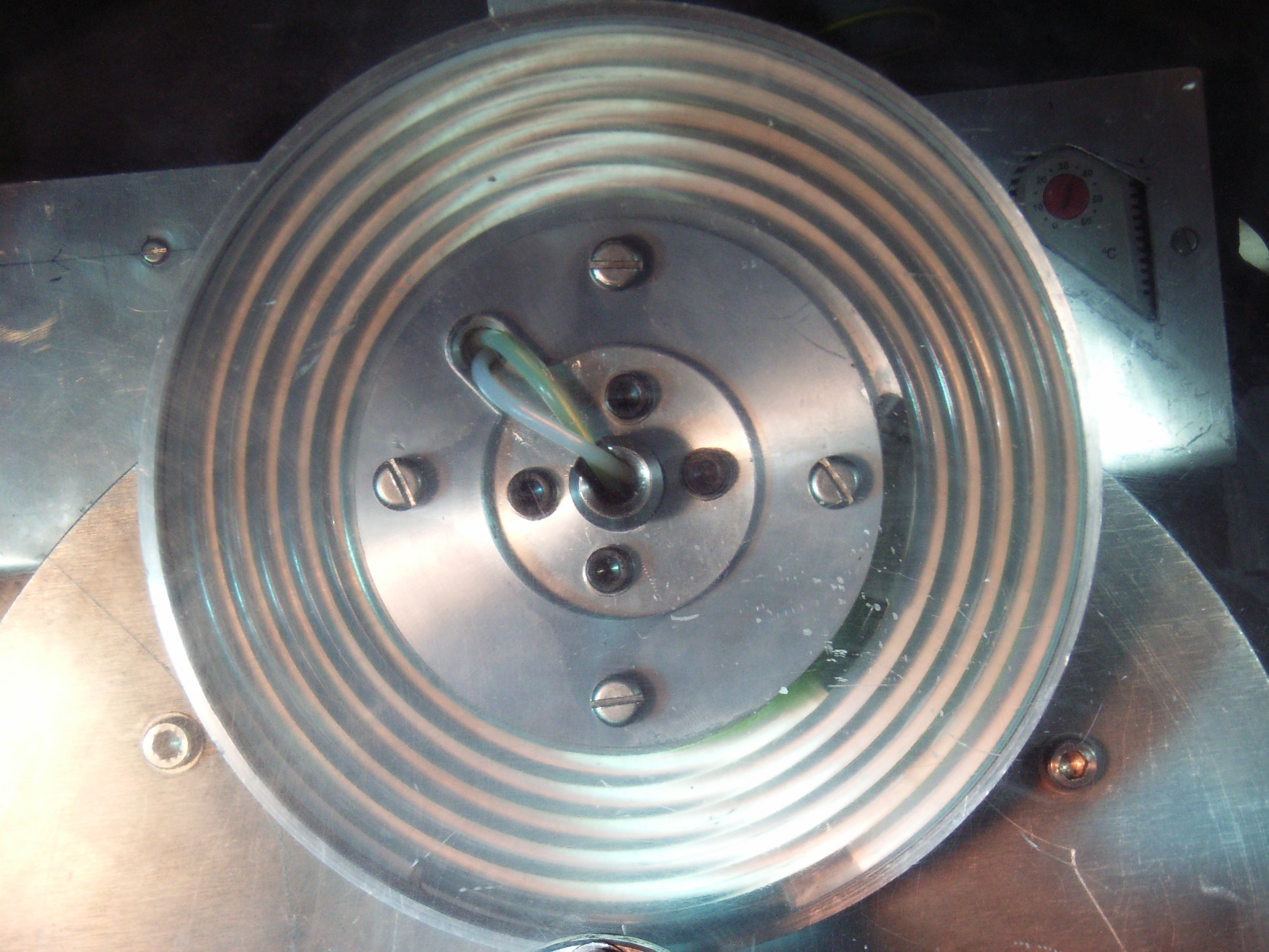 |
| S4-pt2-5  The dynamic image for the whole column  At time t=2 min  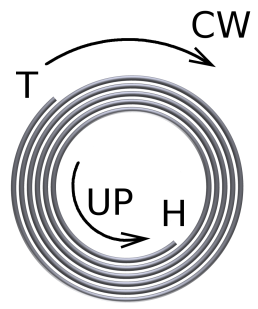 | 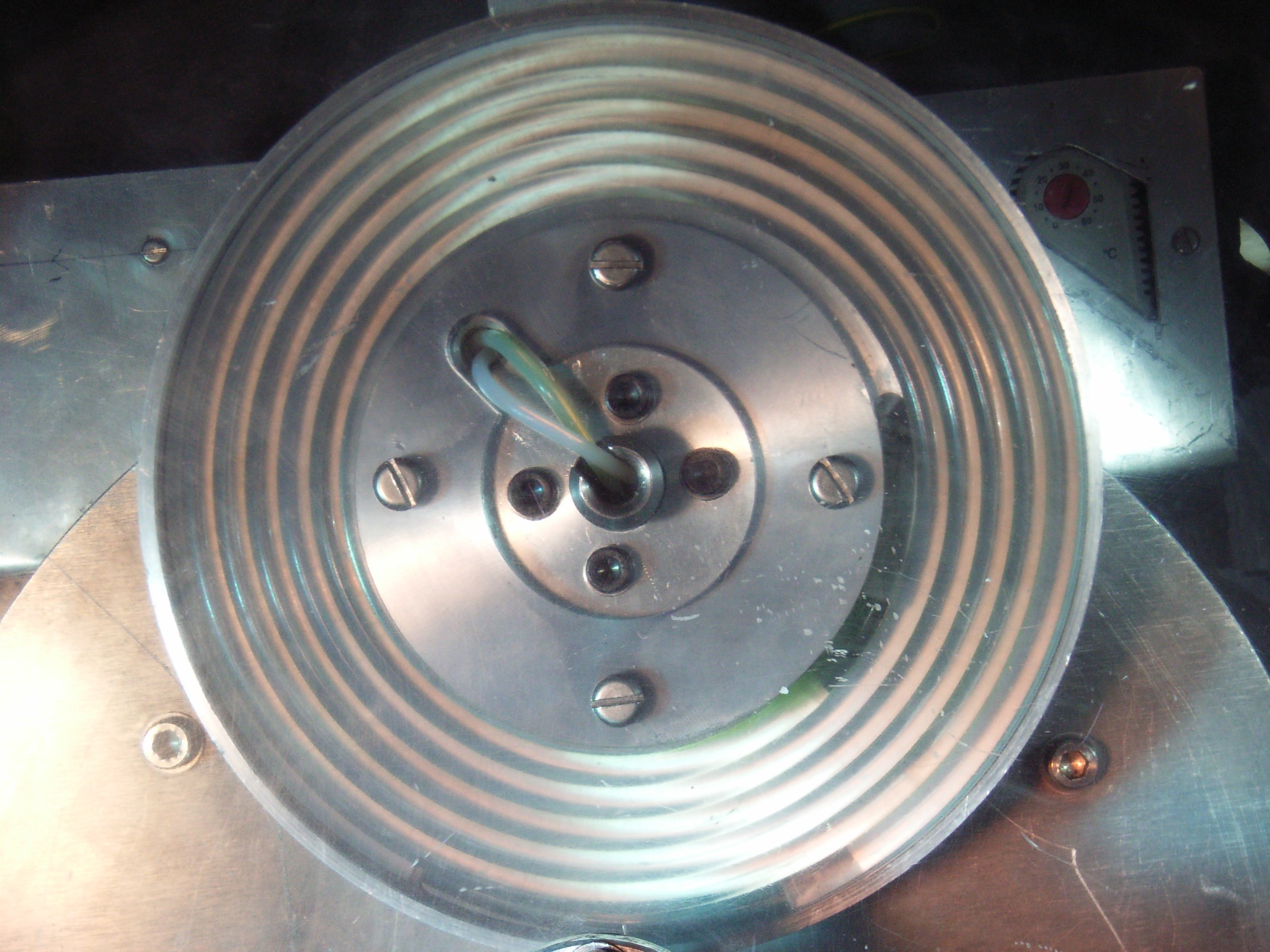 |
| S4-pt2-6  The dynamic image for the whole column  At time t=2.5 min  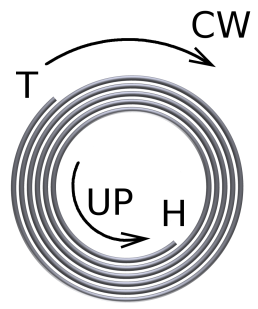 | 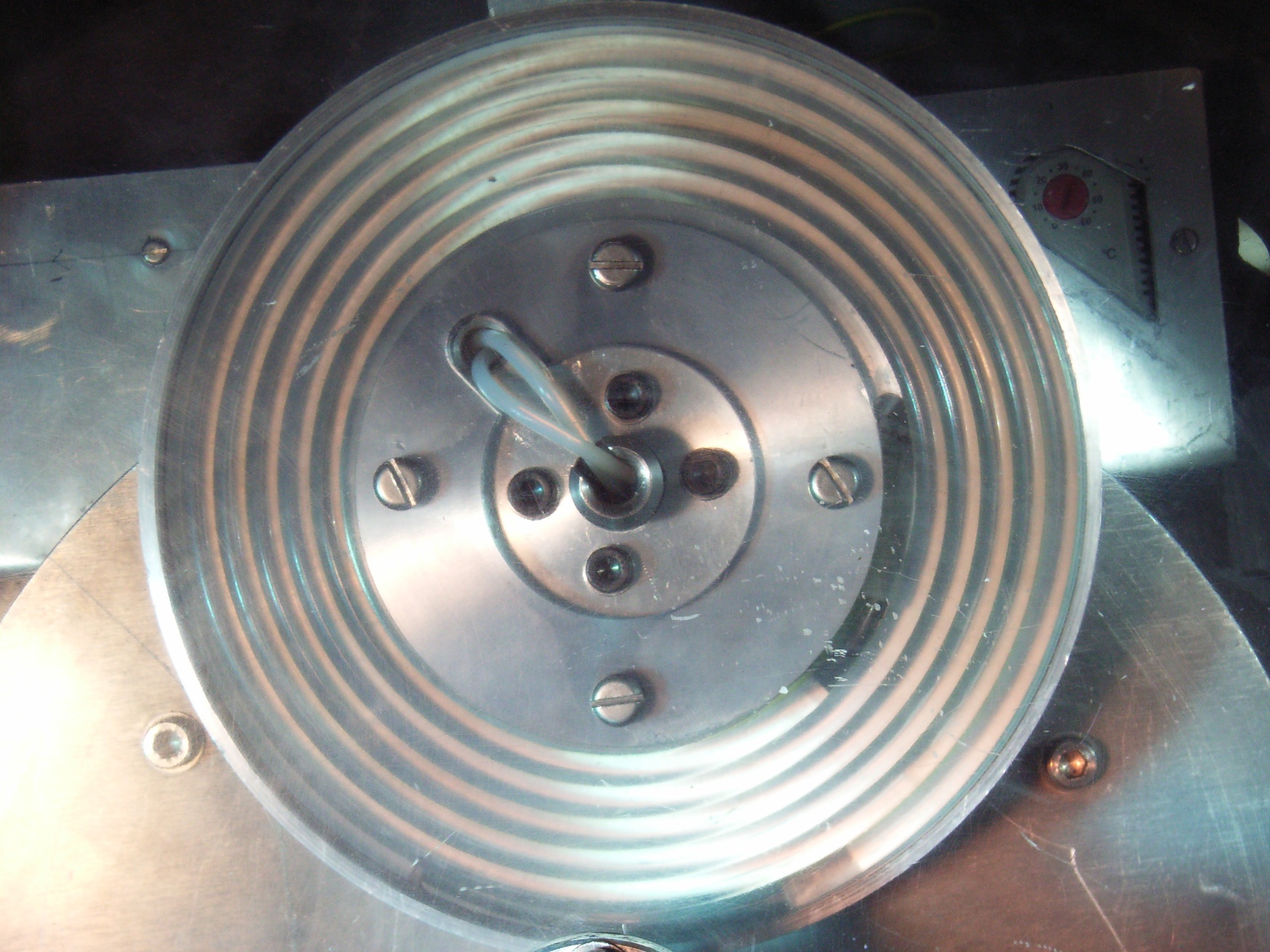 |
| S4-pt2-7  The dynamic image for the whole column  At time t=3 min  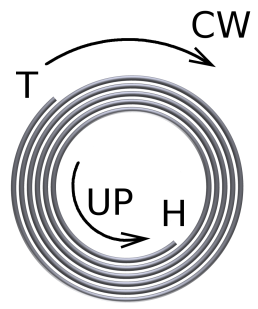 | 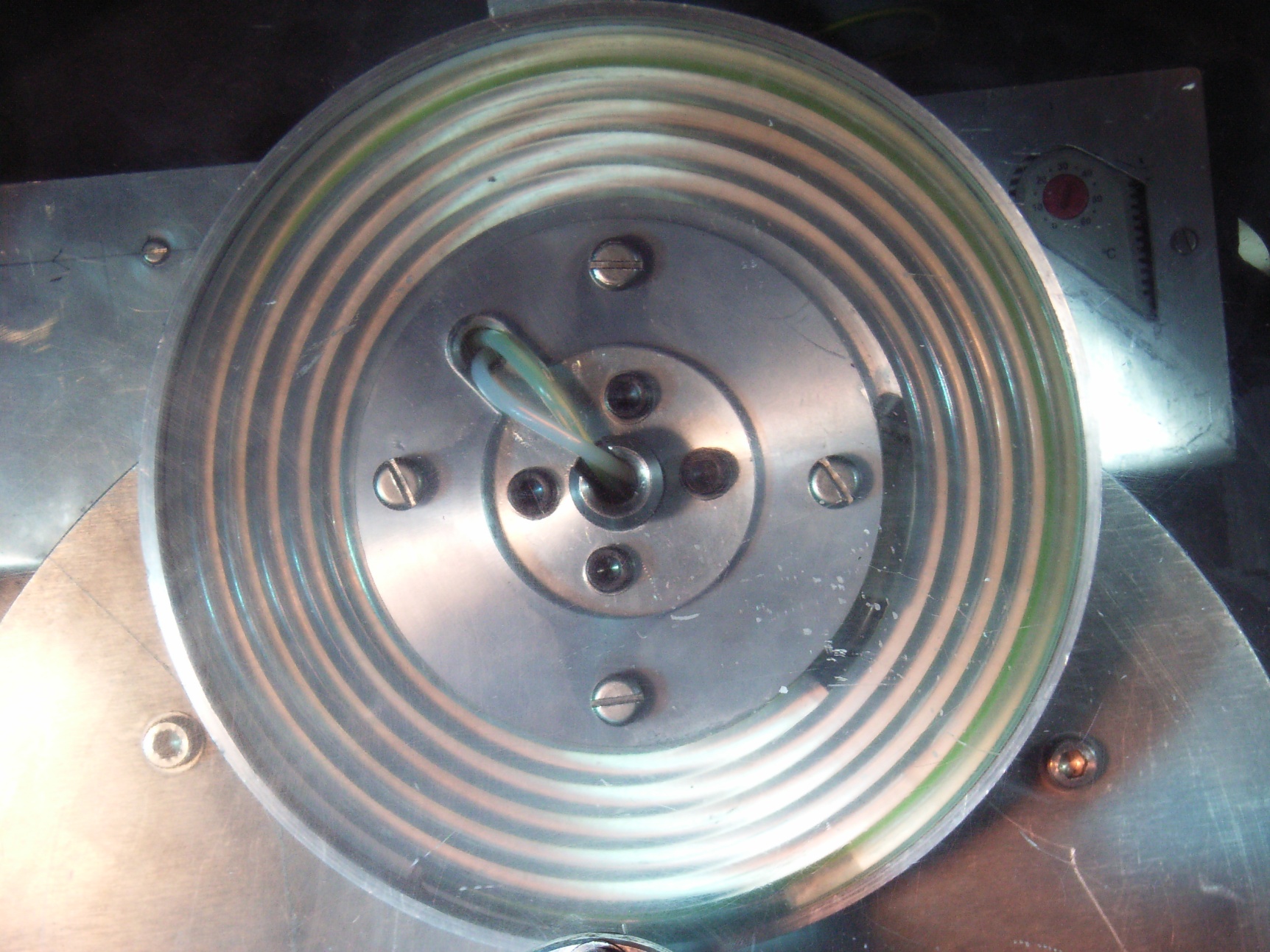 |
| S4-pt2-8  The dynamic image for the whole column  At time t=3.5 min  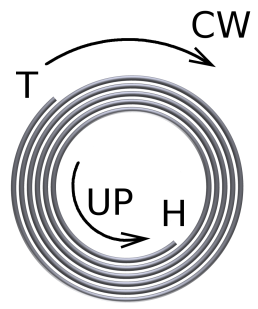 | 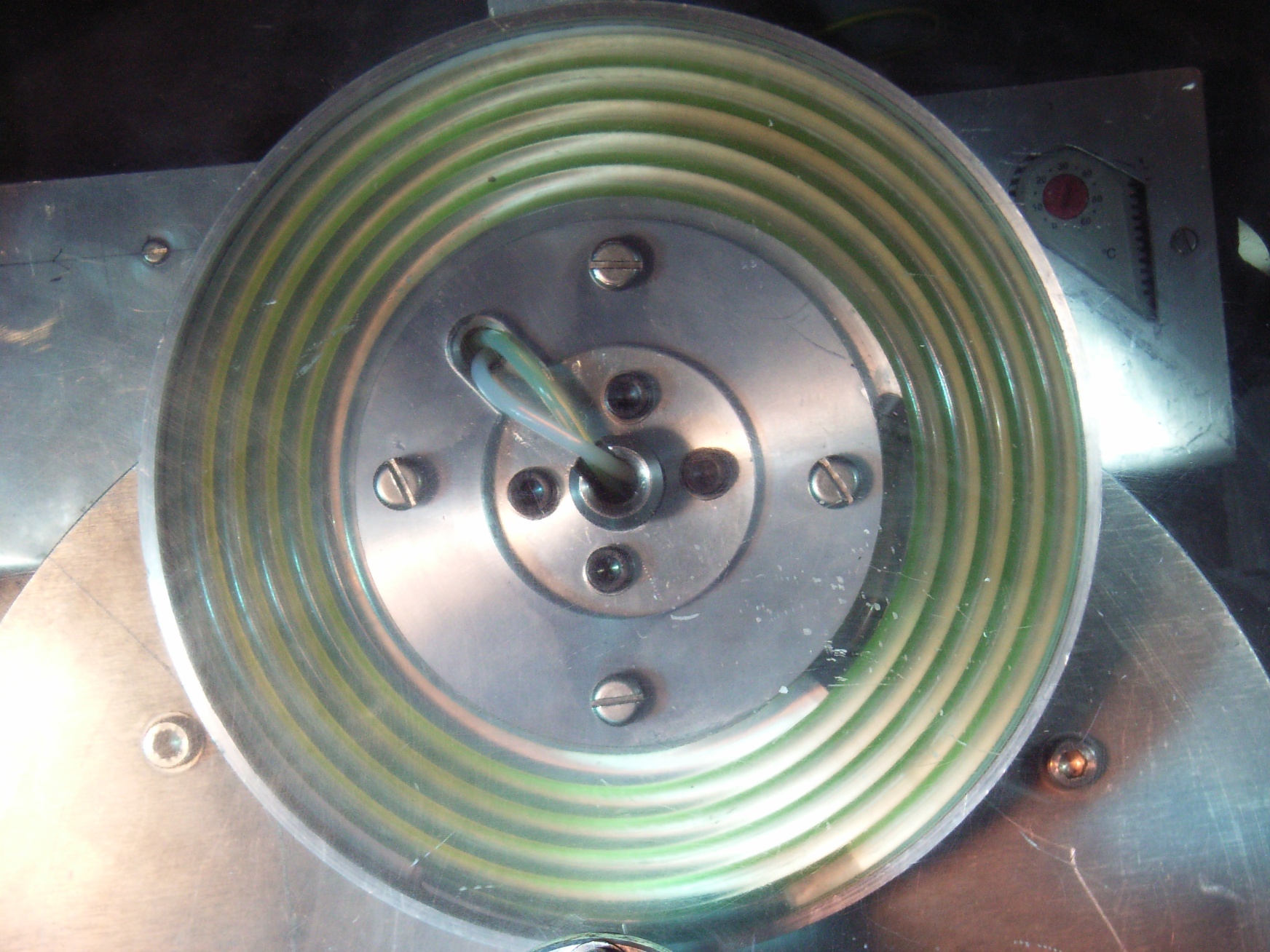 |
| S4-pt2-9  The dynamic image for the whole column  At time t=4.0 min  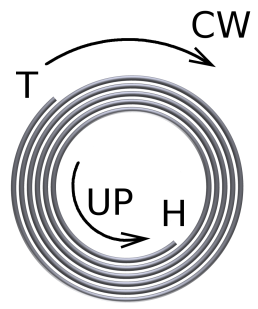 | 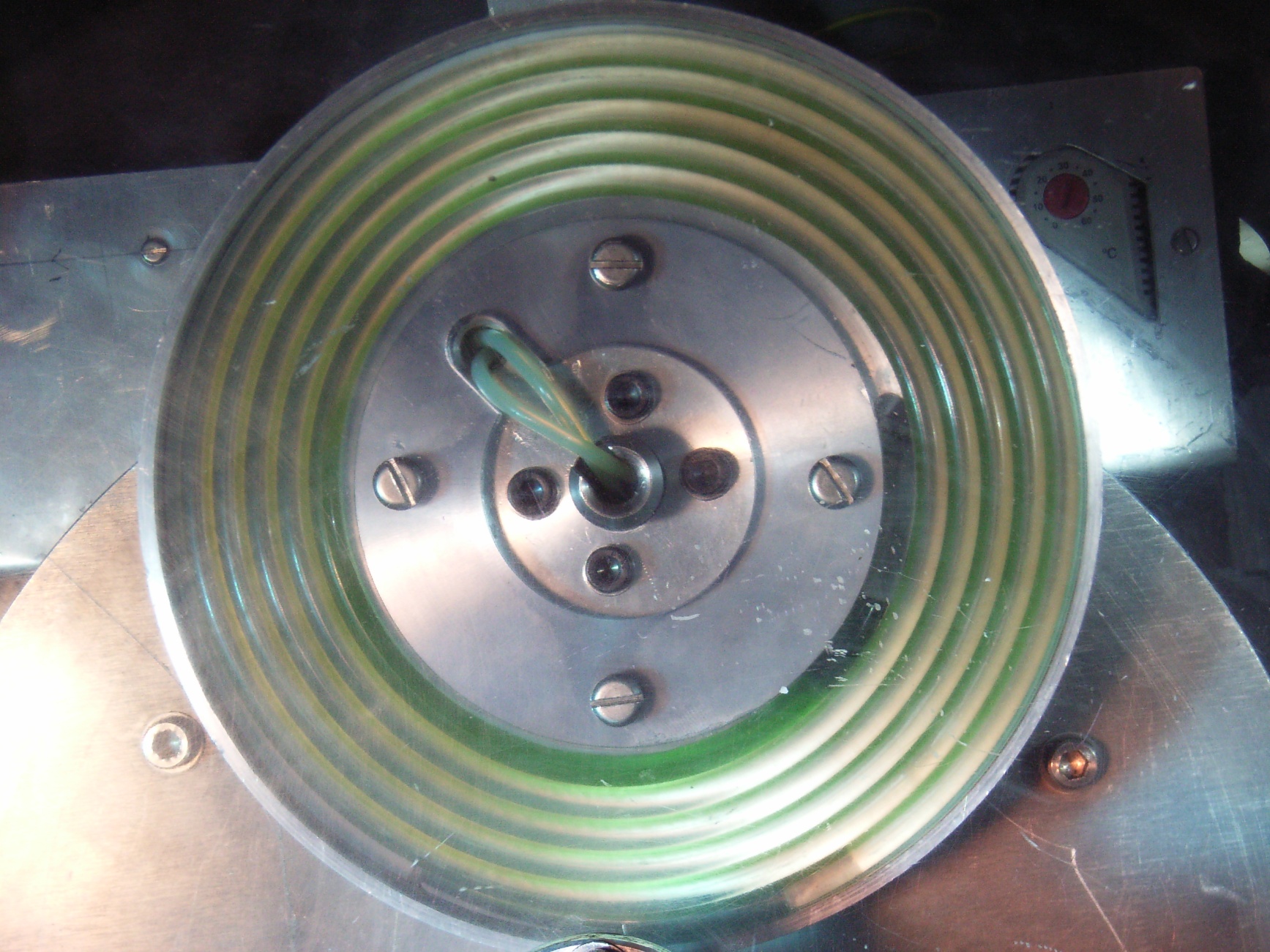 |
| S4-pt2-10  The dynamic image for the whole column  At time t=4.5 min  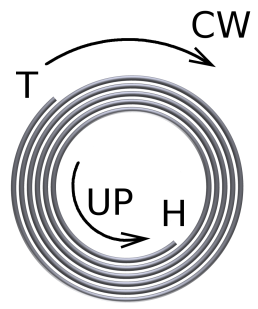 | 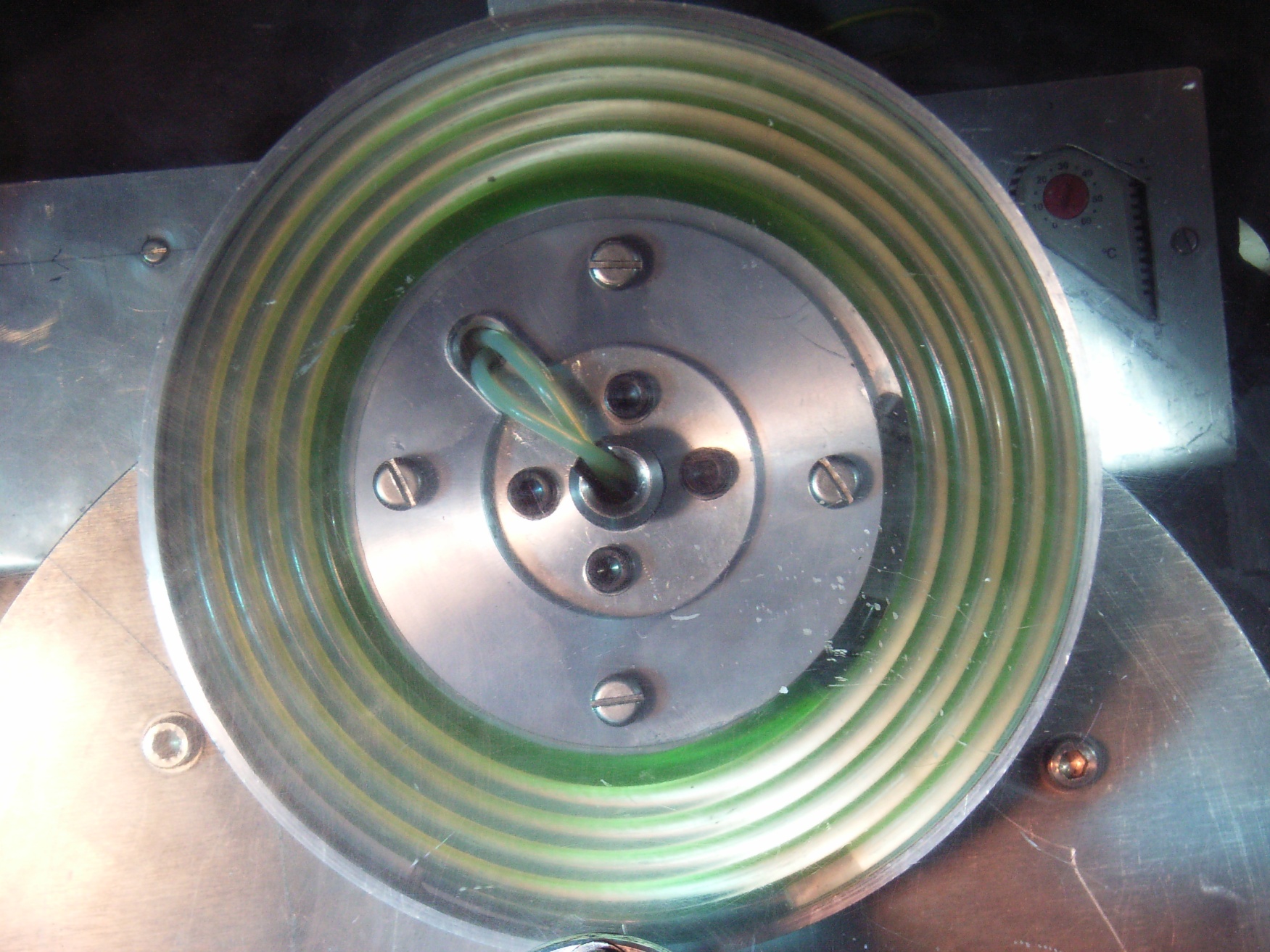 |
| S4-pt2-11  The dynamic image for the whole column  At time t=5.0 min  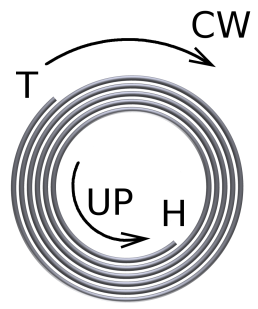 | 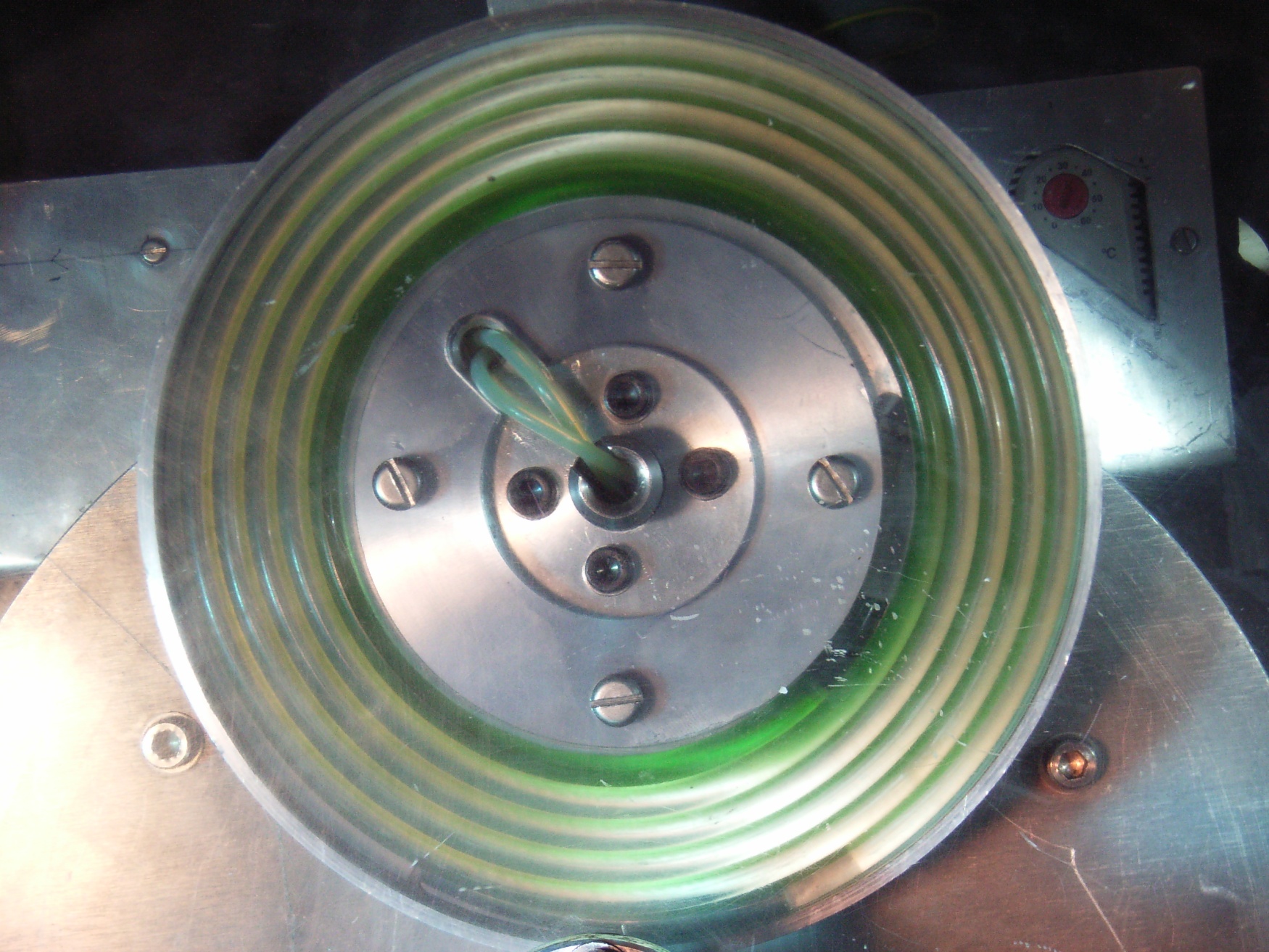 |
| S4-pt2-12  The dynamic image for the whole column  At time t=5.5 min  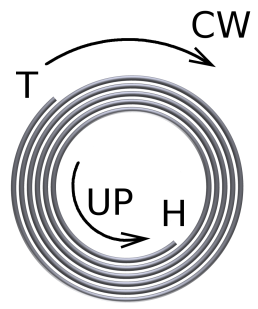 | 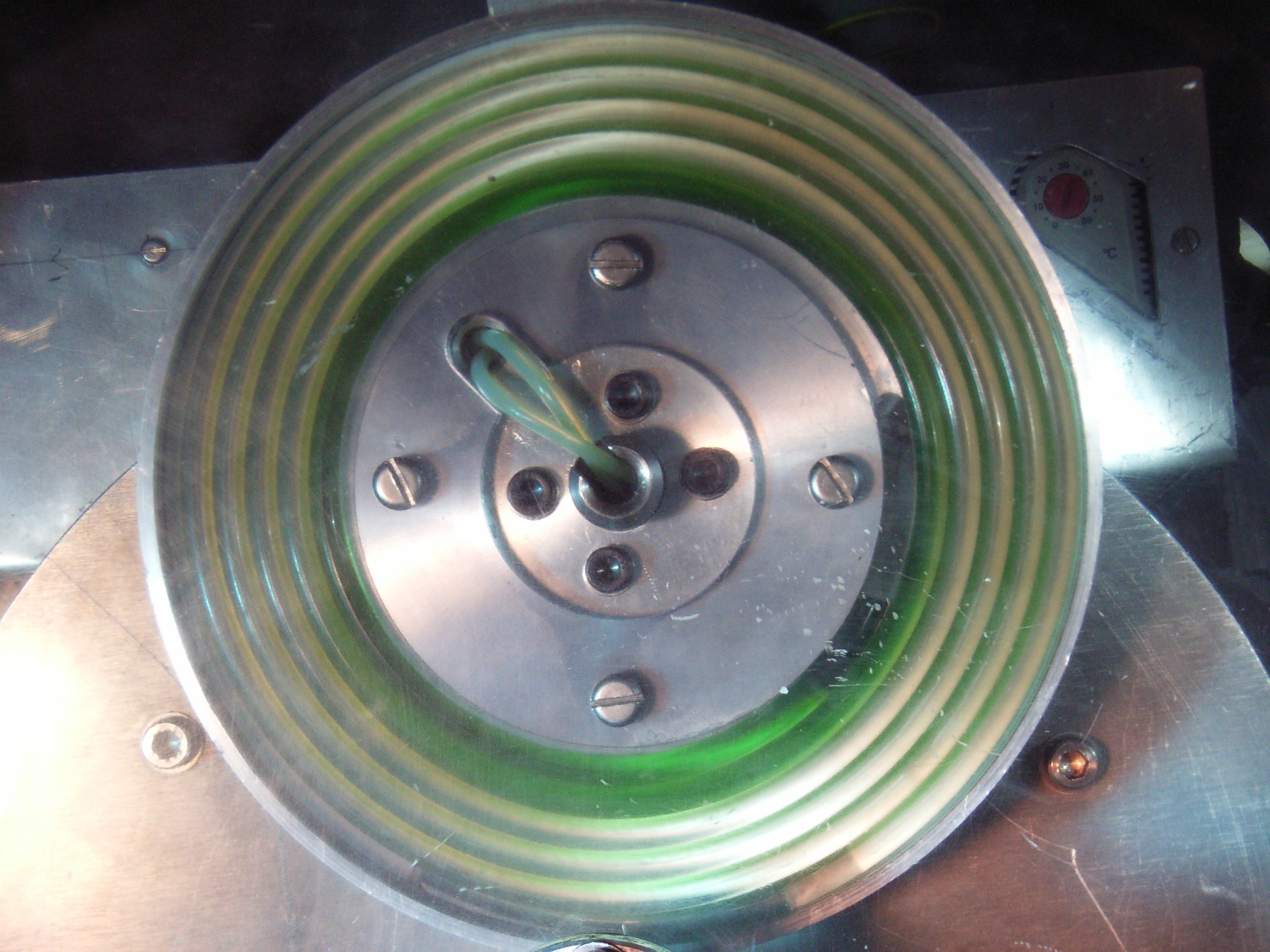 |
| S4-pt2-13  The dynamic image for the whole column  At time t=6.0 min  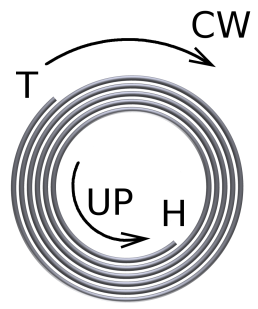 | 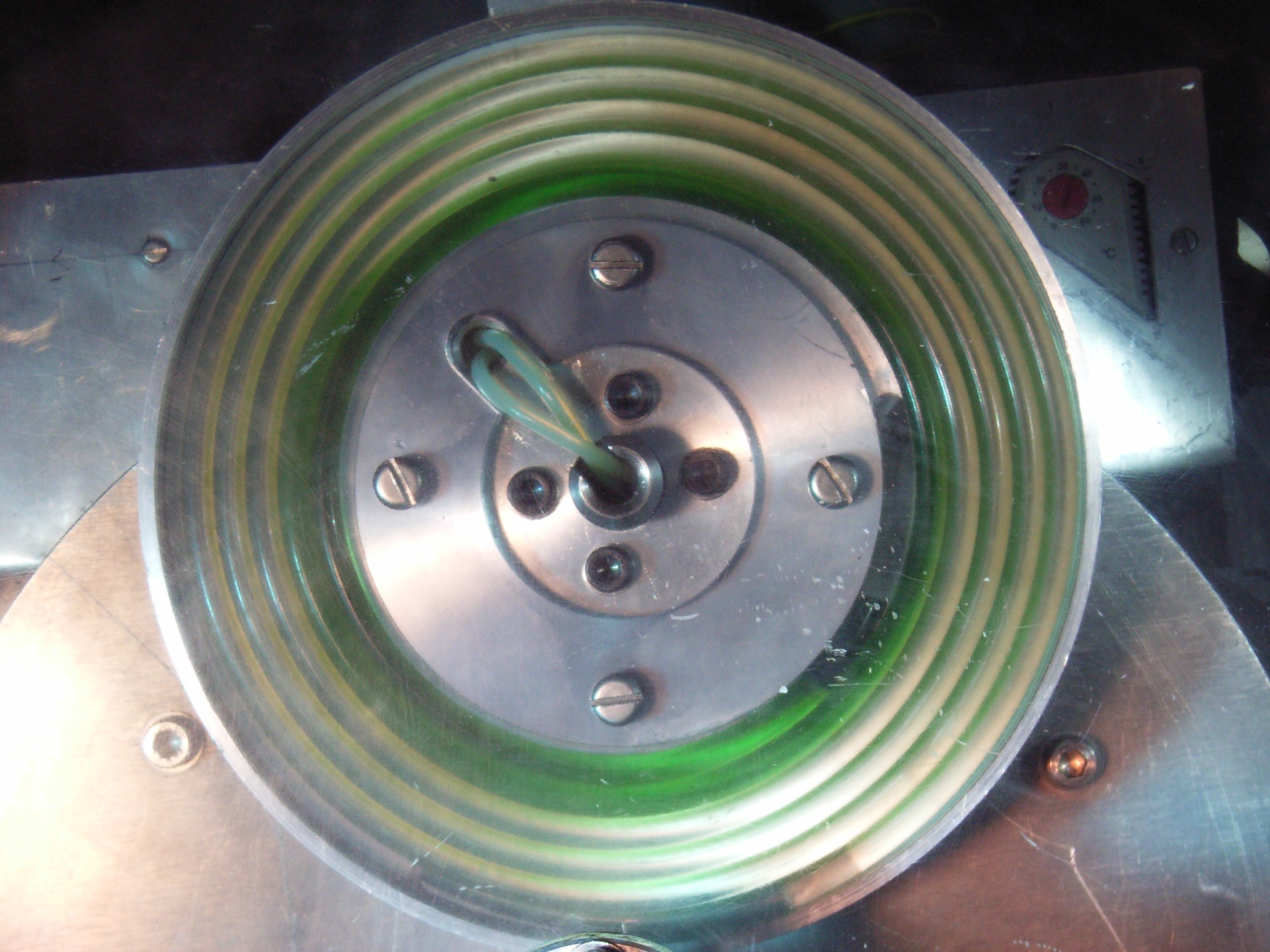 |
| S4-pt2-14  The dynamic image for the whole column  At time t=6.5 min  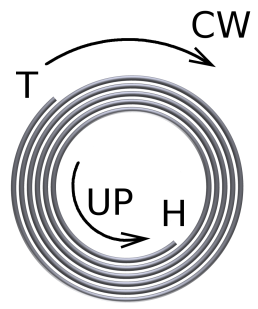 | 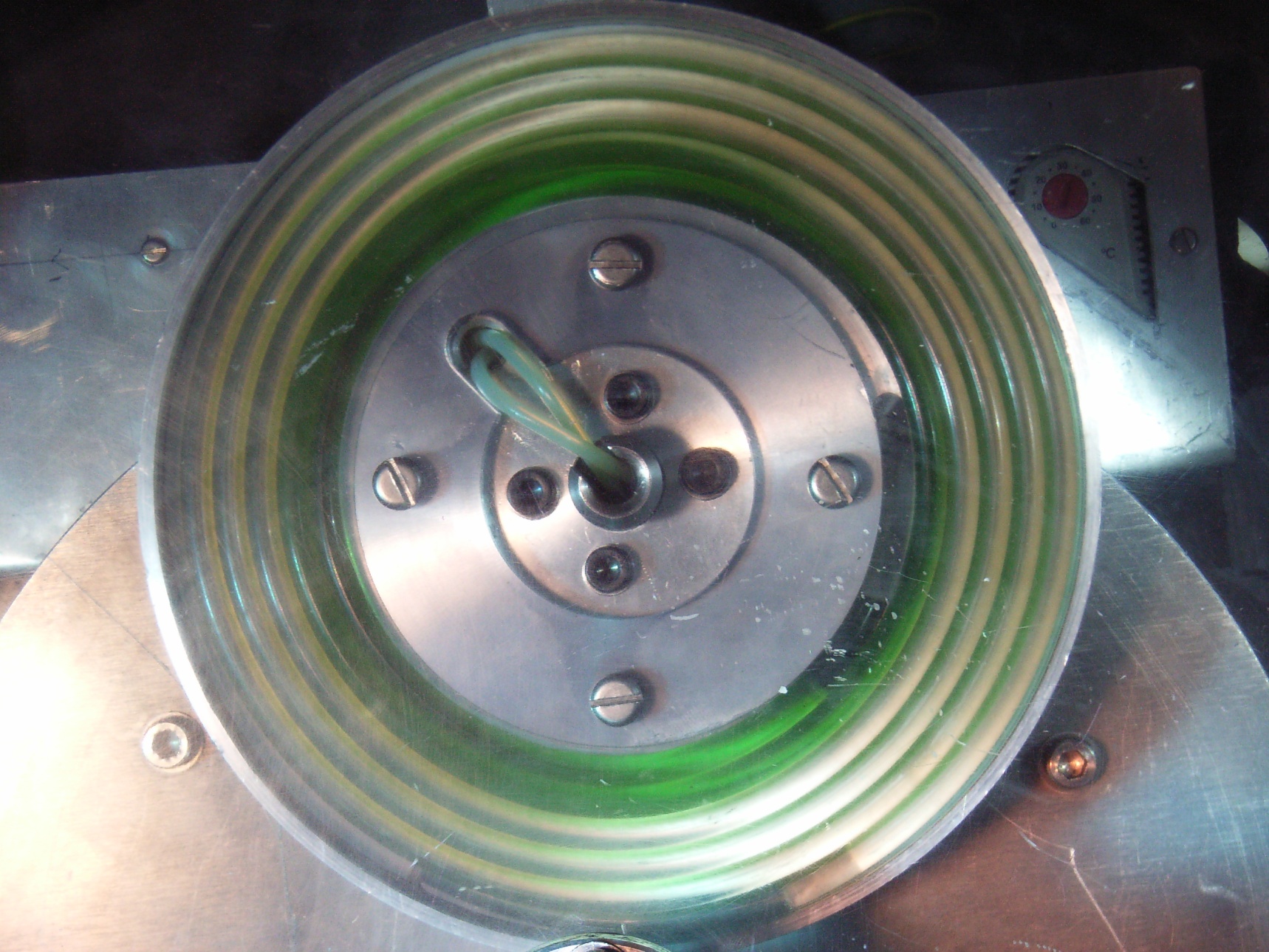 |
| S4-pt2-15  The dynamic image for the whole column  At time t=7.0 min  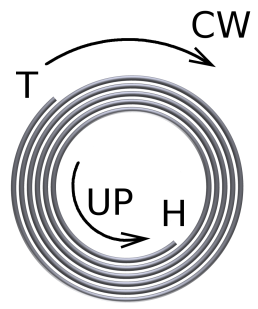 | 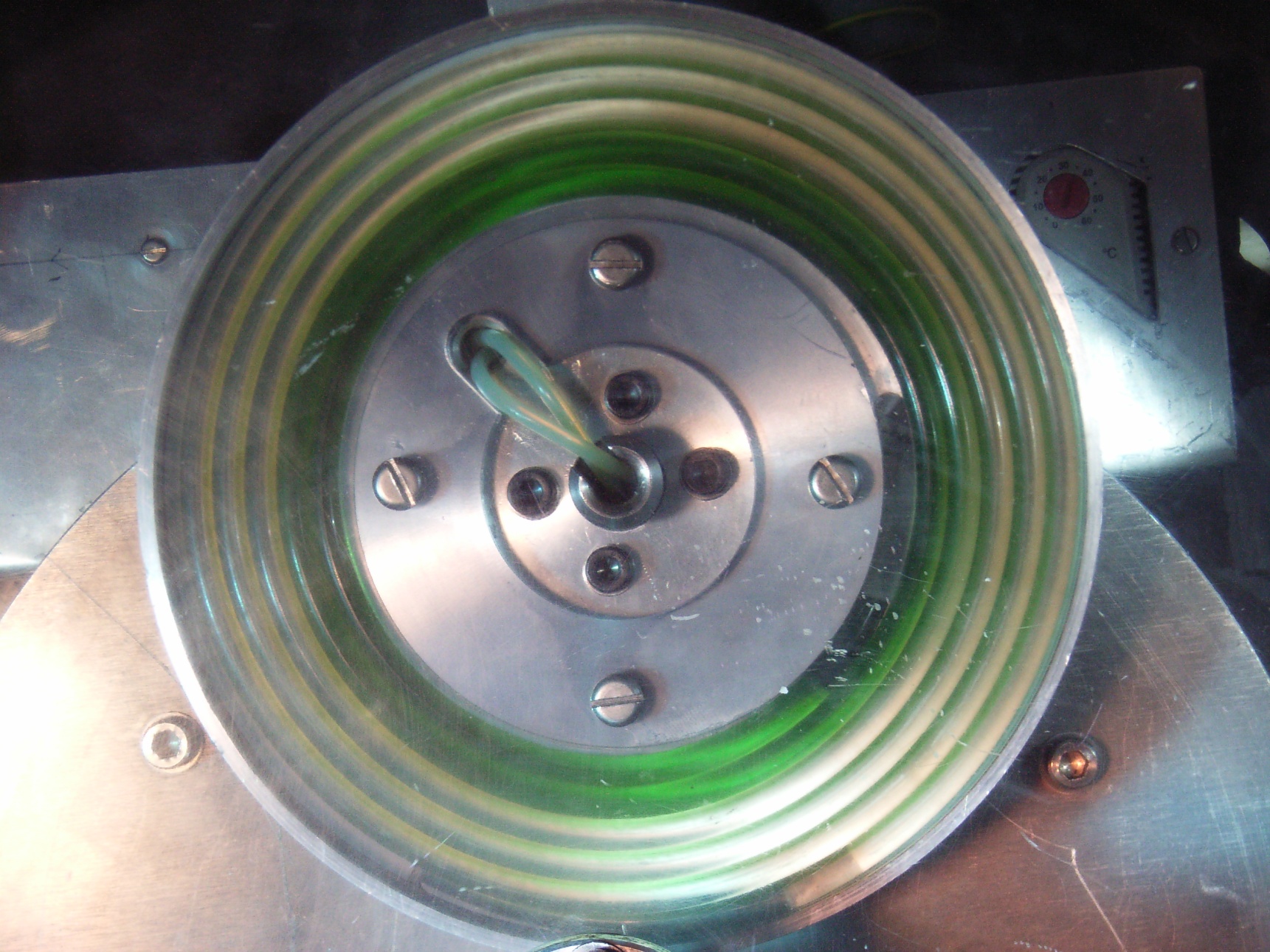 |
| S4-pt2-16  The dynamic image for the whole column  At time t=7.5 min  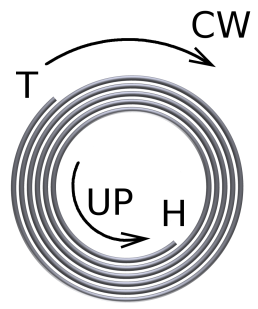 | 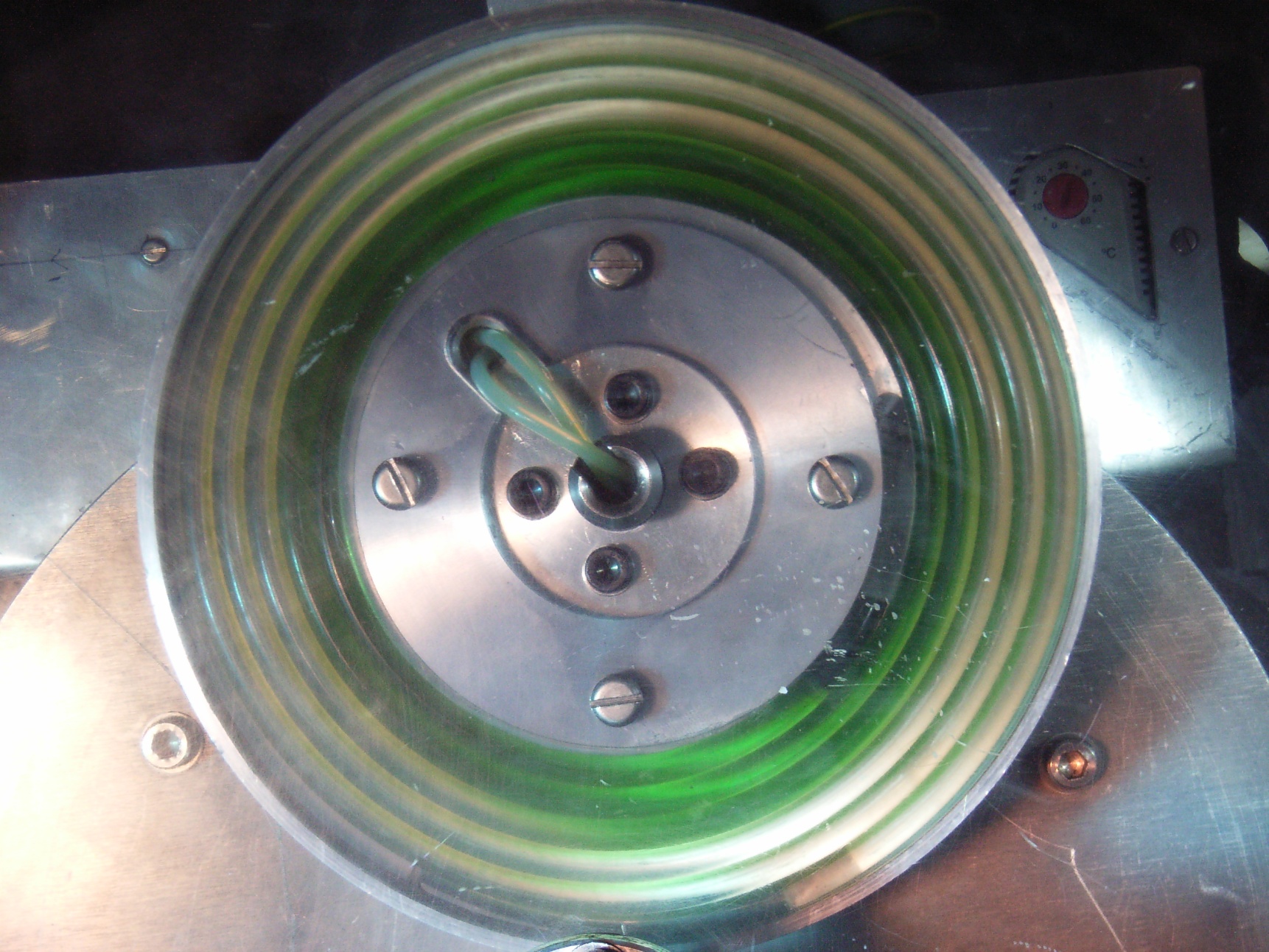 |
| S4-pt2-17  The dynamic image for the whole column  At time t=8.0 min  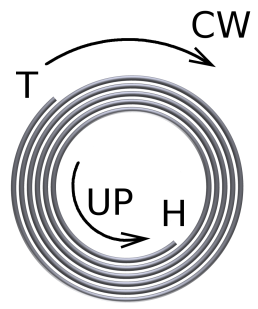 | 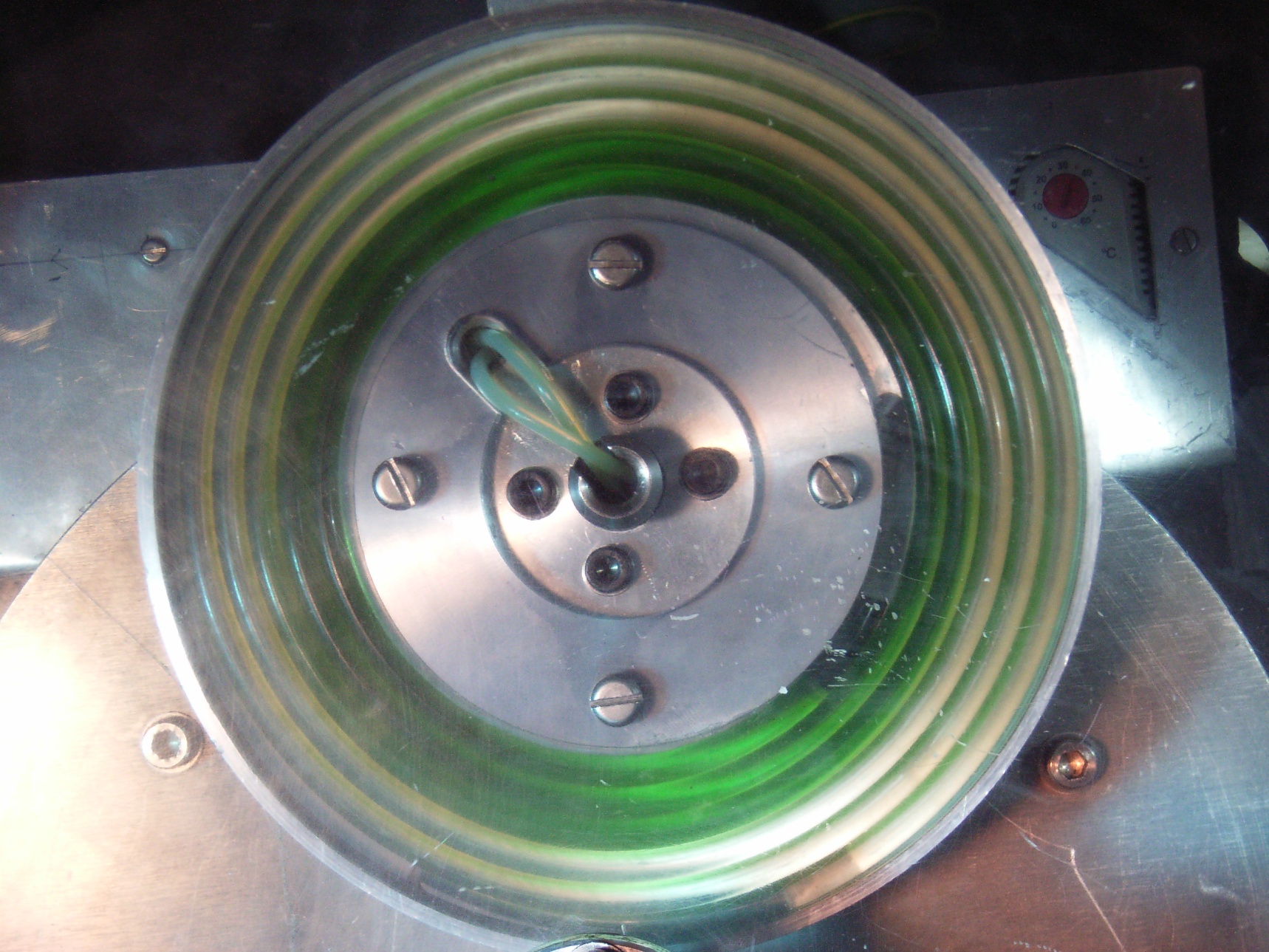 |
| S4-pt2-18  The dynamic image for the whole column  At time t=8.5 min  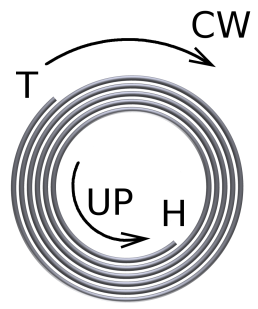 | 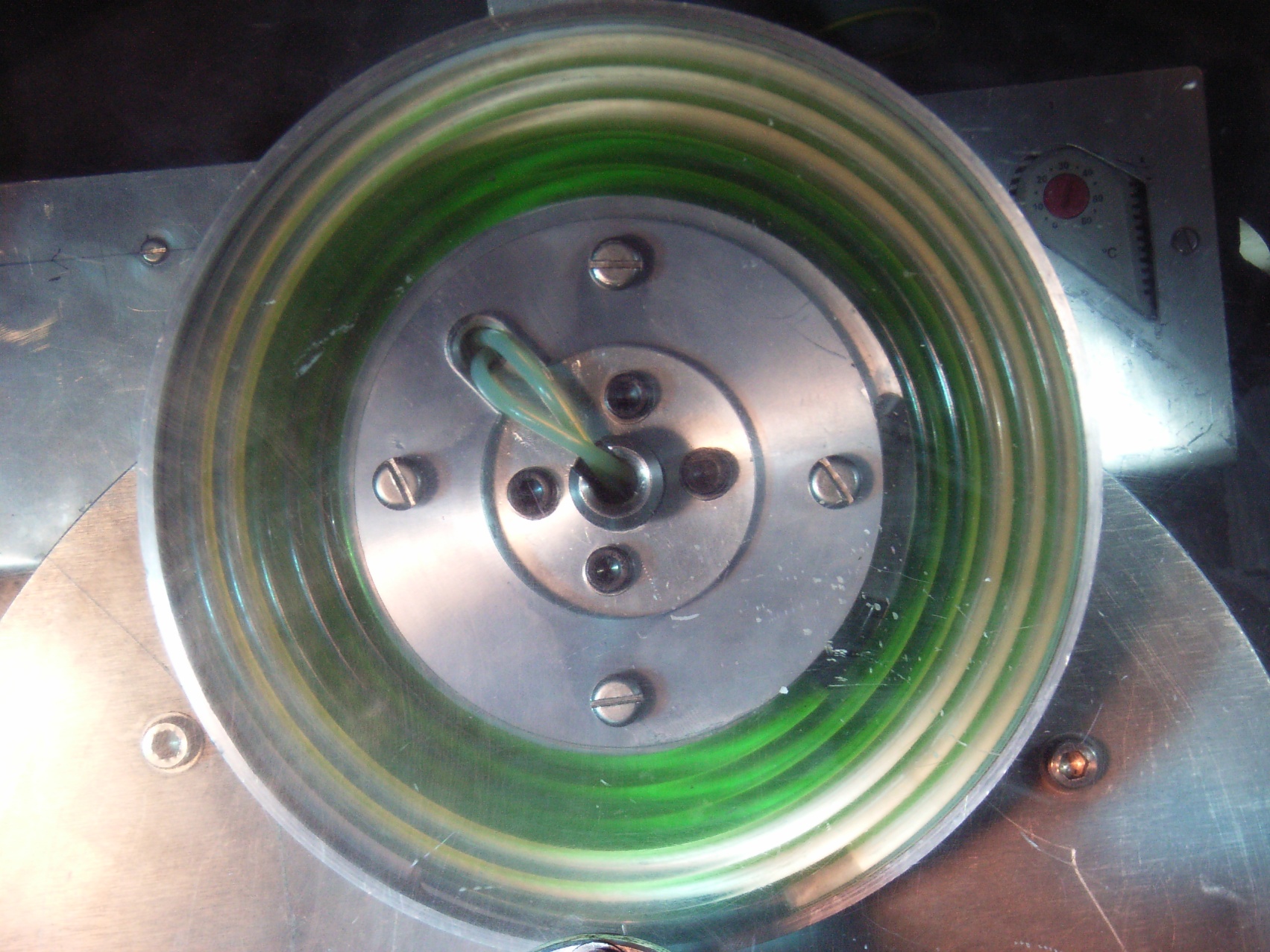 |
| S4-pt2-19  The dynamic image for the whole column  At time t=9.0 min  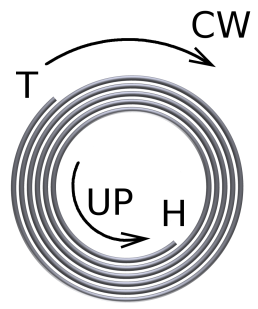 | 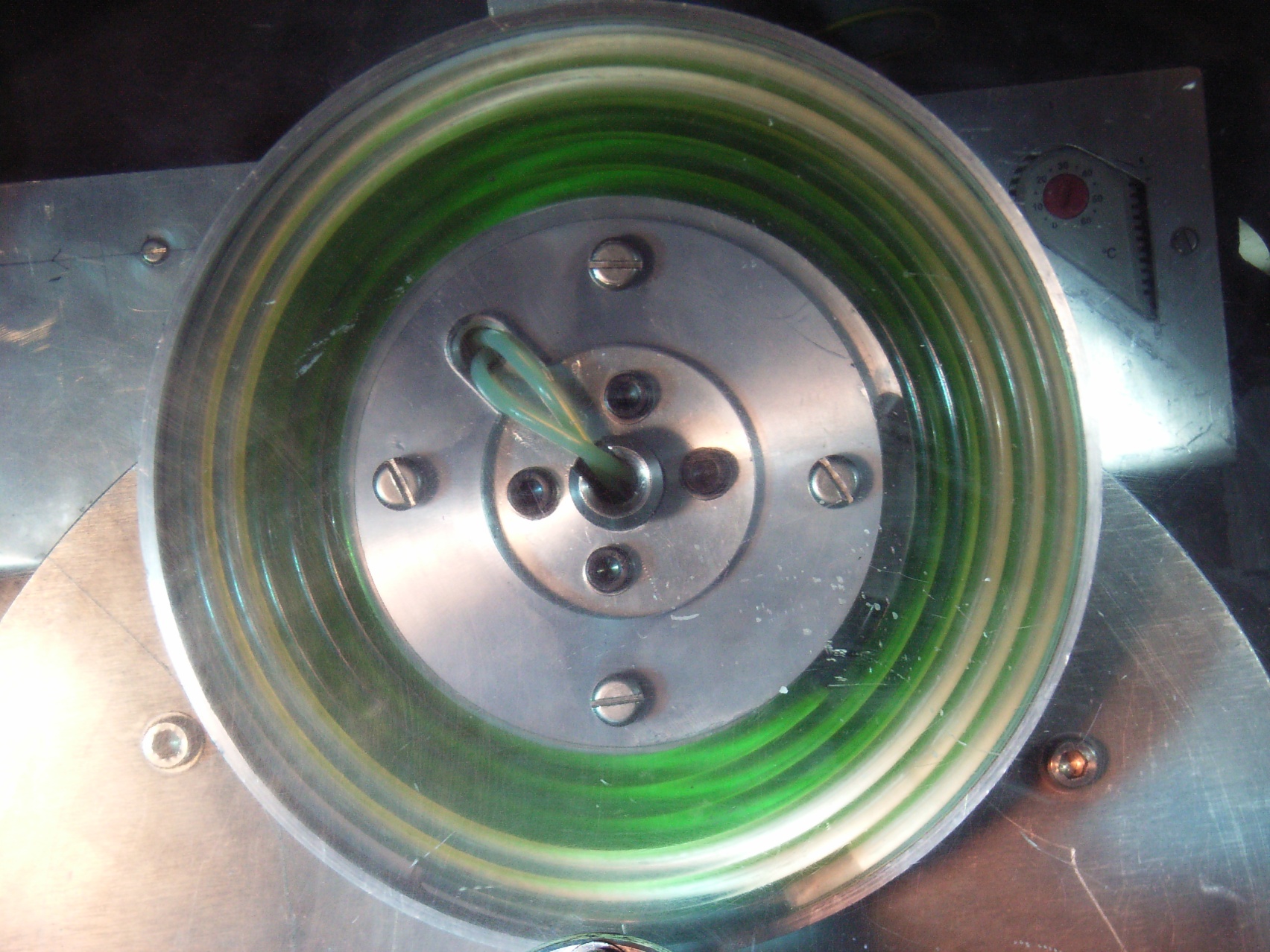 |
| S4-pt2-20  The dynamic image for the whole column  At time t=9.5 min  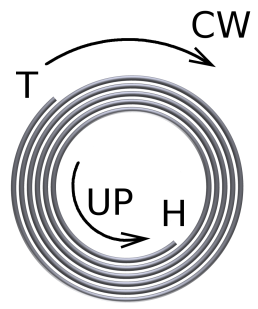 | 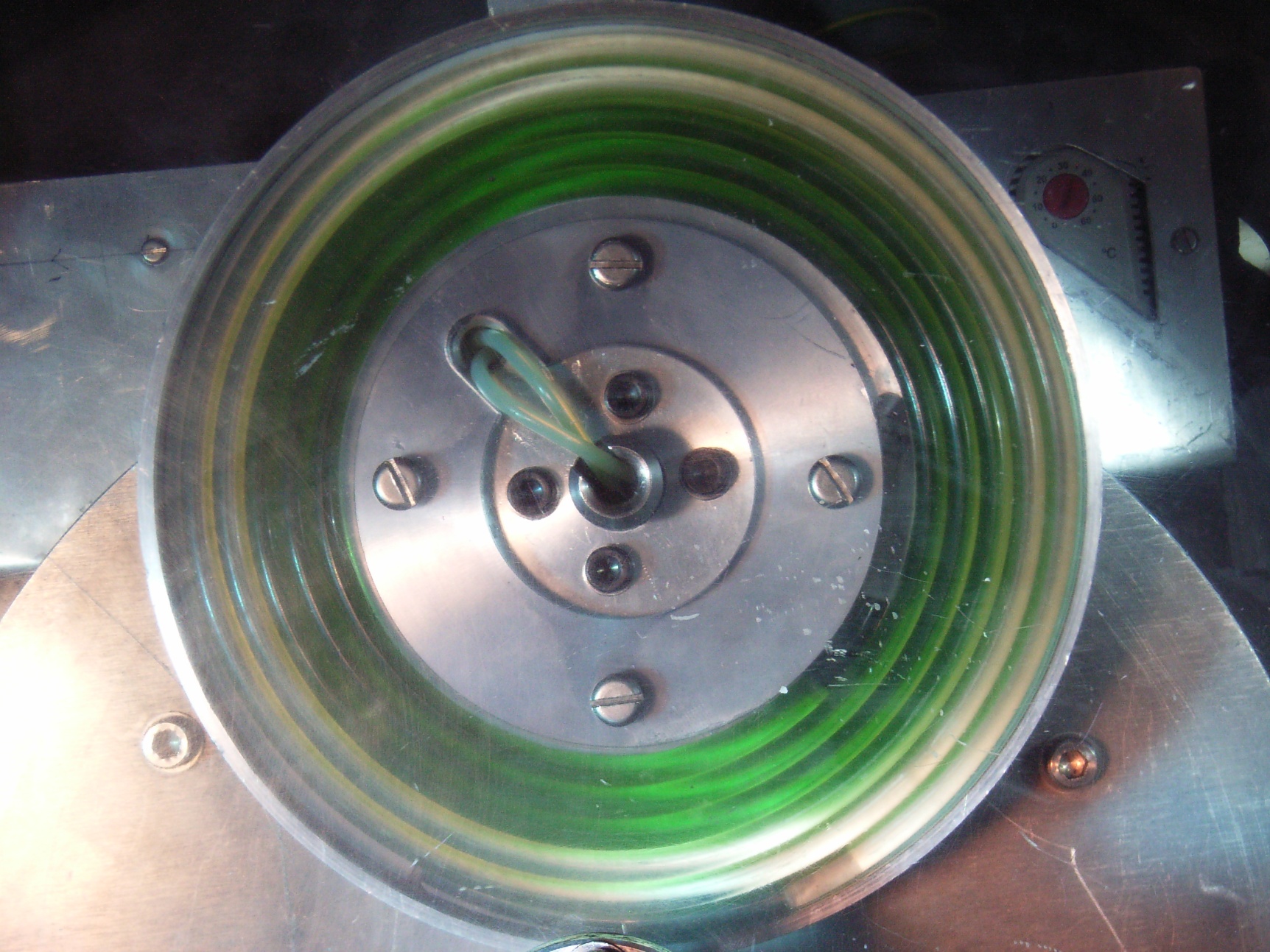 |
| S4-pt2-21  The dynamic image for the whole column  At time t=10.0 min  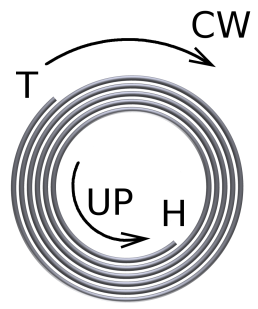 | 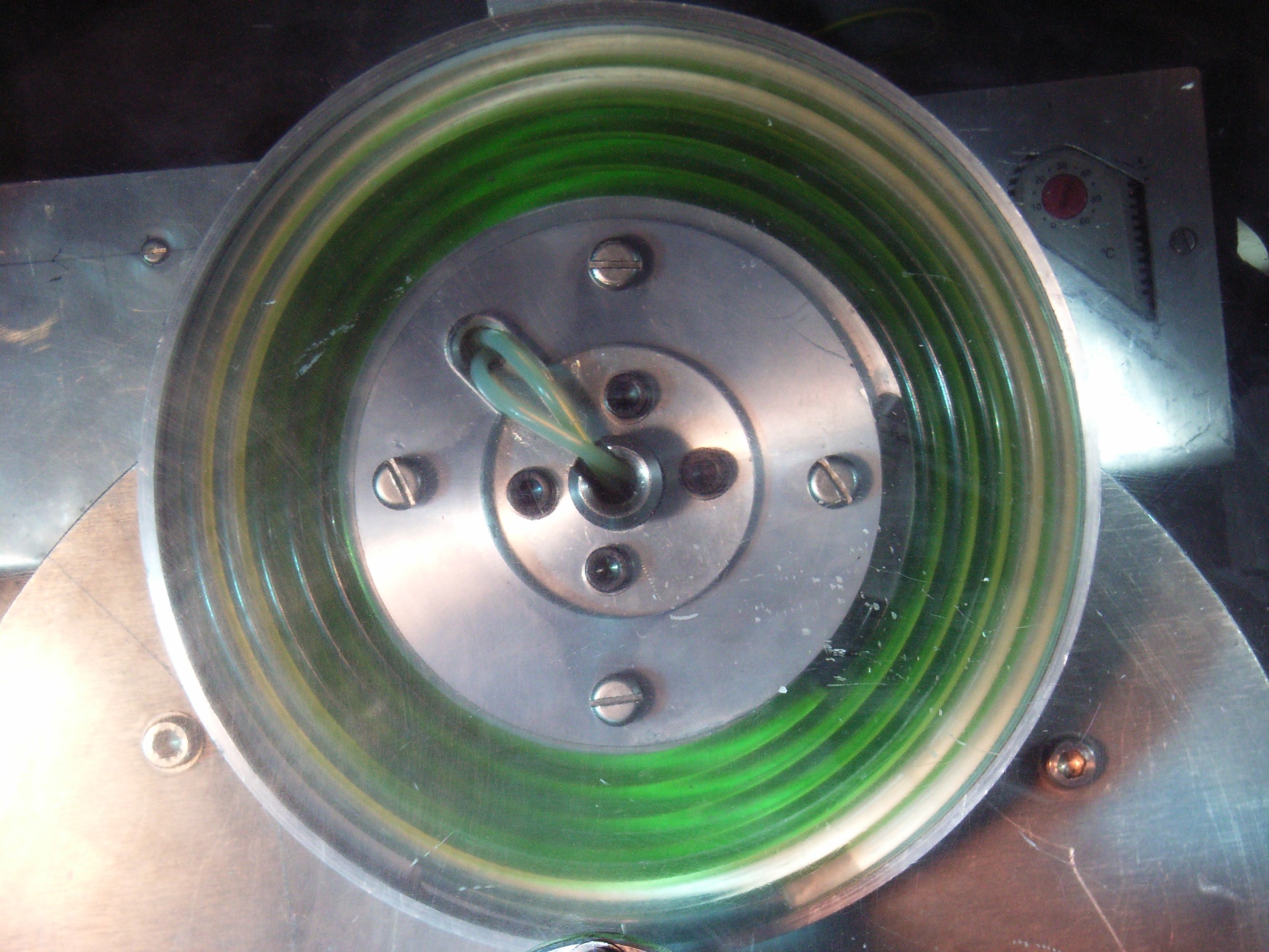 |
| S4-pt2-22  The dynamic image for the whole column  At time t=10.5 min  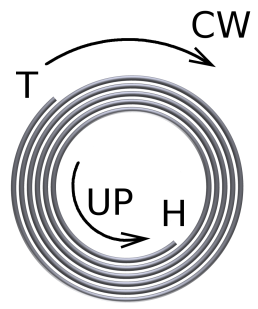 | 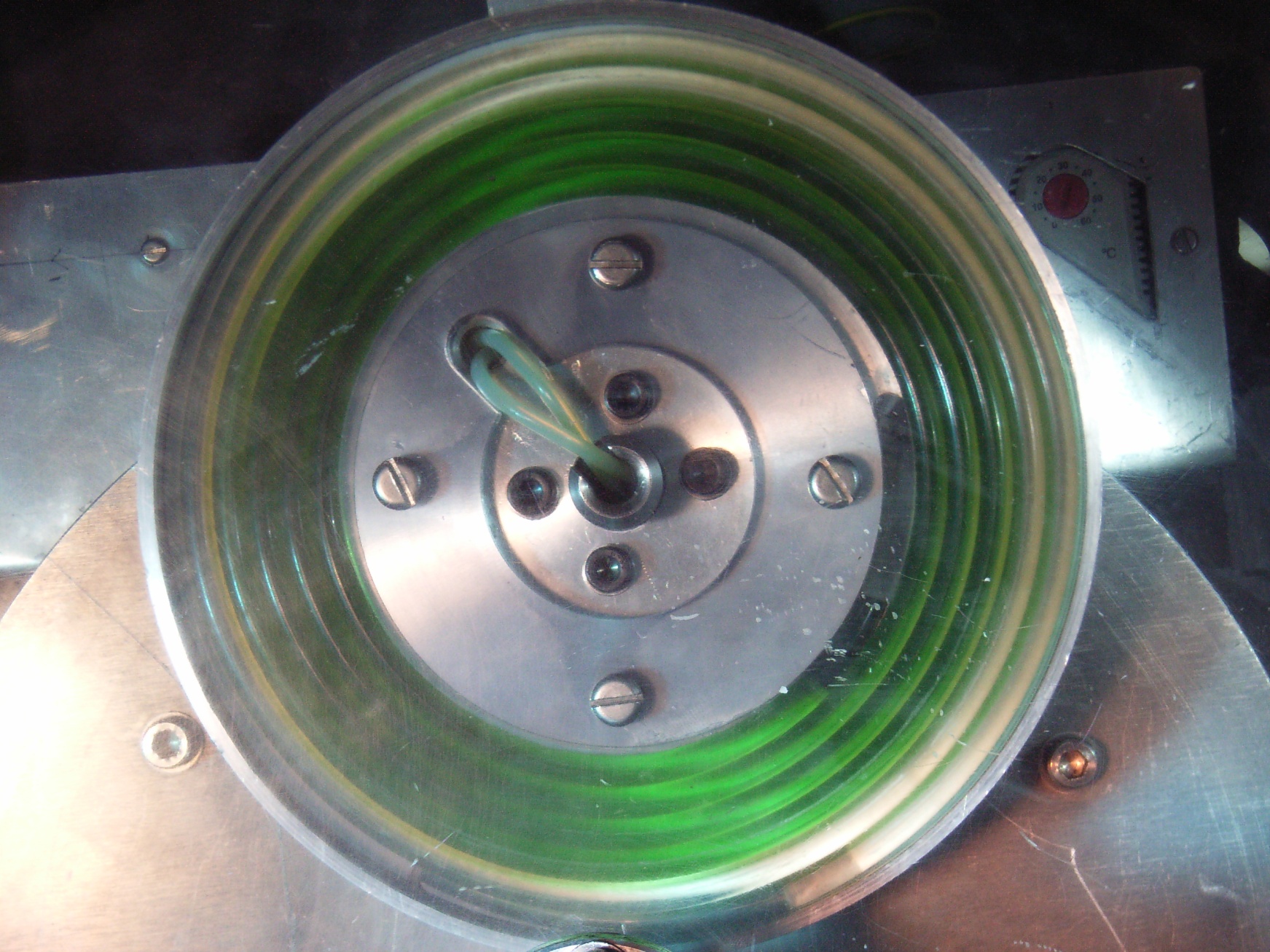 |
| S4-pt2-23  The dynamic image for the whole column  At time t=11.0 min  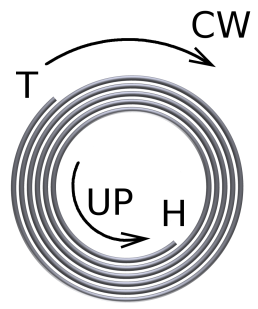 | 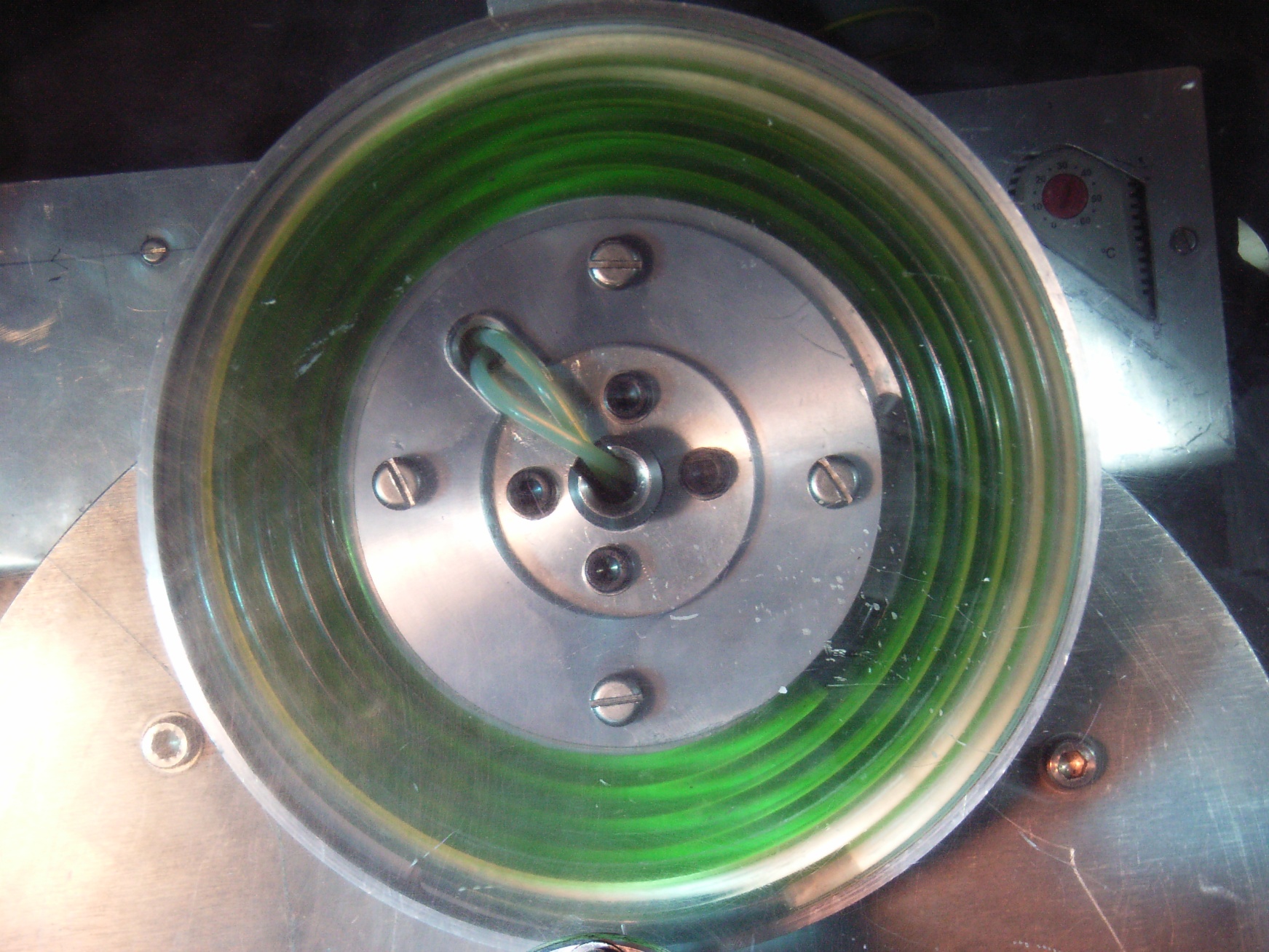 |
| S4-pt2-24  The dynamic image for the whole column  At time t=11.5 min  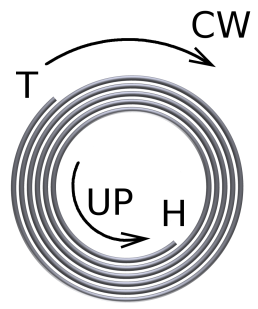 | 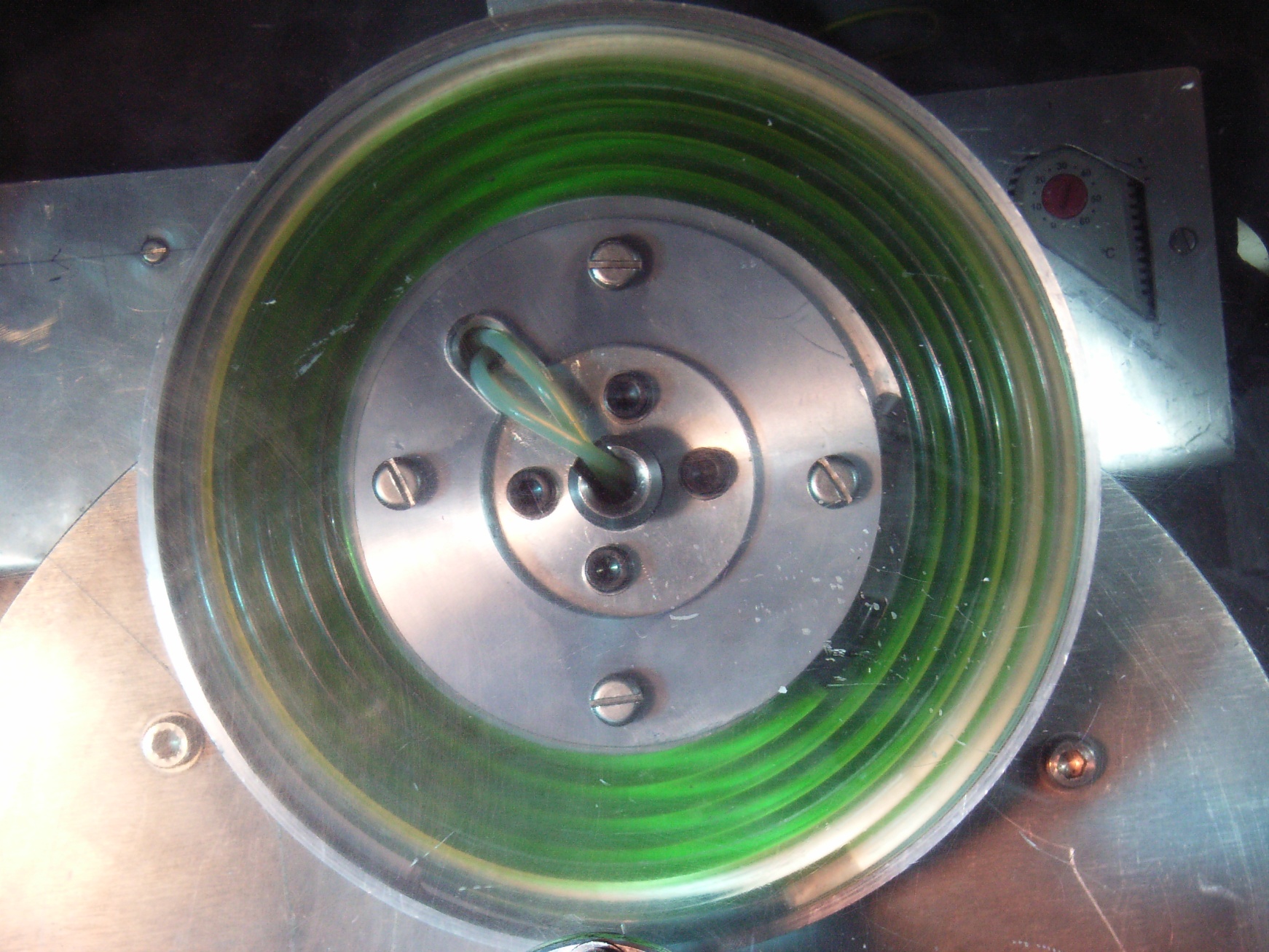 |
| S4-pt2-25  The dynamic image for the whole column  At time t=12.0 min  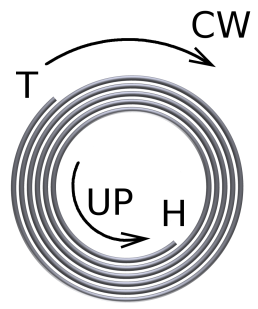 | 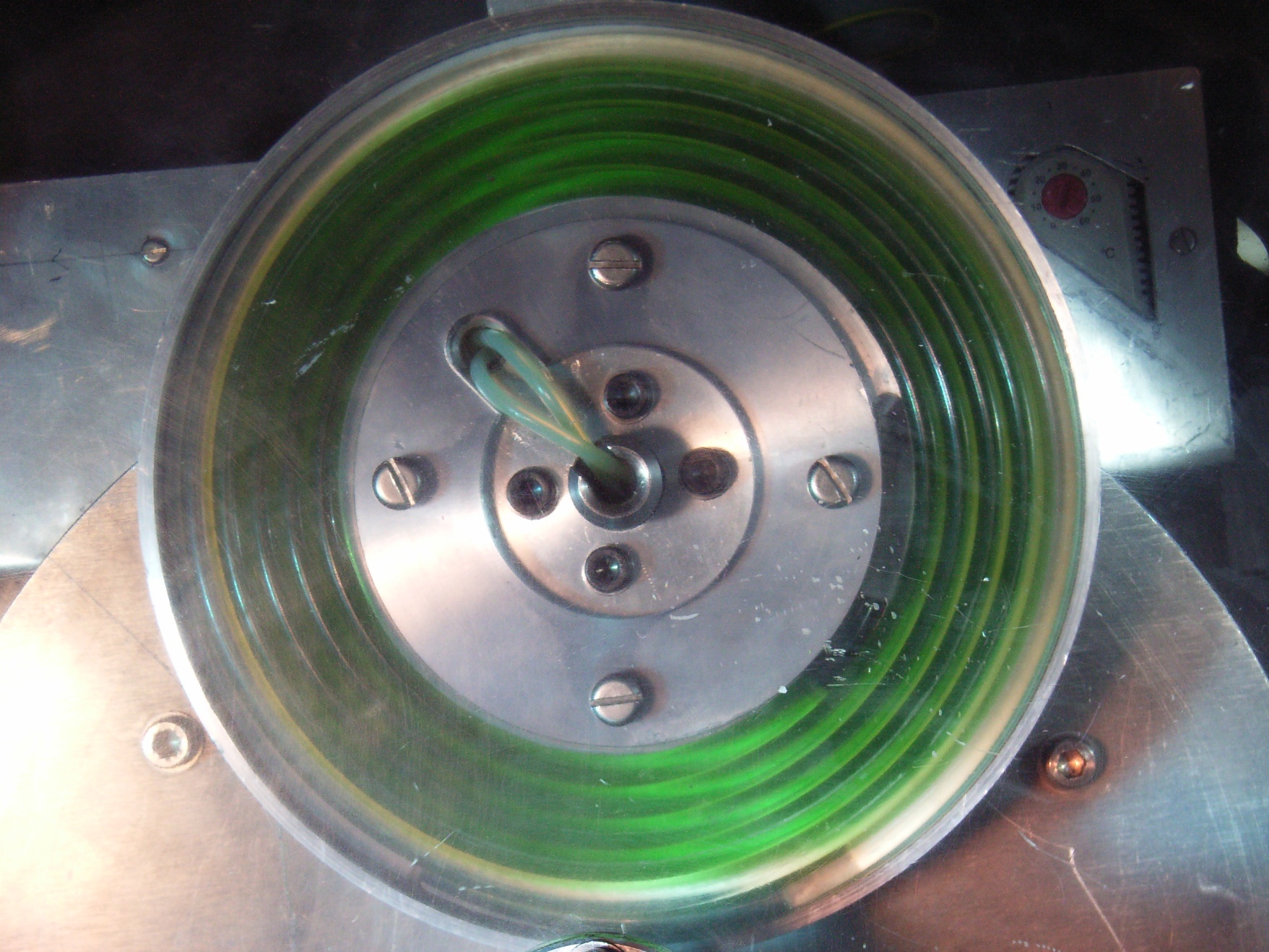 |
| S4-pt2-26  The dynamic image for the whole column  At time t=12.5 min  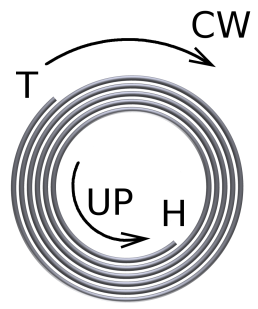 | 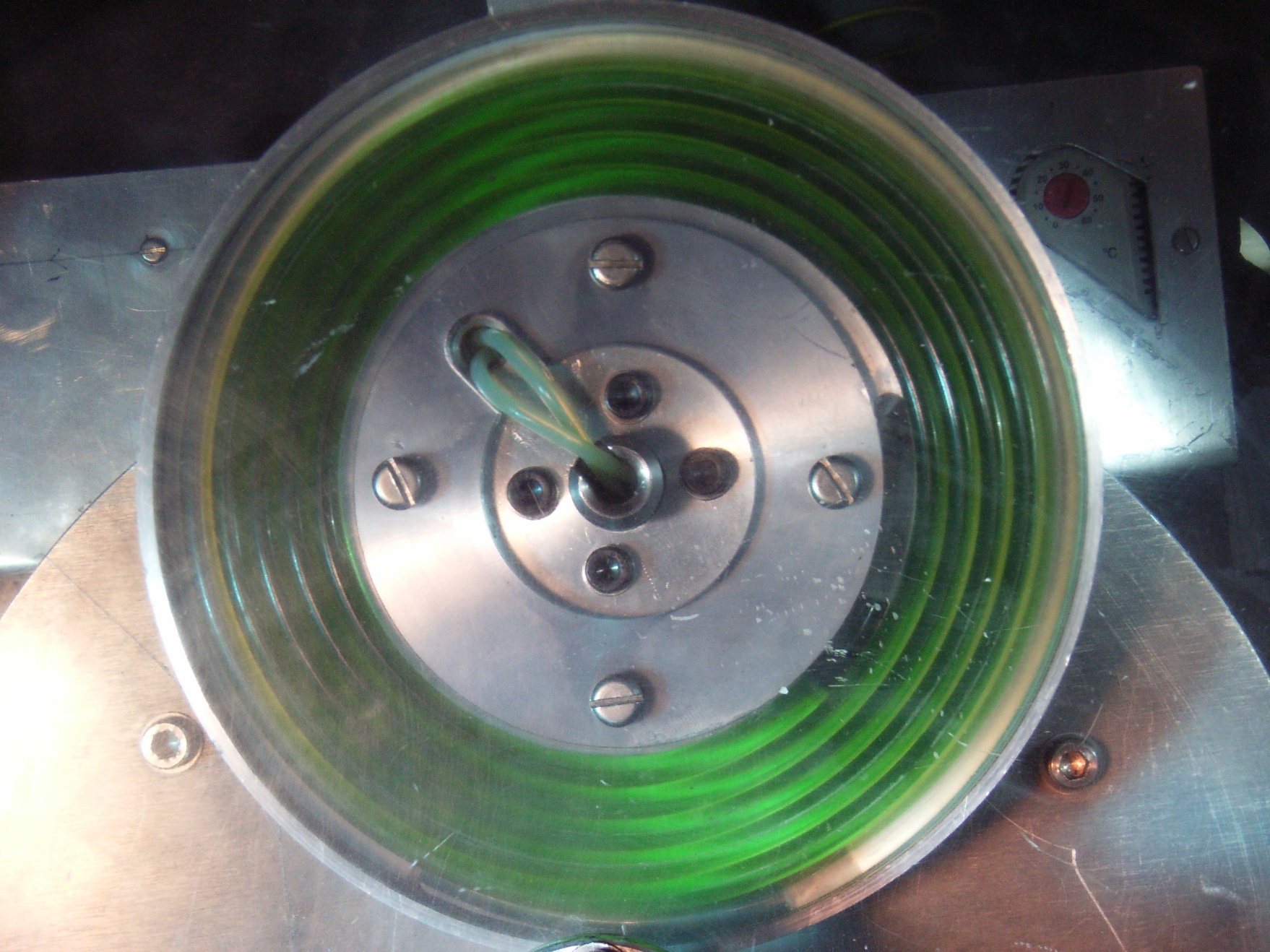 |
| S4-pt2-27  The dynamic image for the whole column  At time t=13.0 min  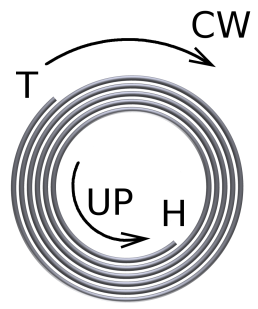 | 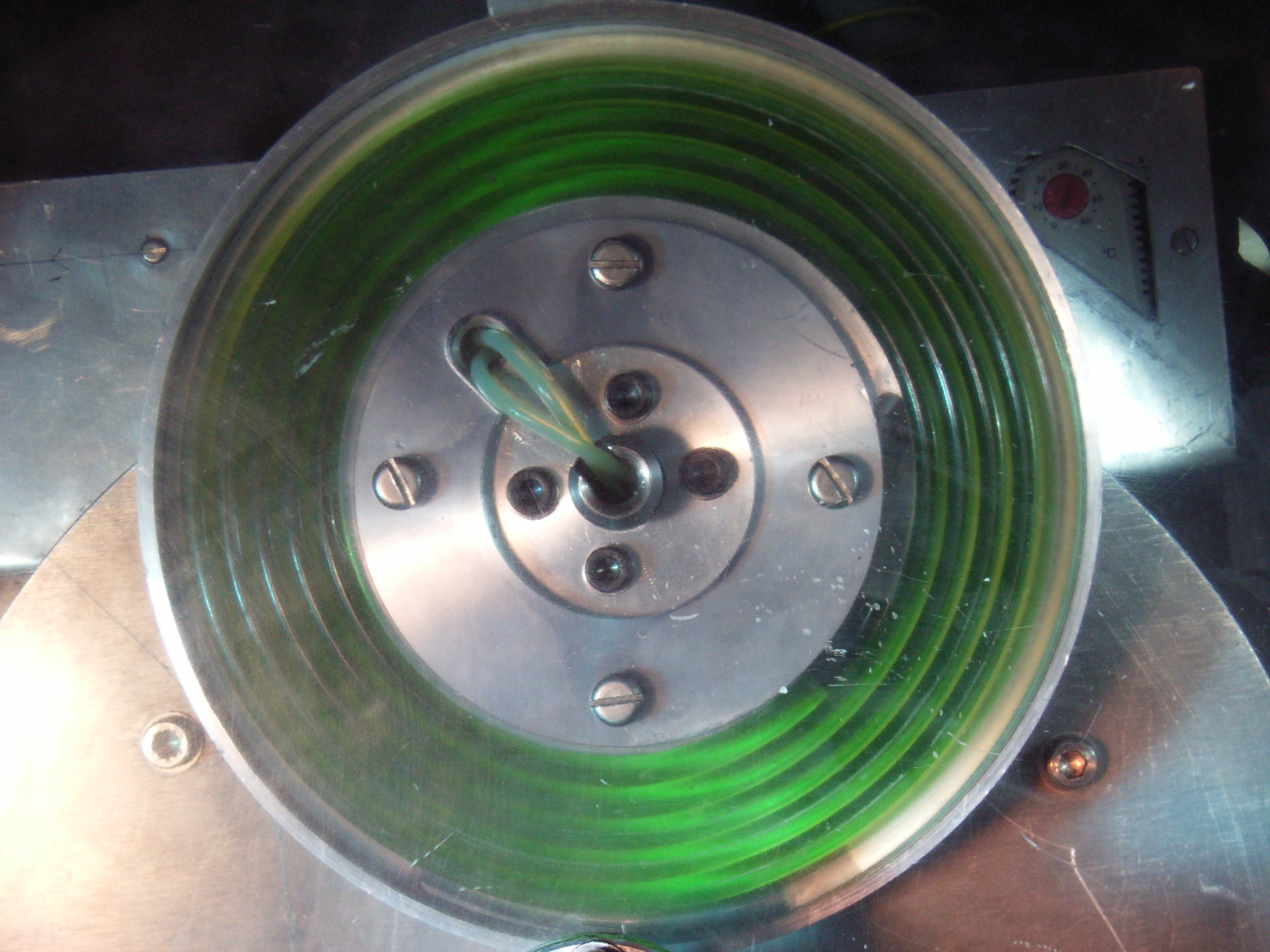 |
| S4-pt2-28  The dynamic image for the whole column  At time t=13.5 min  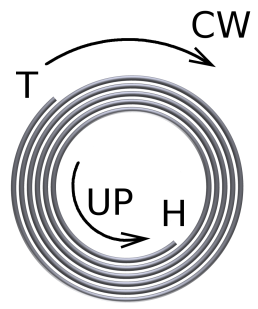 | 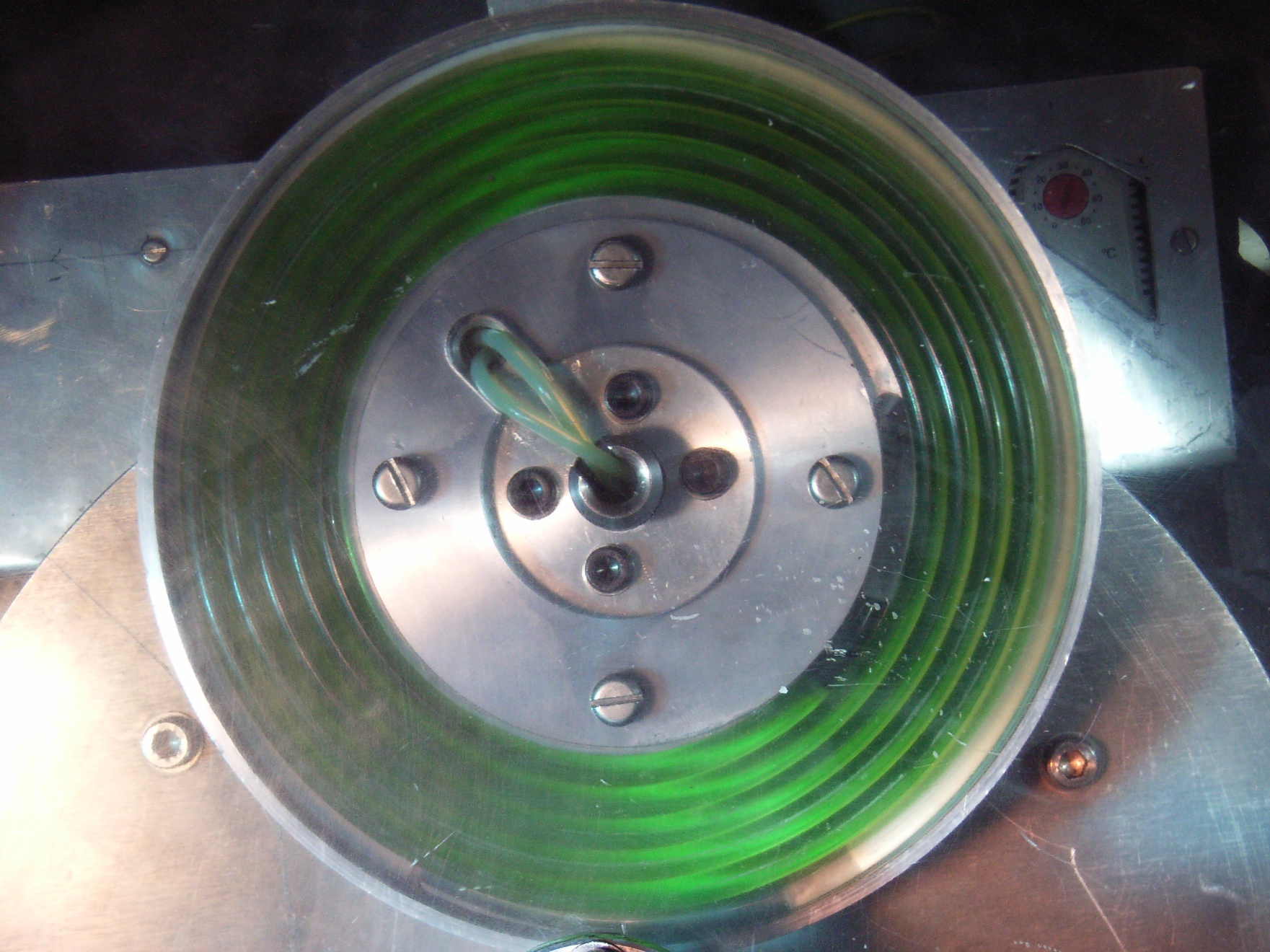 |
