# Supplementary material for: Visualisation of J-type counter-current chromatography: A route to understand hydrodynamic phase distribution and retention
Source: J Chromatogr A. 2012 May 25;1239(15):10–21. doi: 10.1016/j.chroma.2012.03.039 (PMC3405518; doi:10.1016/j.chroma.2012.03.039)
Supplement: Supplementary file 10 [file mmc10.doc]

**Supplementary material: 9**

Detailed insights into the phase mixing and separation situation for the ATPS contained in a spiral tubing and undergoing type-J synchronous centrifugal planetary motion.

Flow mode: L-I-H at 8 ml/min (i.e. the lower phosphate phase as the mobile phase which flows from the inside terminal of the spiral column towards the column periphery). This experimental condition is denoted as A4 in Table 1.

The ATPS is formed by 18% (w/w) PEG 1000 and 18% (w/w) K_2_HPO_4_ in deionised water. The rotation speed of the centrifuge was at 800 rpm.

An illustration is shown in the left column for explaining the dynamic situation where the image in the right column was taken.

| S9-2  The dynamic image for the whole column  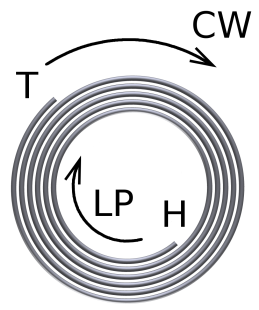 | 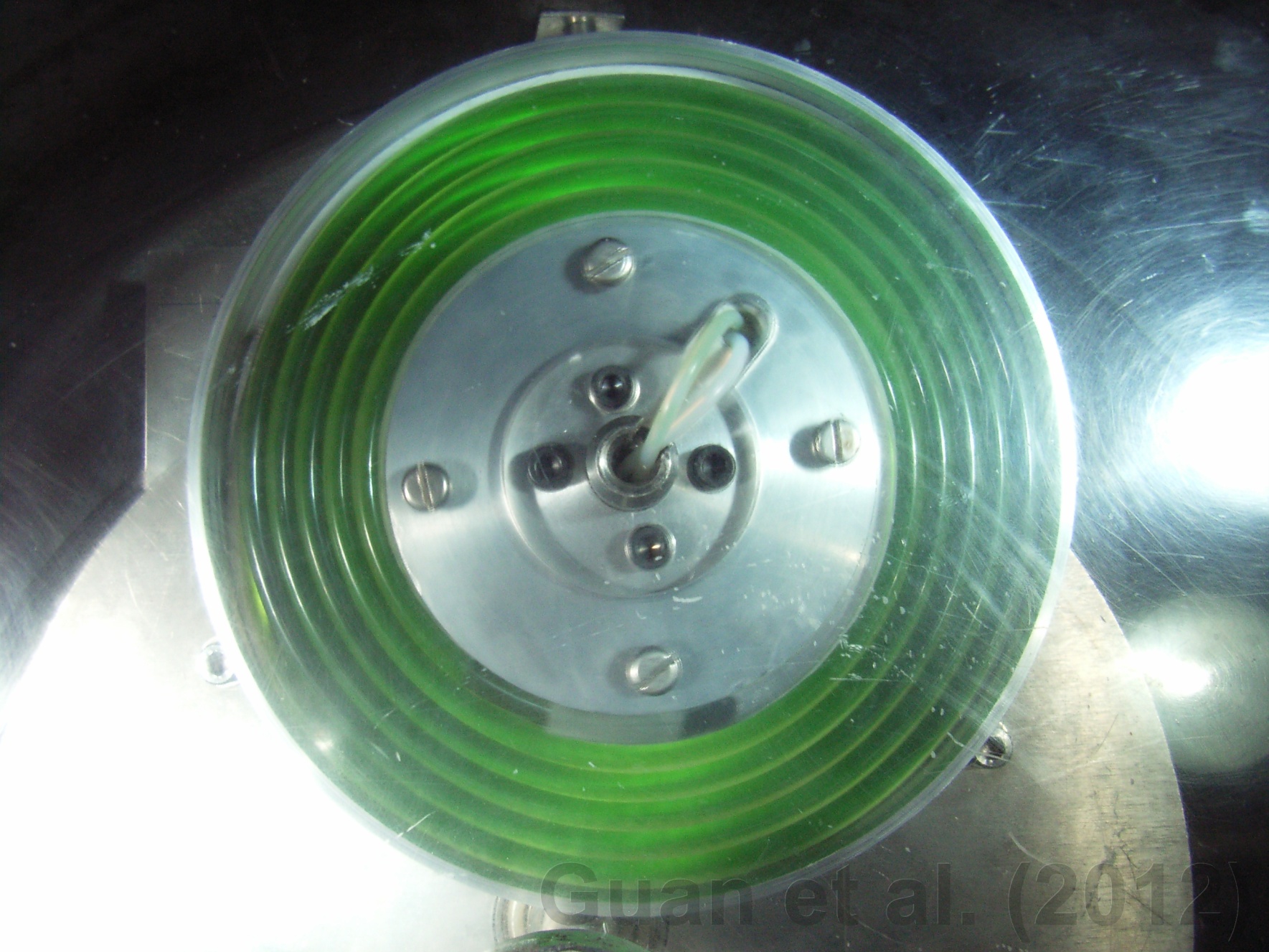 |
| --- | --- |
| S9-3  The static image for the whole column.  The time was at the end of experiment when rotation was terminated.  This shows phase distribution and stationary phase retention during the prior planetary motion.  This image was not taken under stroboscopic lighting | 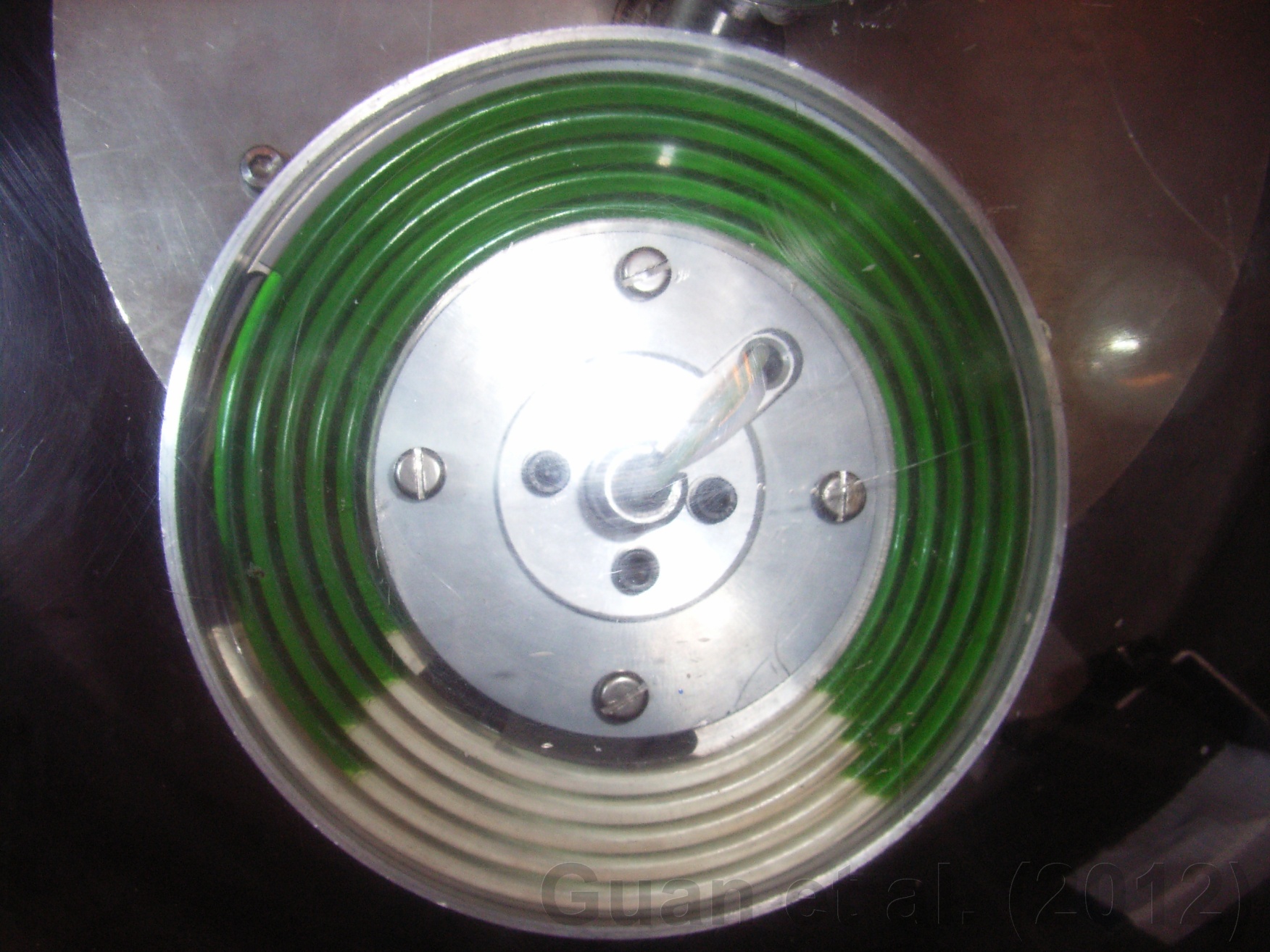 |
| S9-4  A dynamic image for the focused part of the column (shown below)  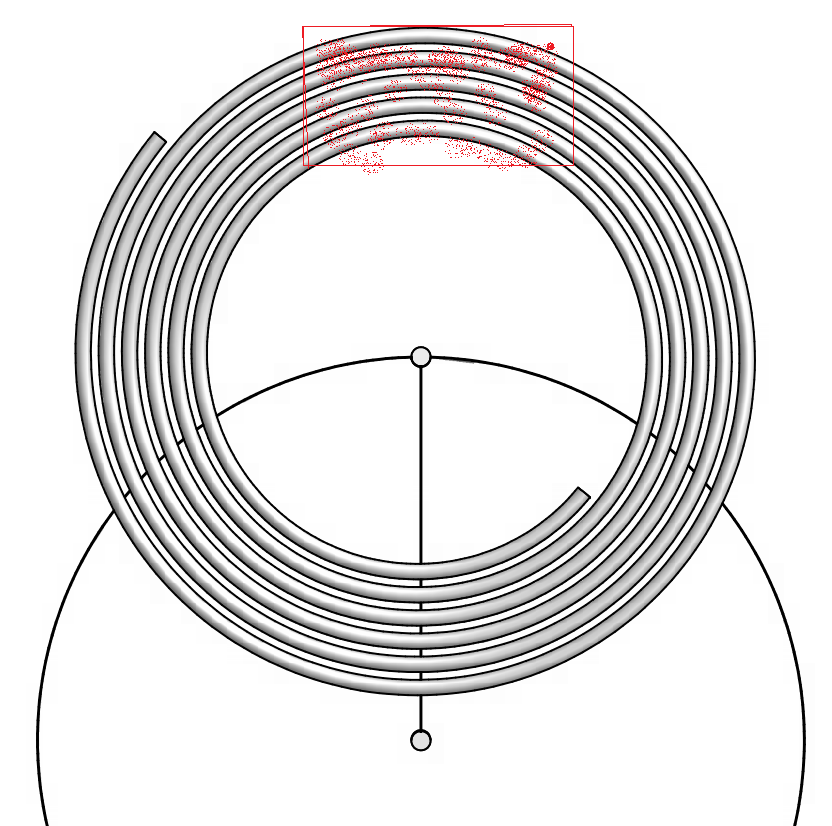 | 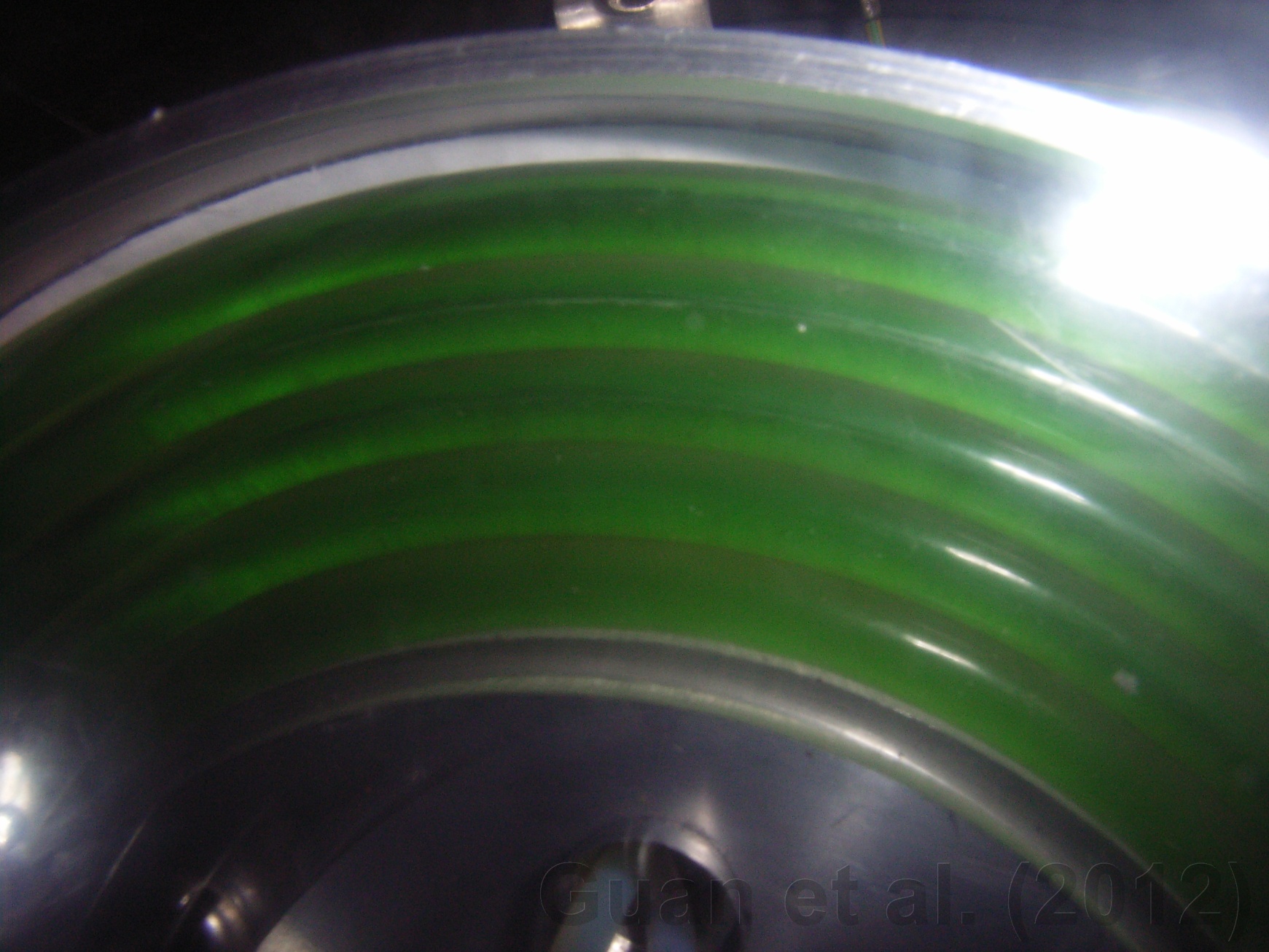 |
| S9-5  A dynamic image for the focused part of the column (shown below)  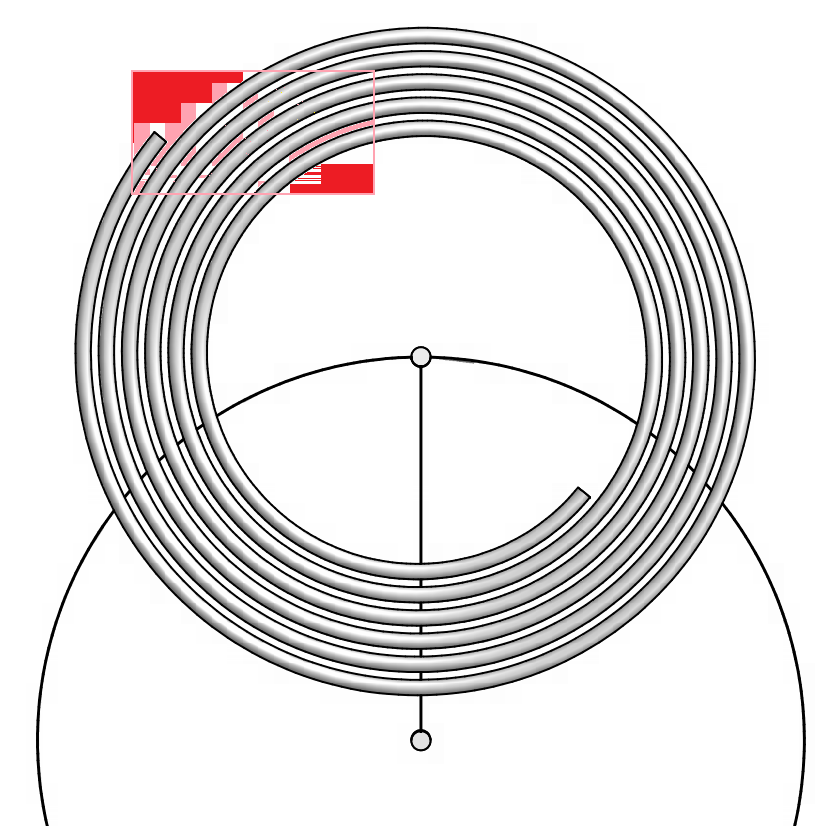 | 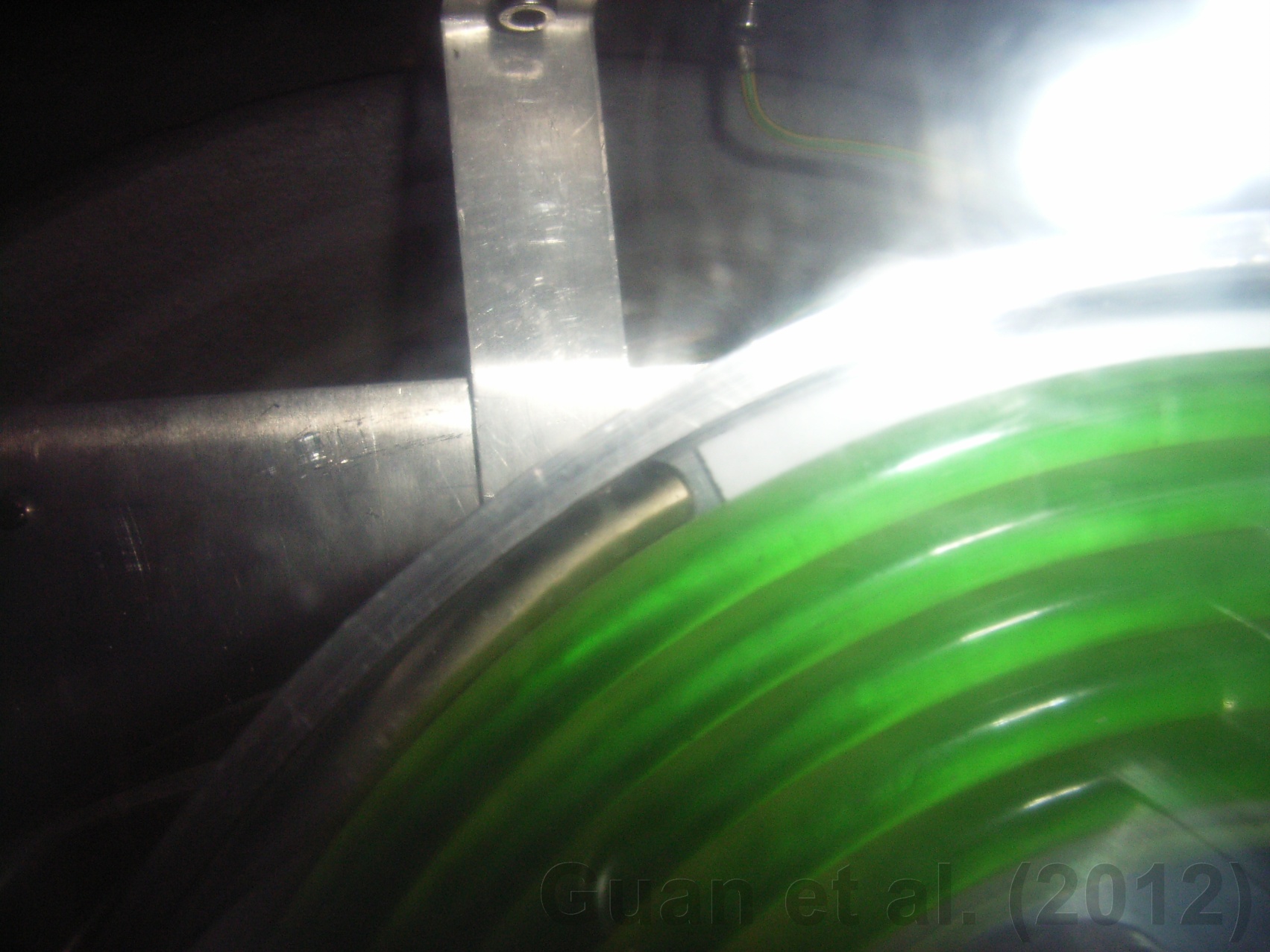 |
| S9-6  A dynamic image for the focused part of the column (shown below)  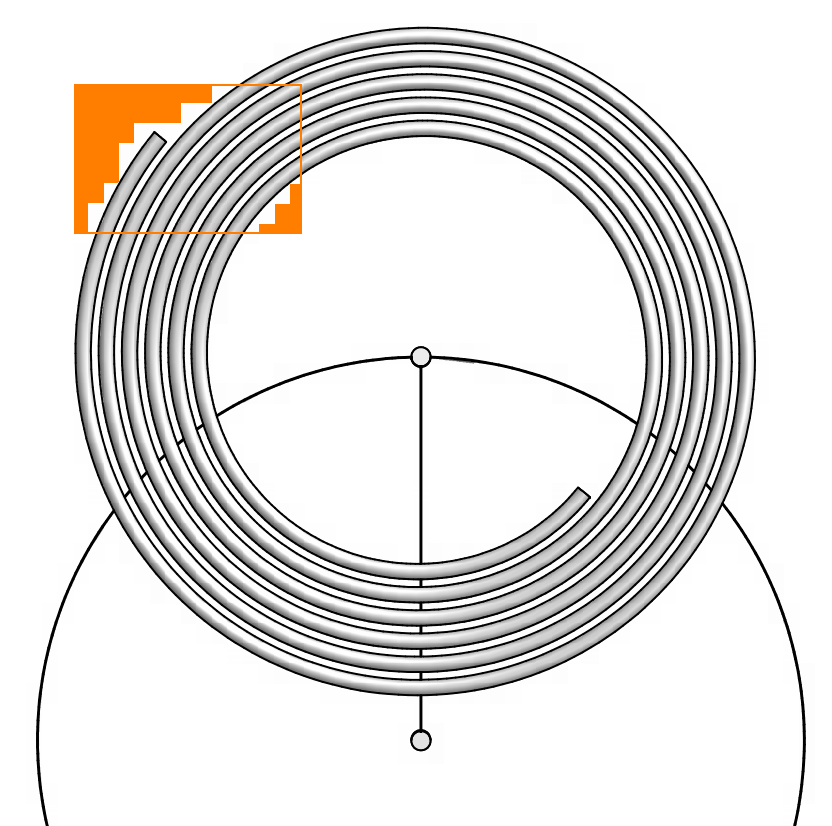 | 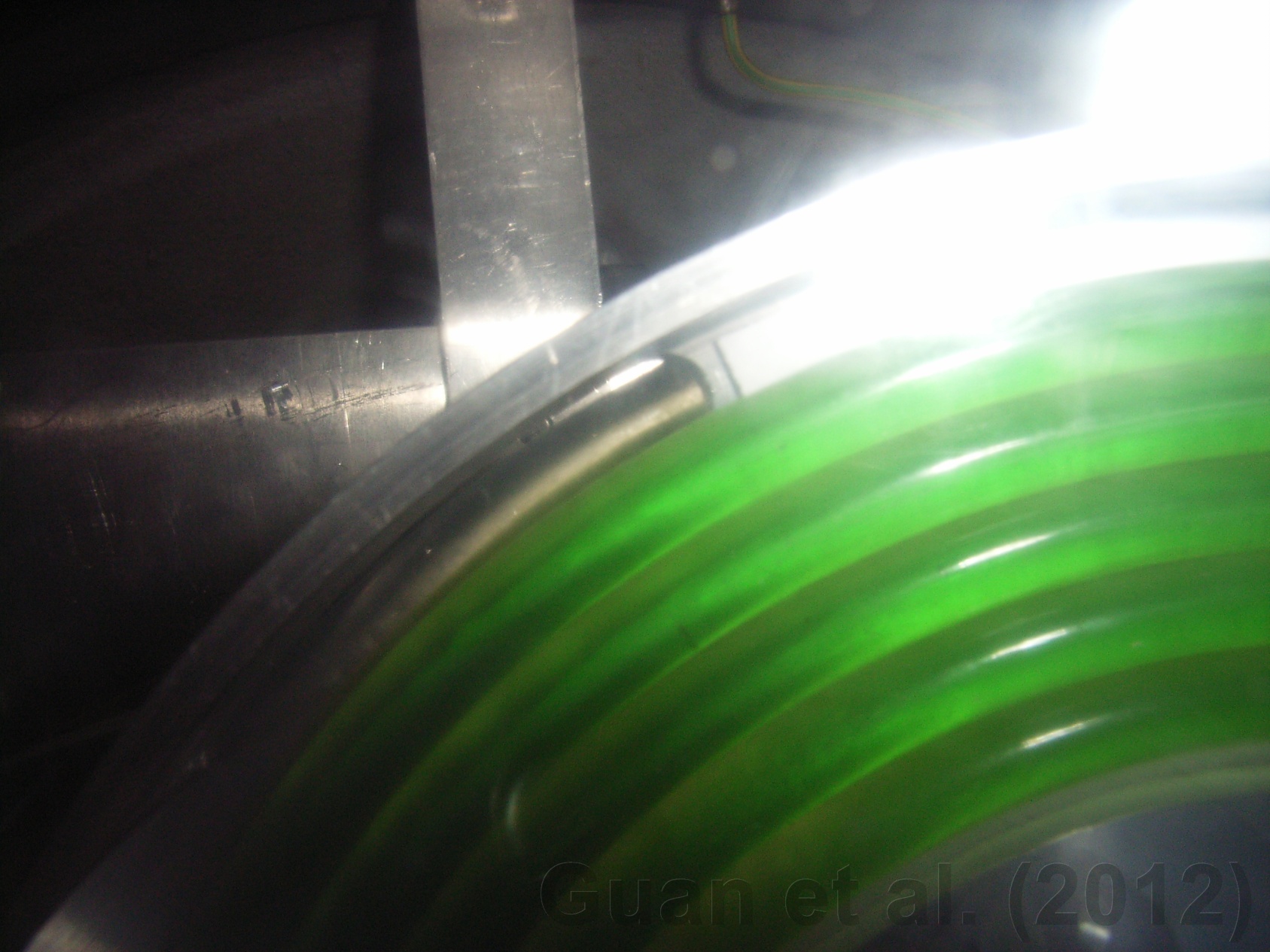 |
| S9-7  A dynamic image for the focused part of the column (shown below)  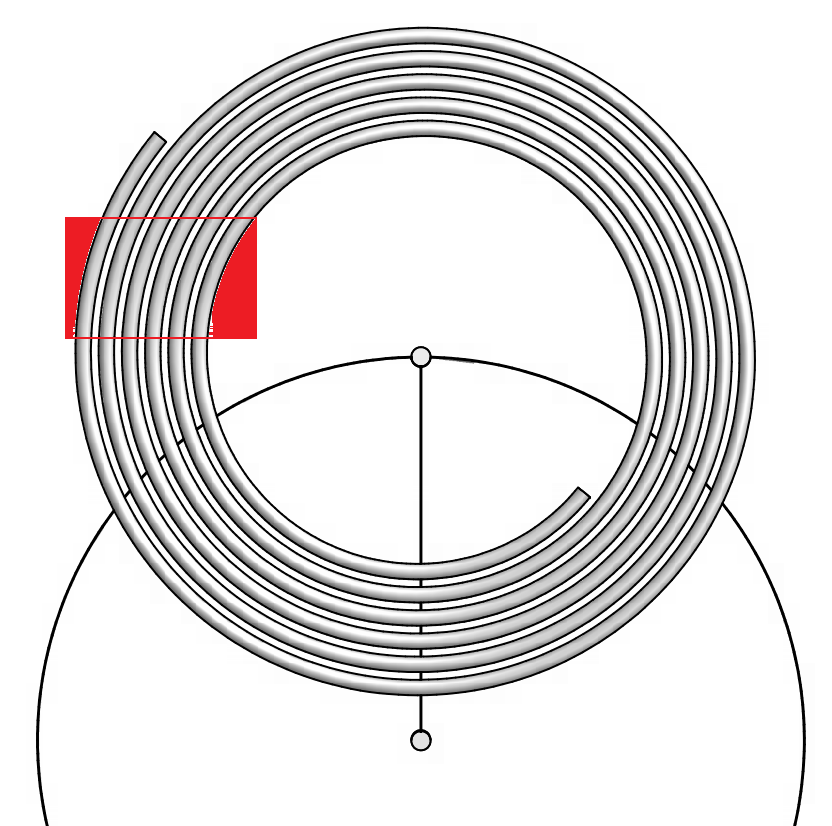 | 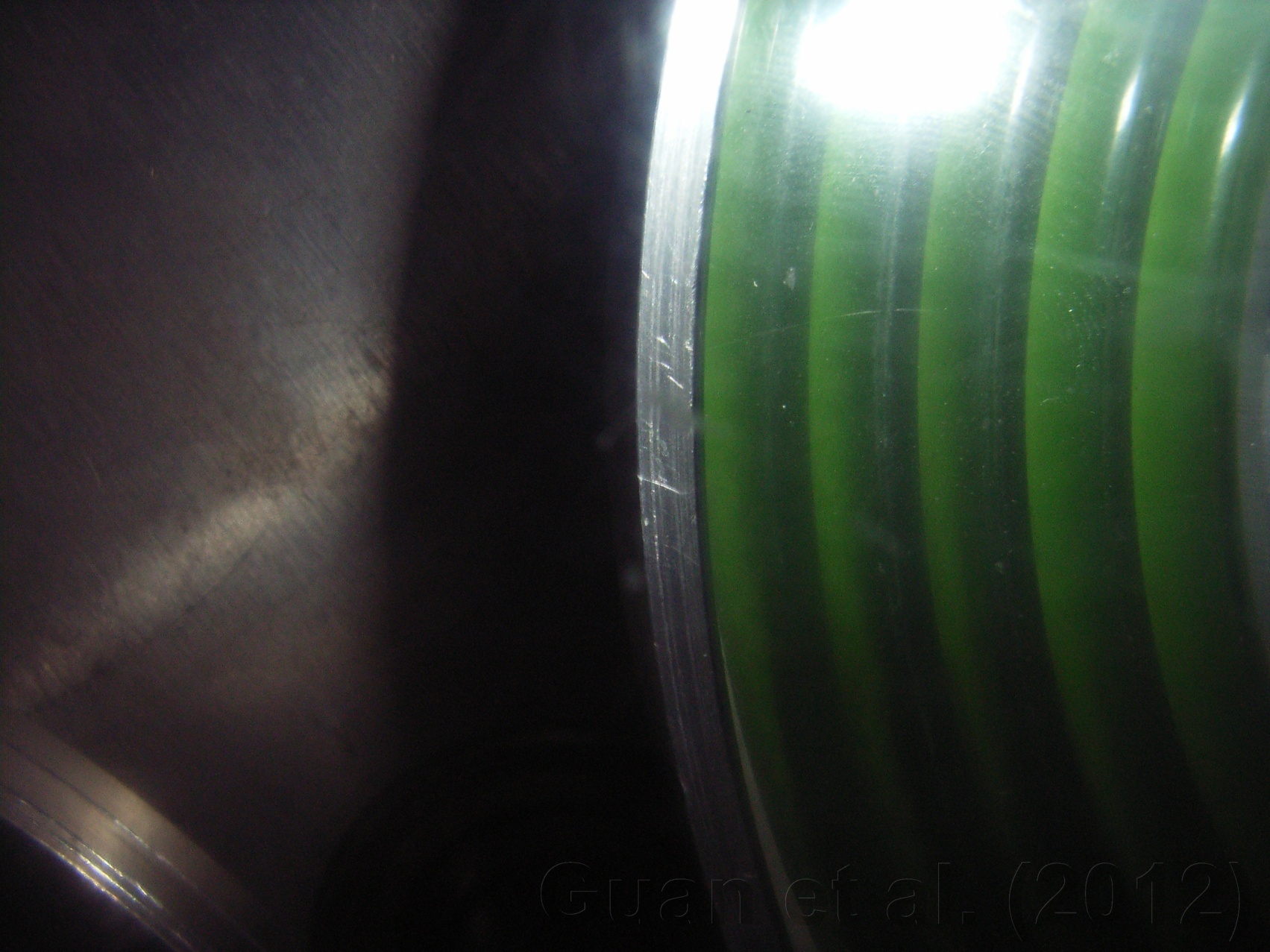 |
| S9-8  A dynamic image for the focused part of the column (shown below)  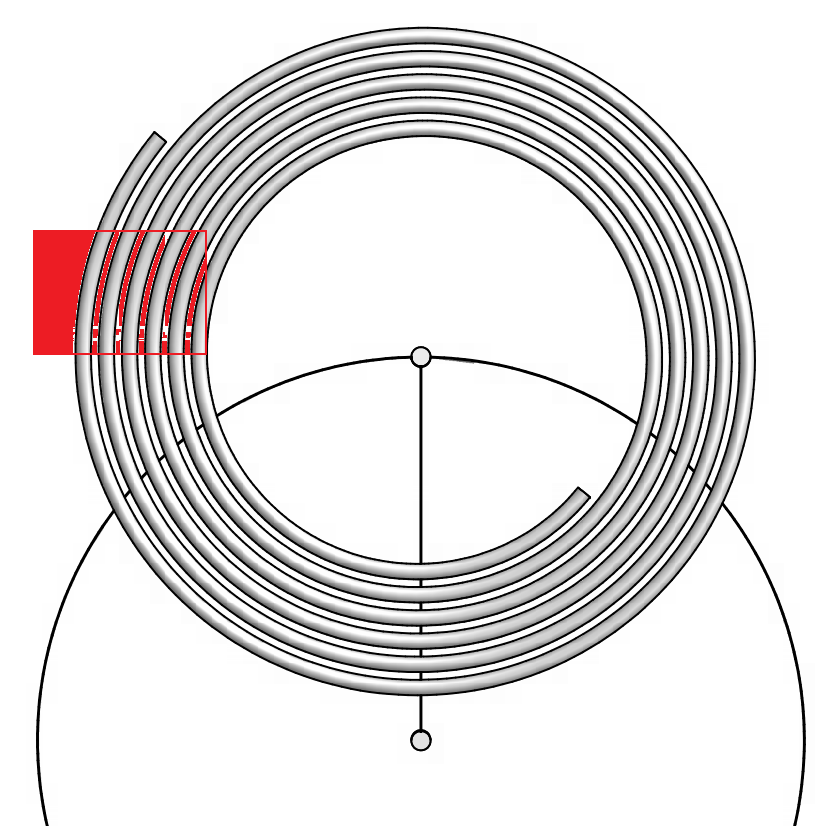 | 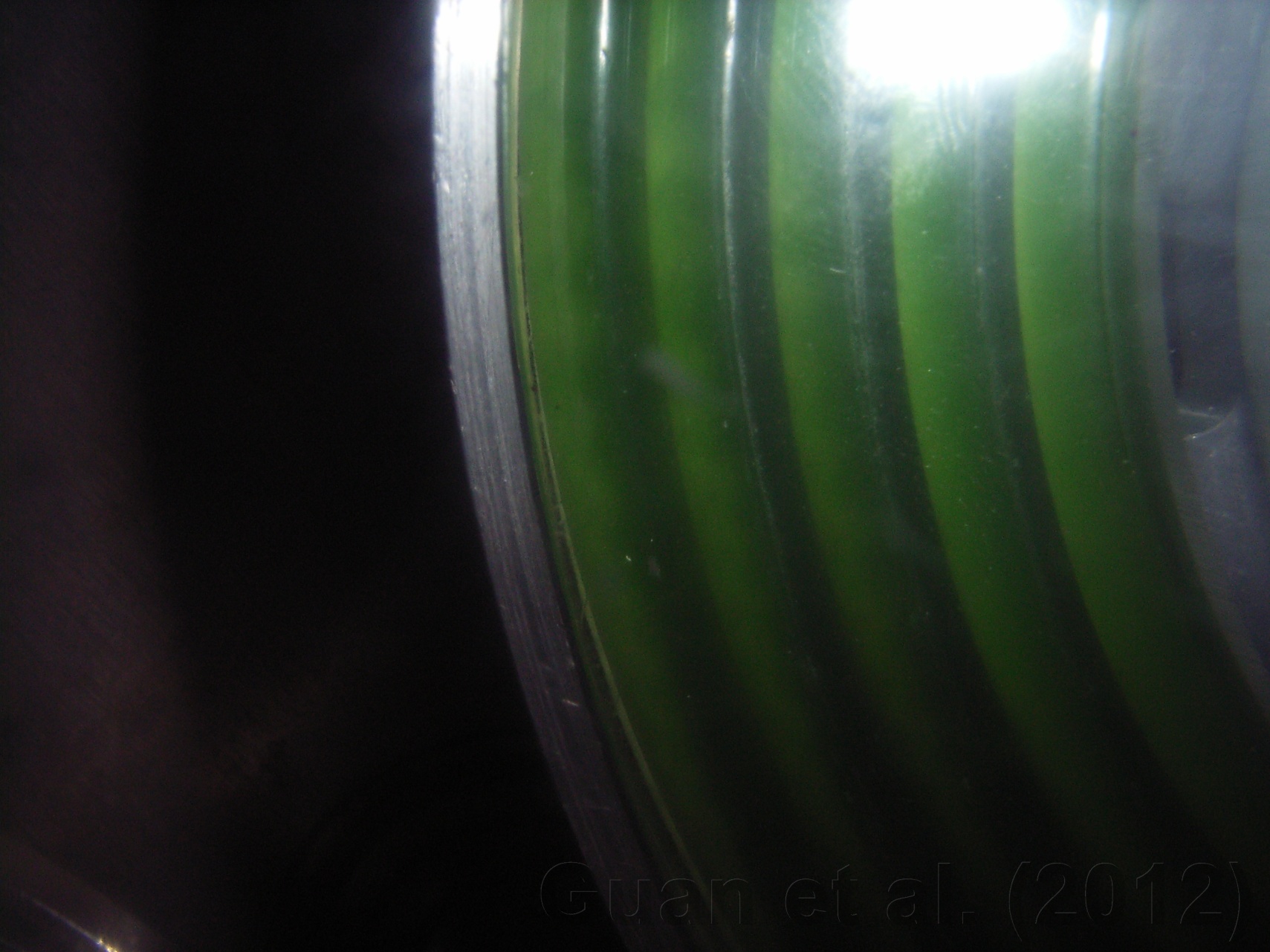 |
| S9-9  A dynamic image for the focused part of the column (shown below)  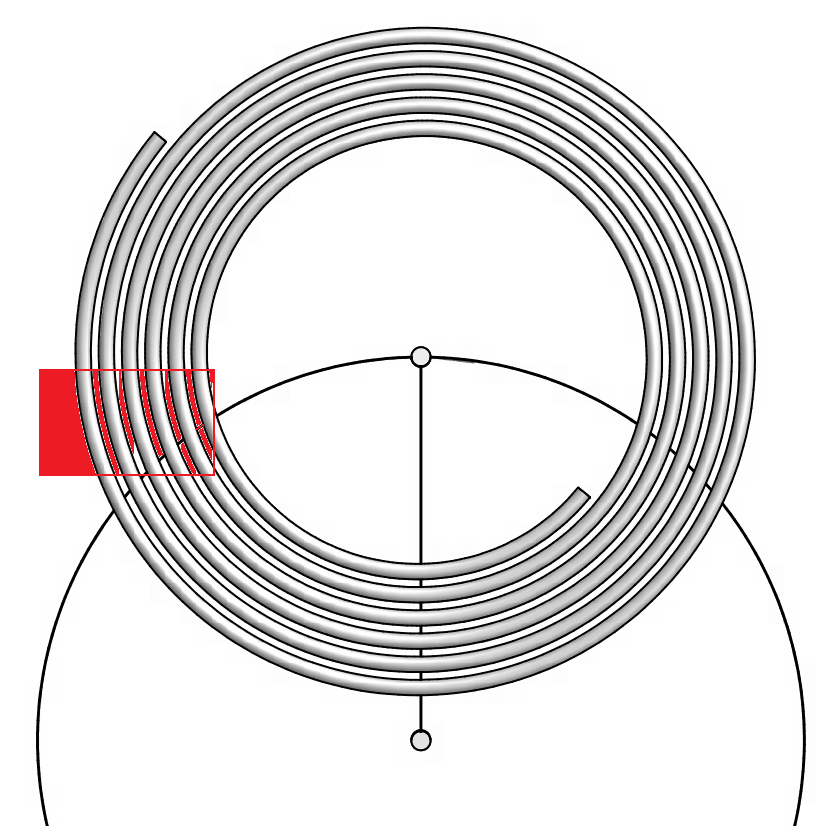 | 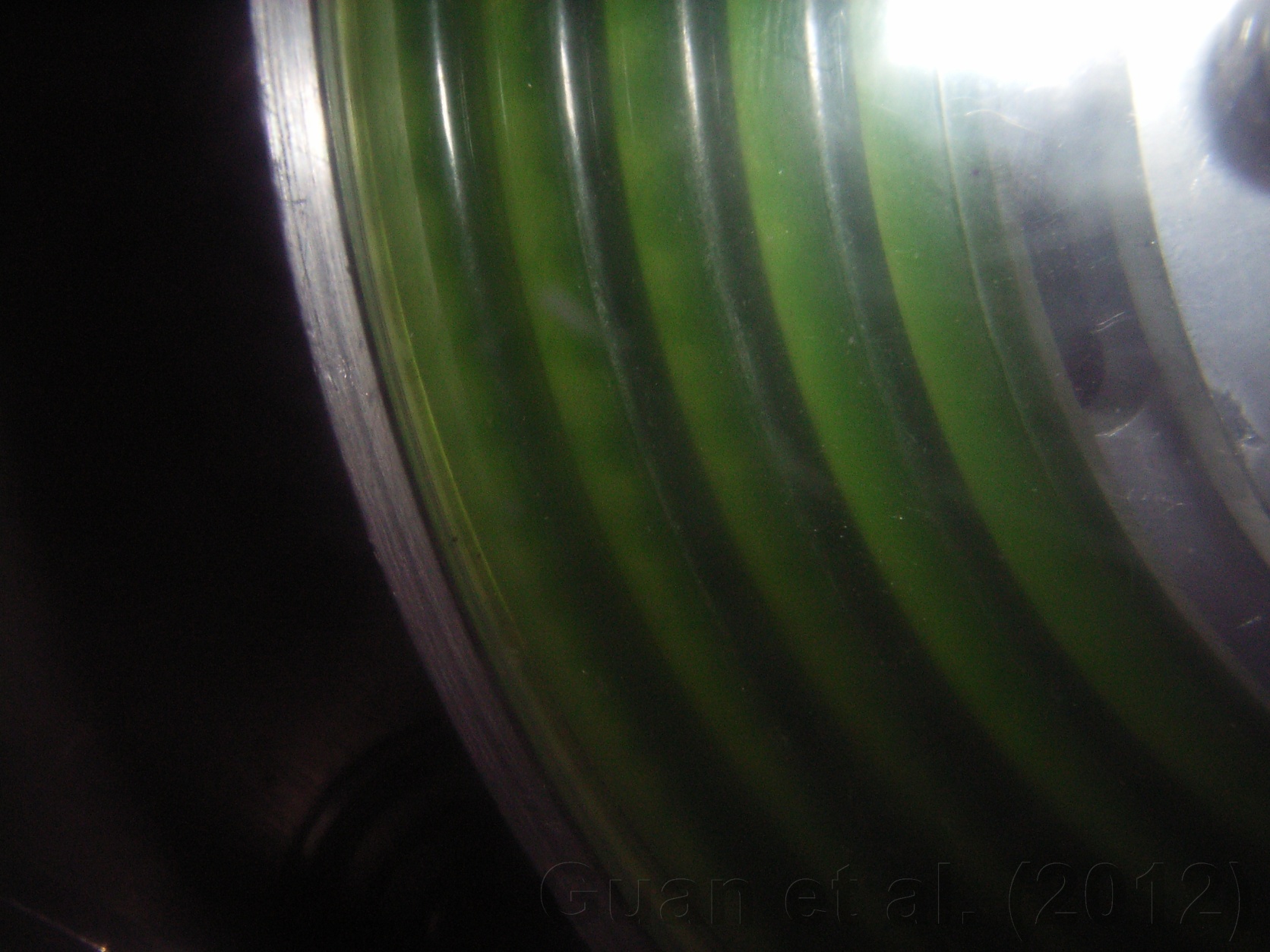 |
| S9-10  A dynamic for the focused part of the column (shown below)  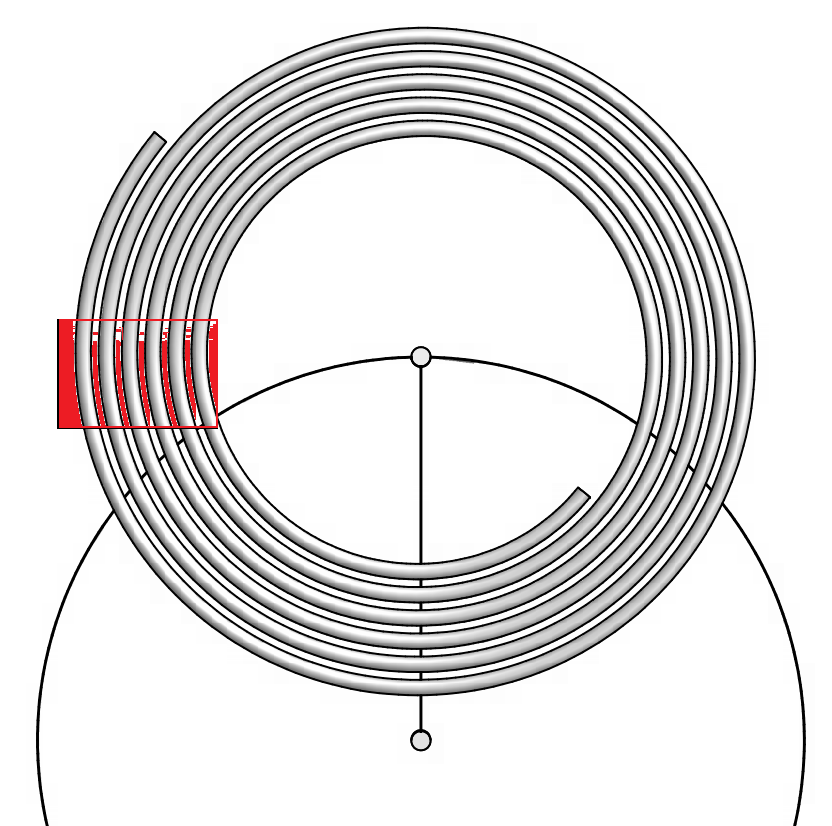 | 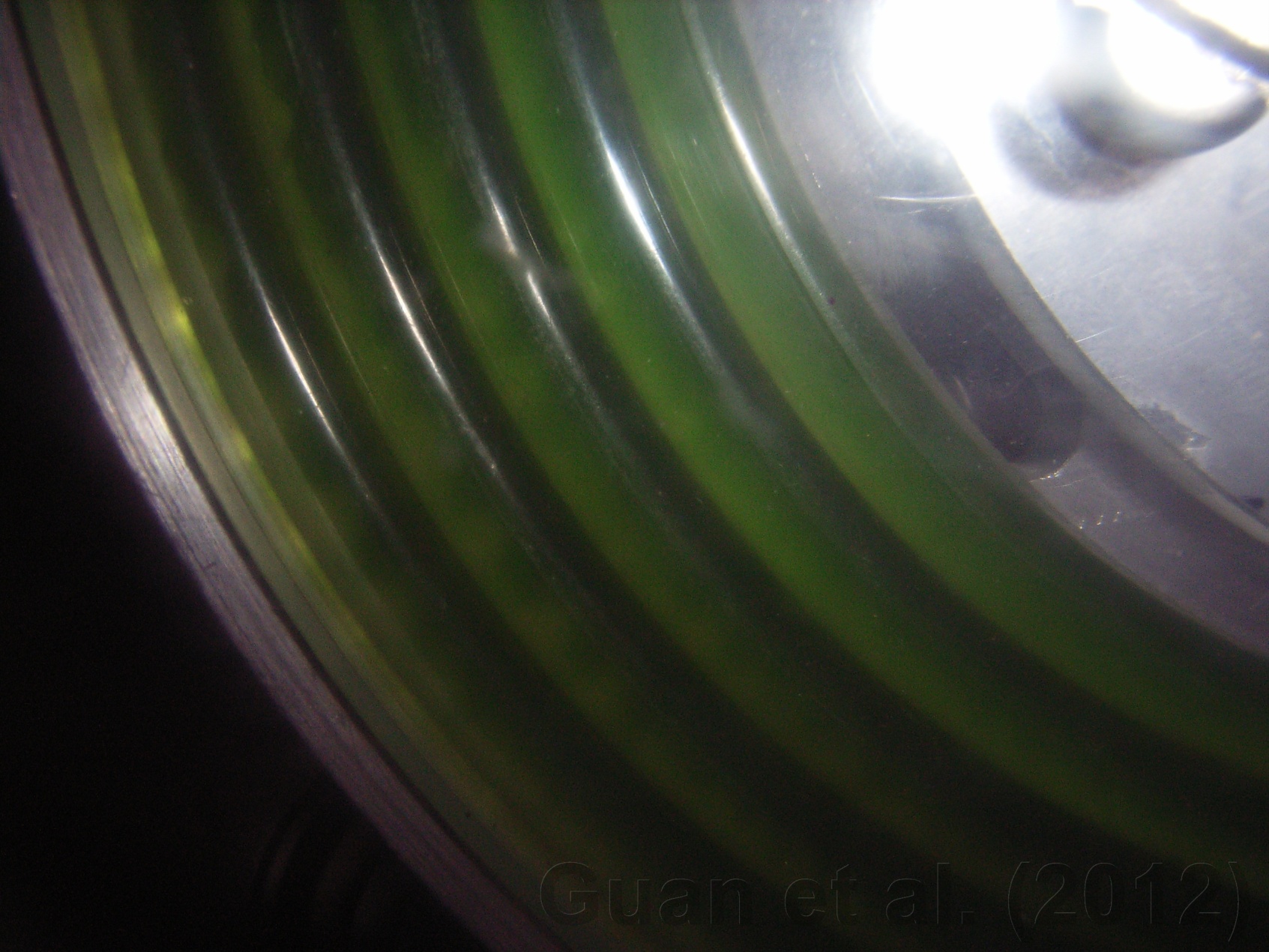 |
| S9-11  A dynamic image for the focused part of the coil (shown below)  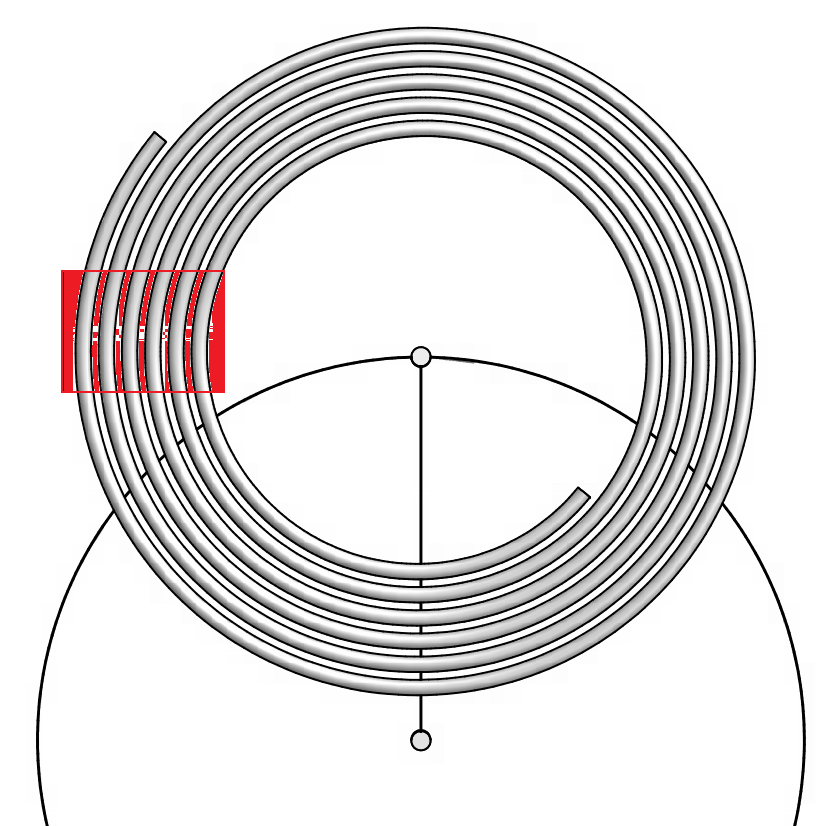 | 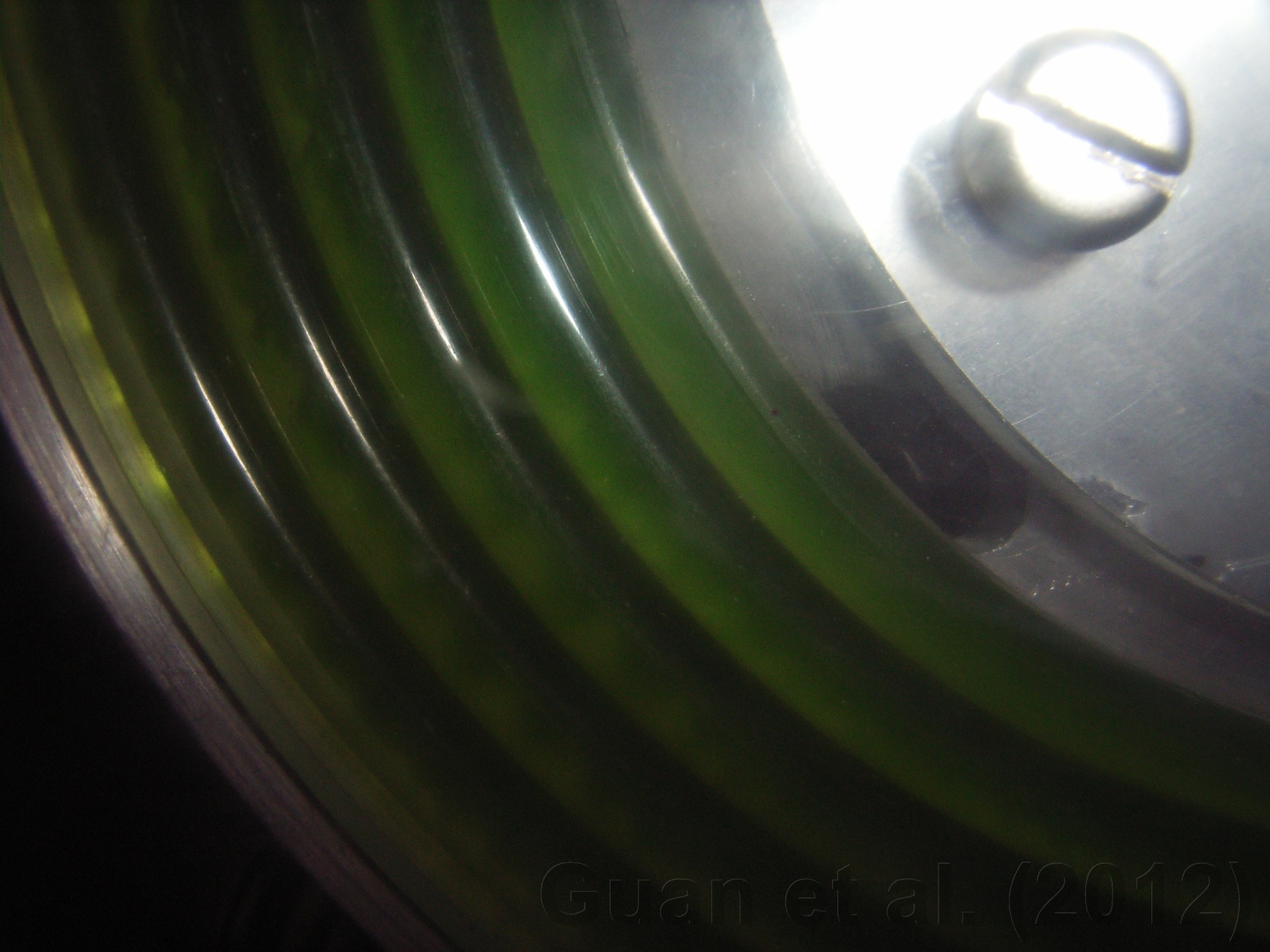 |
| S9-12  A dynamic image for the focused part of the column (shown below)  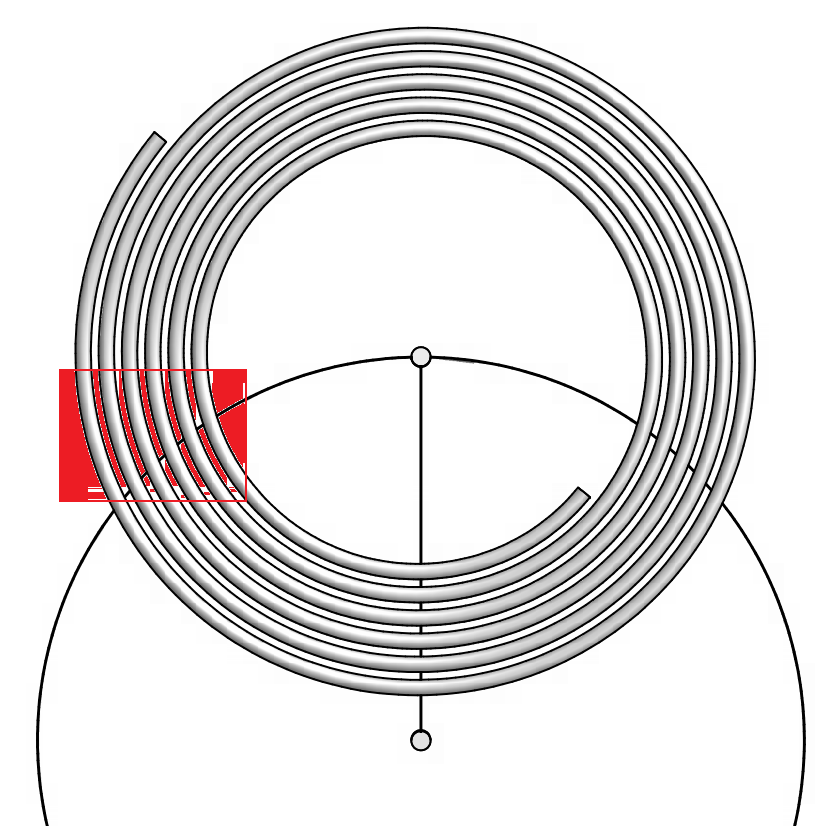 | 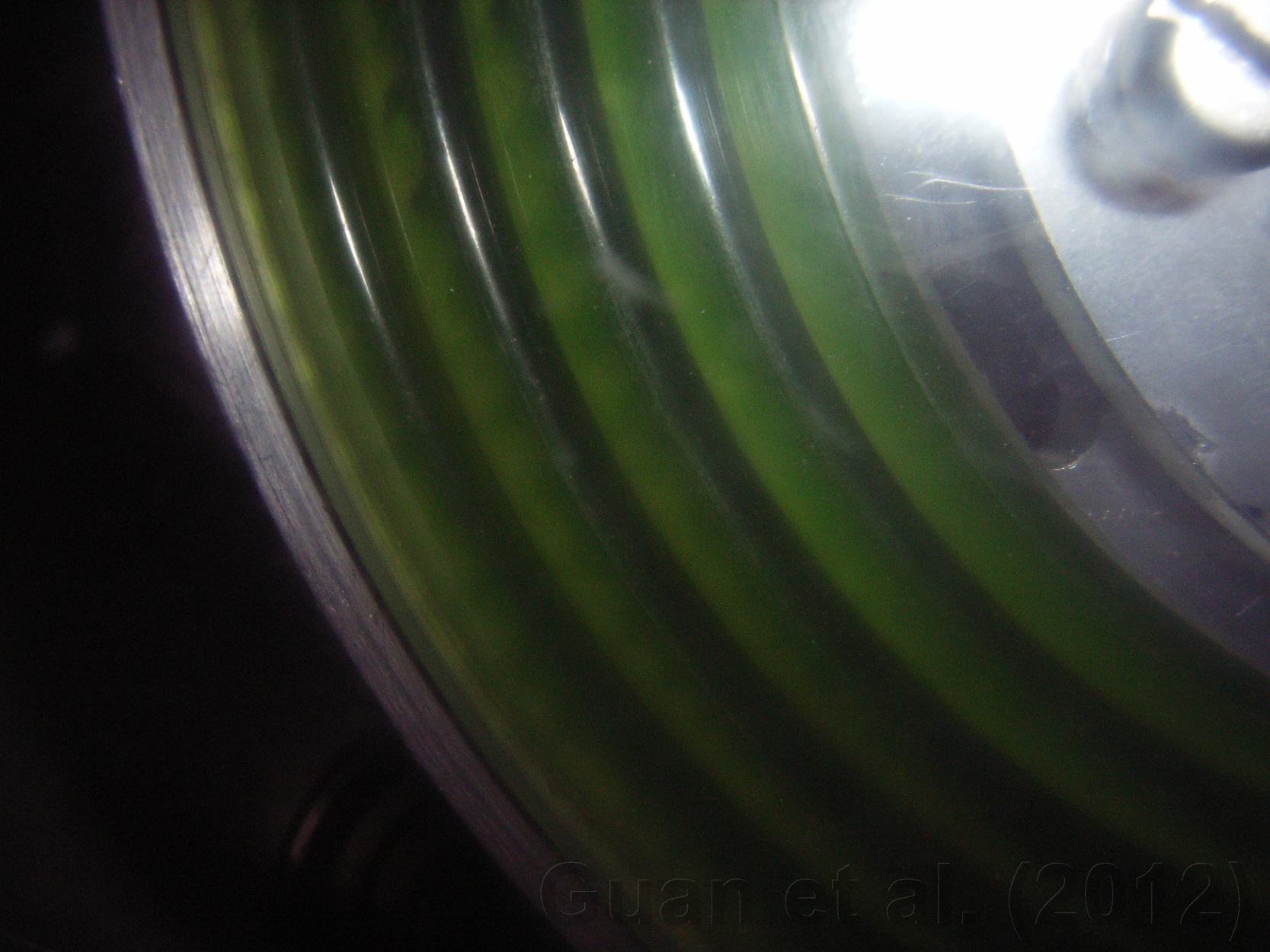 |
| S9-13  A dynamic image for the focused part of the column (shown below)  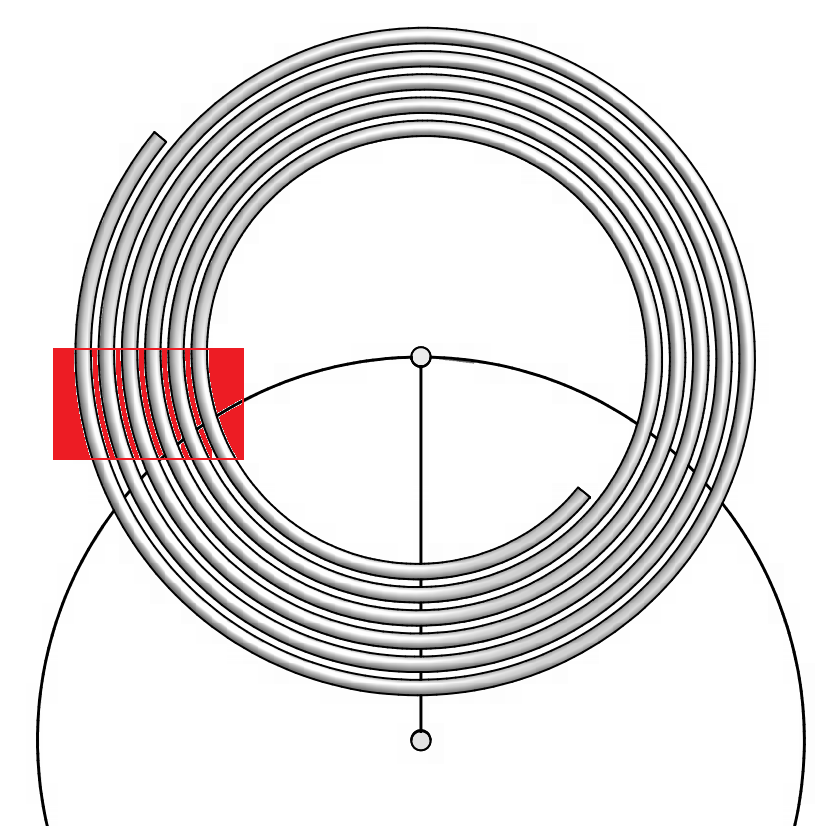 | 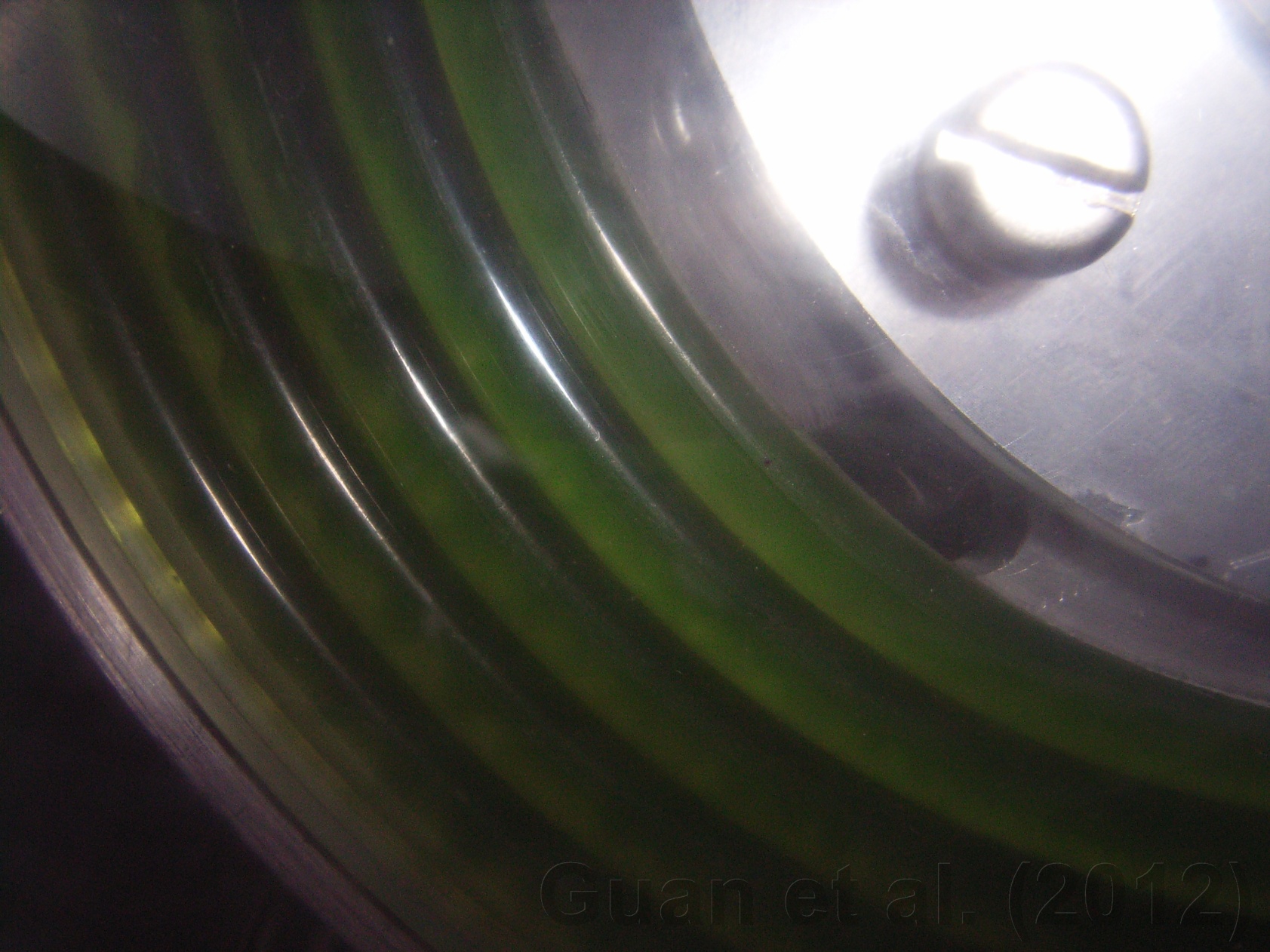 |
| S9-14  A dynamic image for the focused part of the column (shown below)  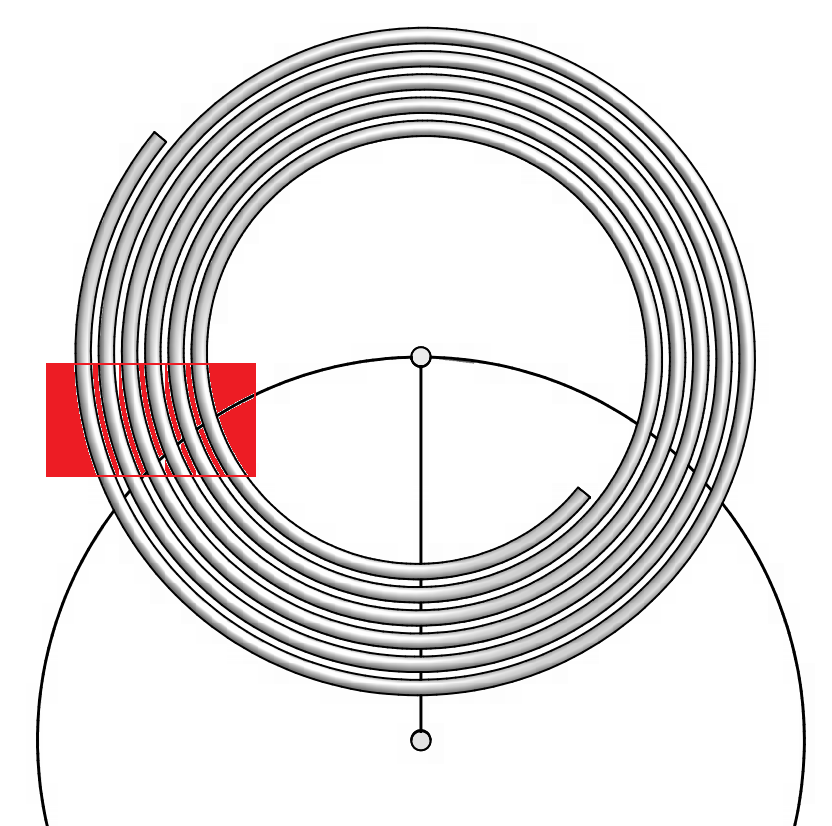 | 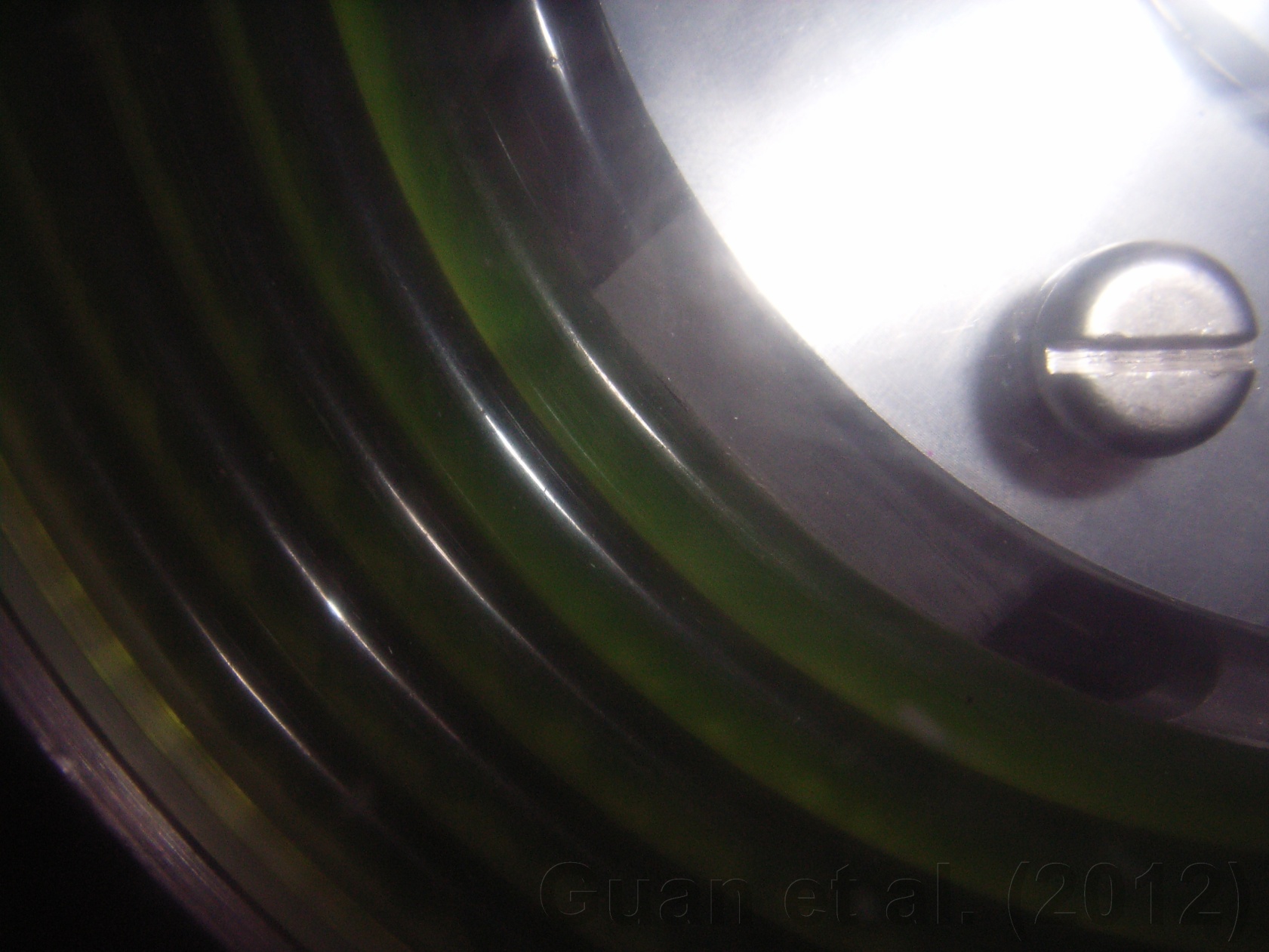 |
| S9-15  A dynamic image for the focused part of the column (shown below)  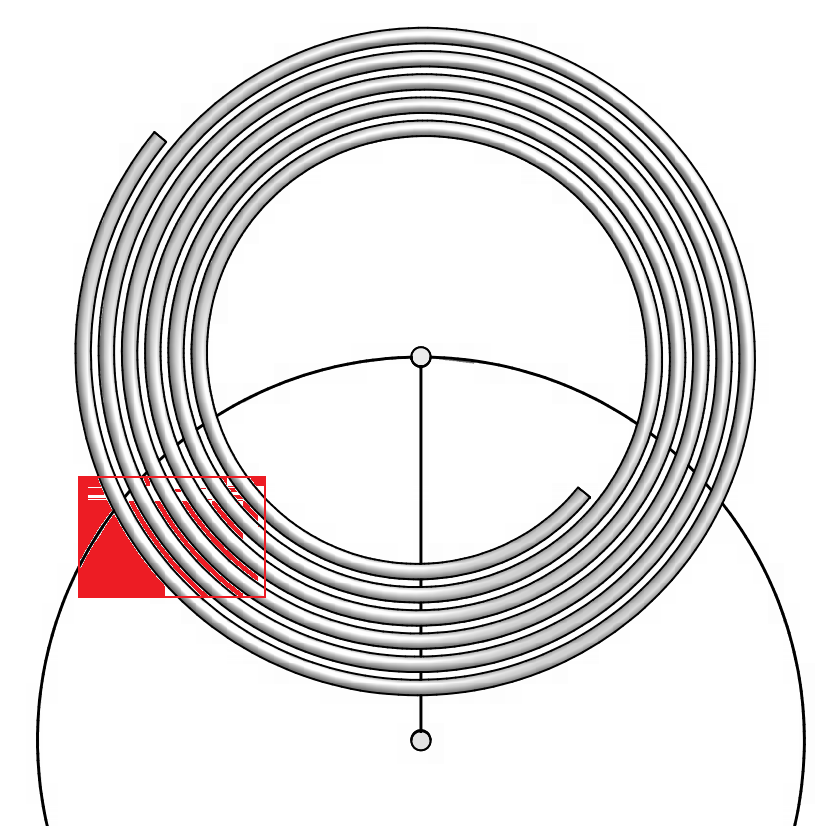 | 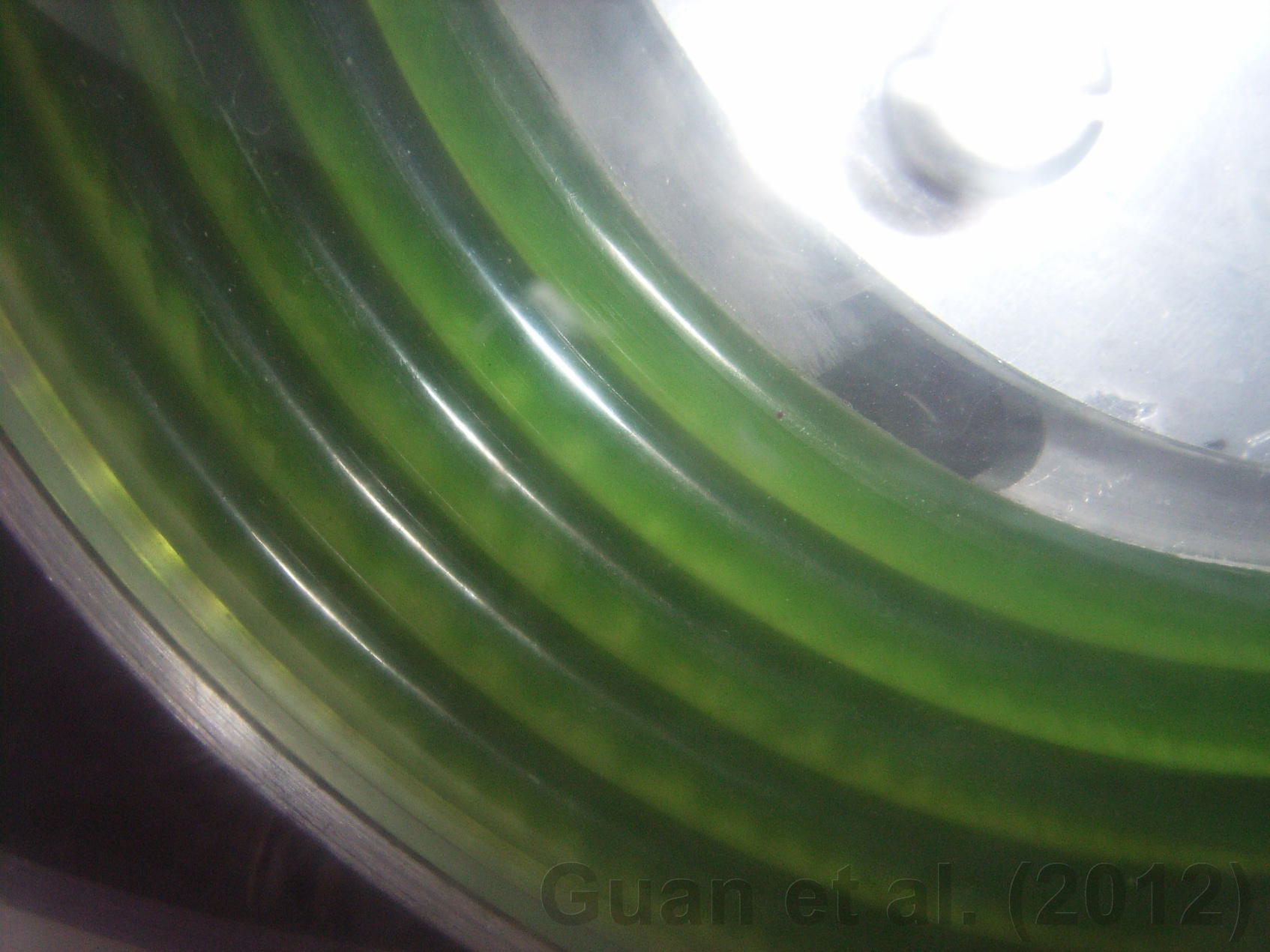 |
| S9-16  A dynamic image for the focused part of the column (shown below)  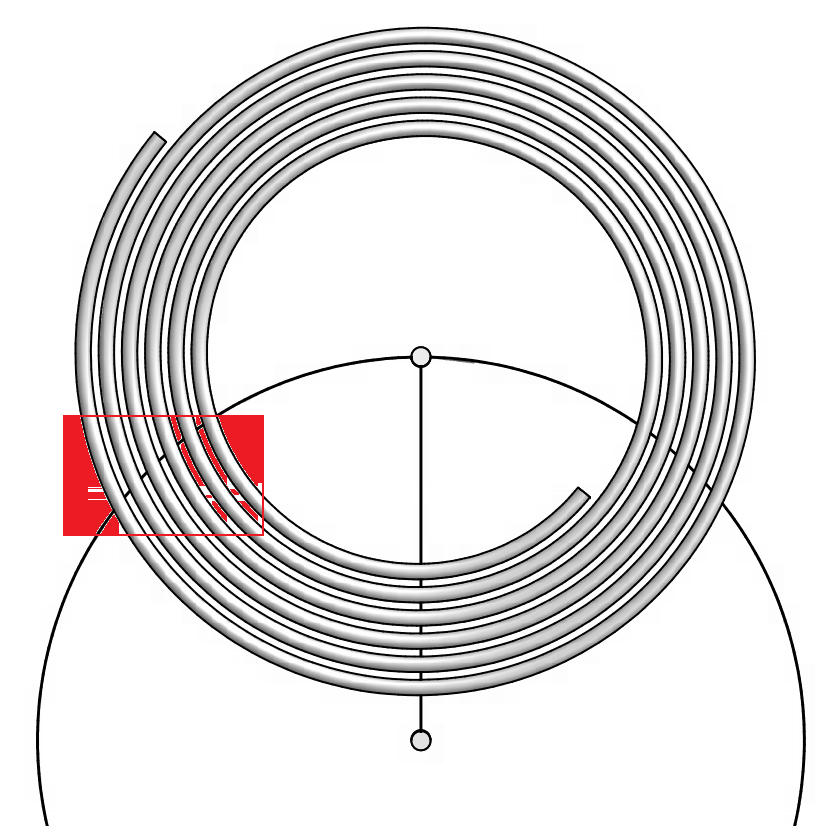 | 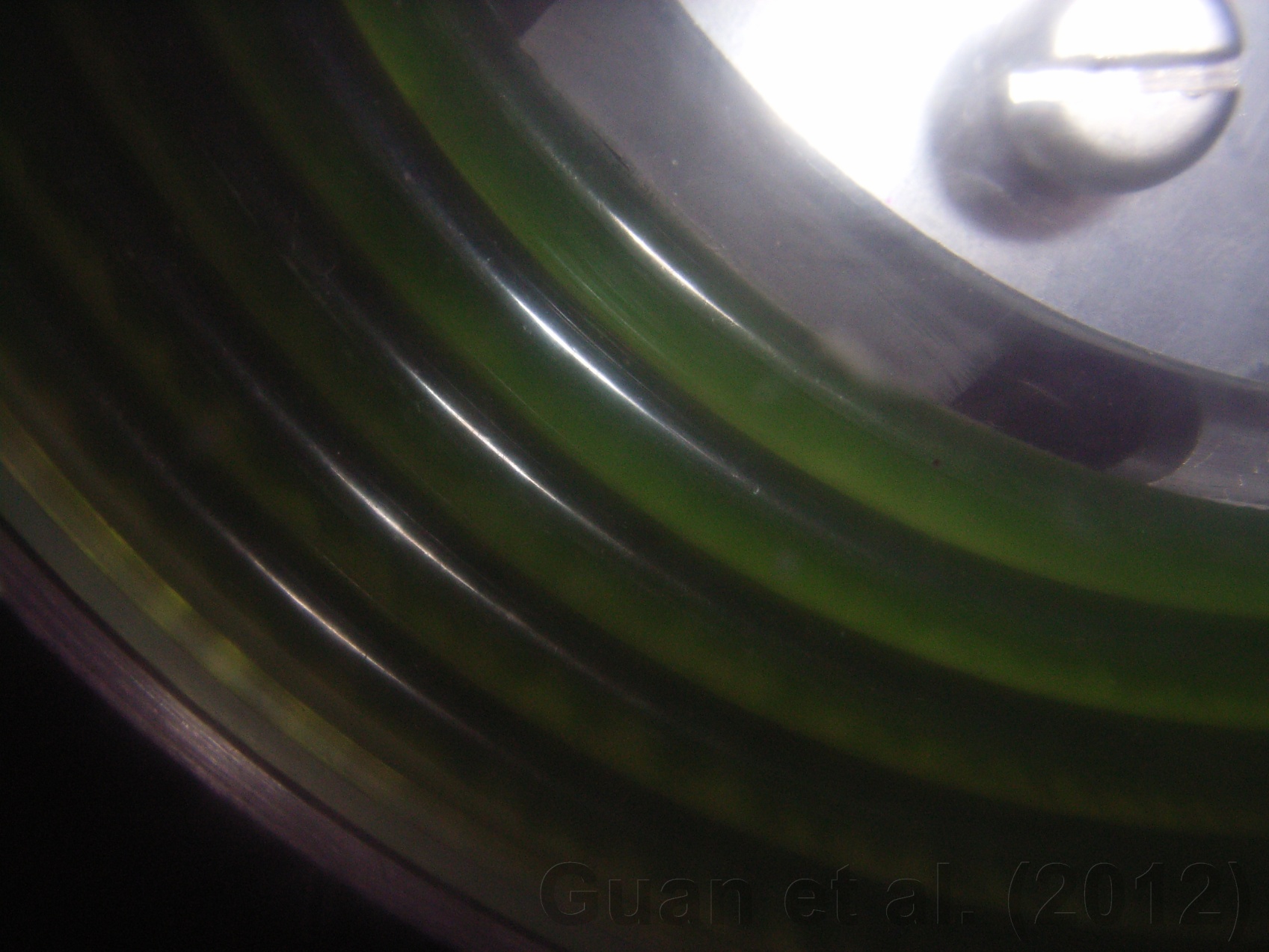 |
| S9-17  A dynamic for the focused part of the column (shown below)  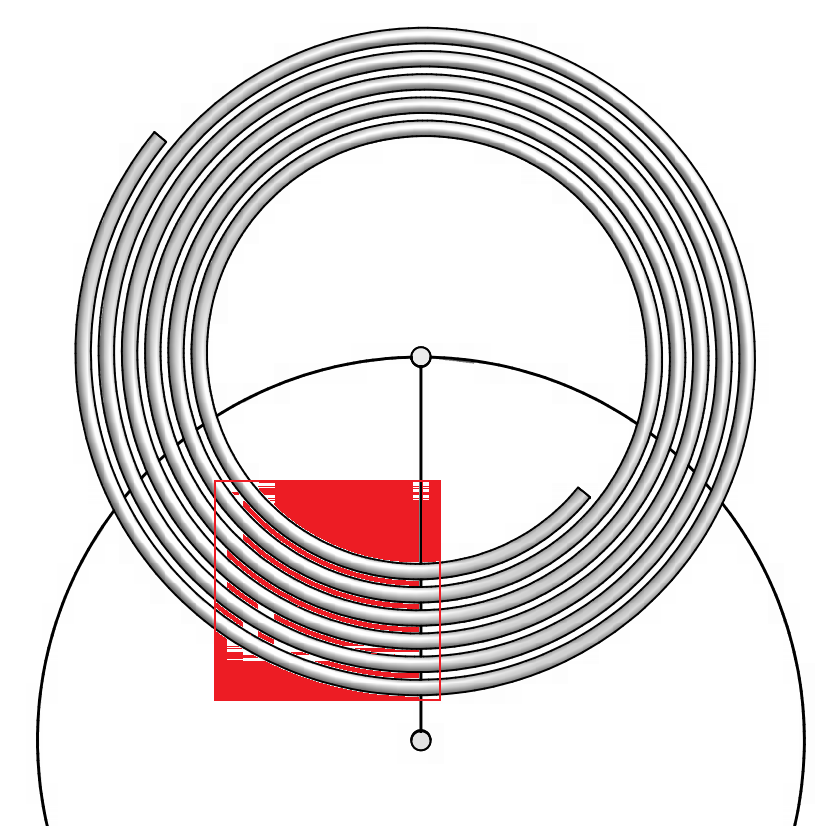 | 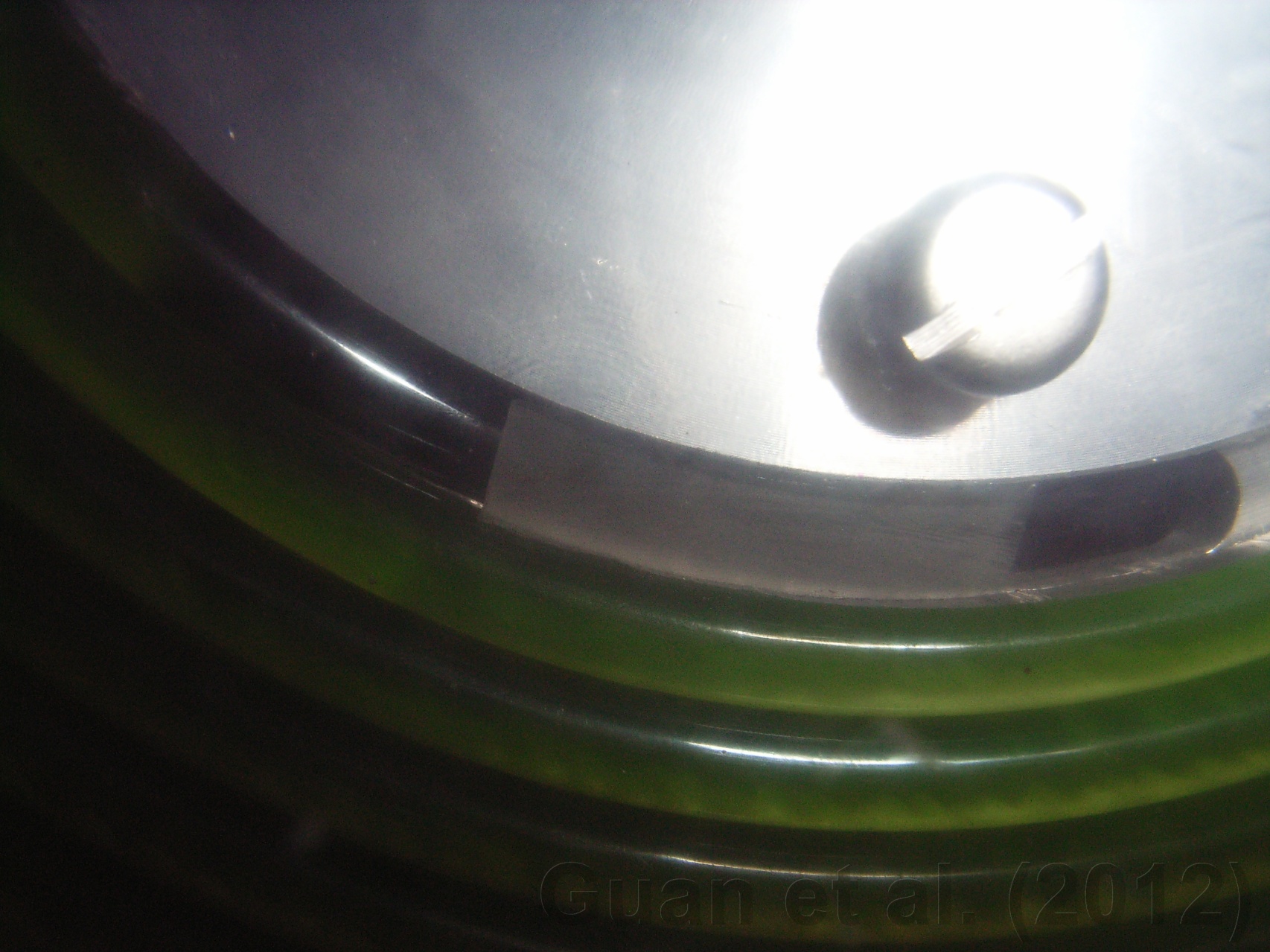 |
| S9-18  A dynamic image for the focused part of the column (shown below)  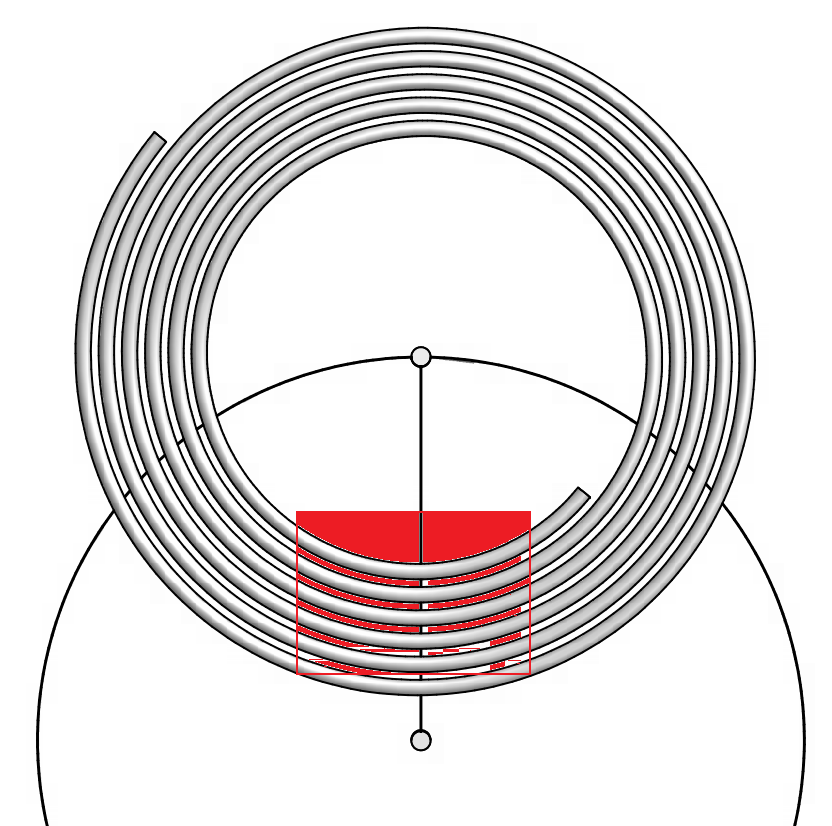 | 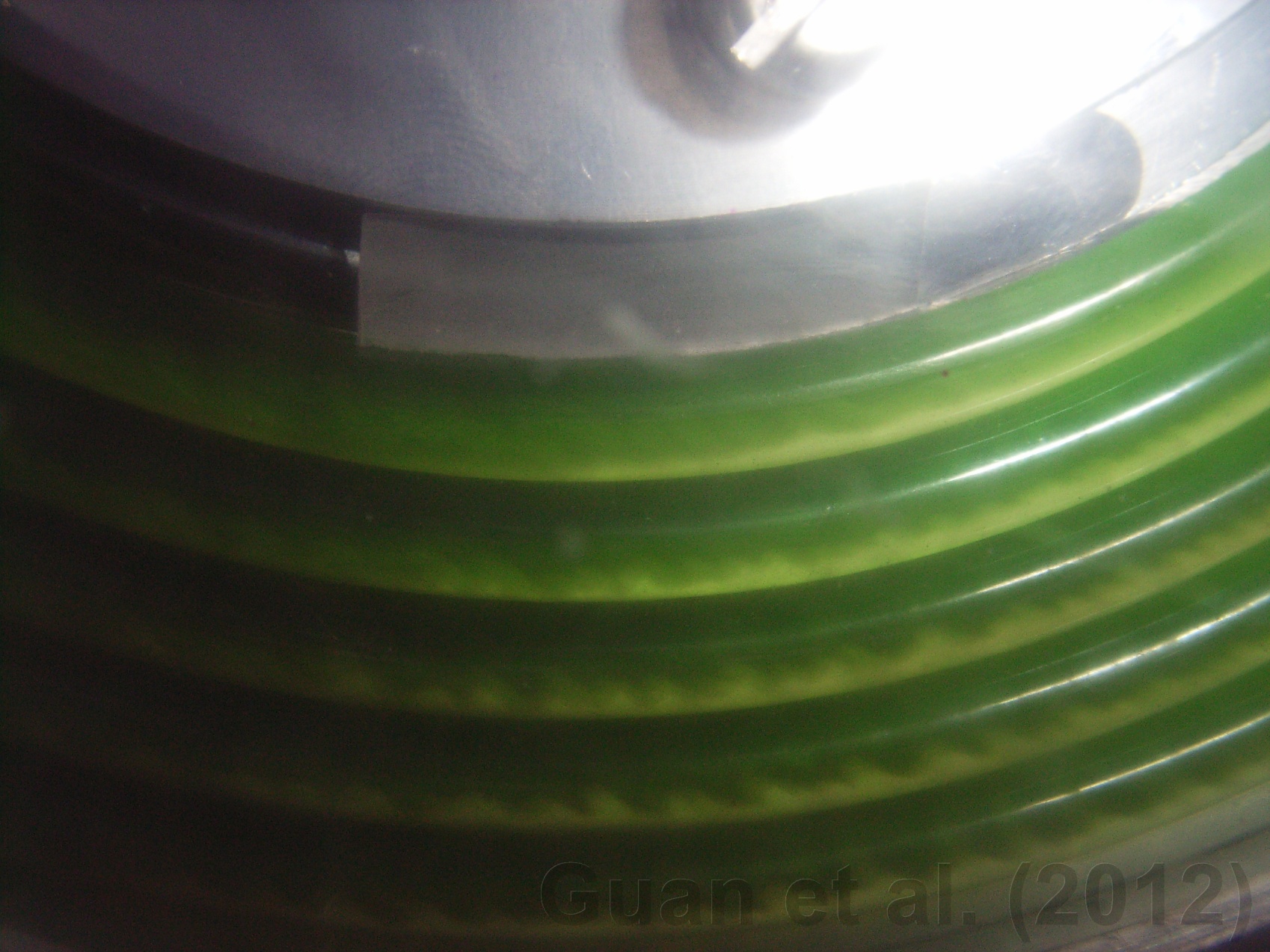 |
| S9-19  A dynamic image for the focused part of the column (shown below)  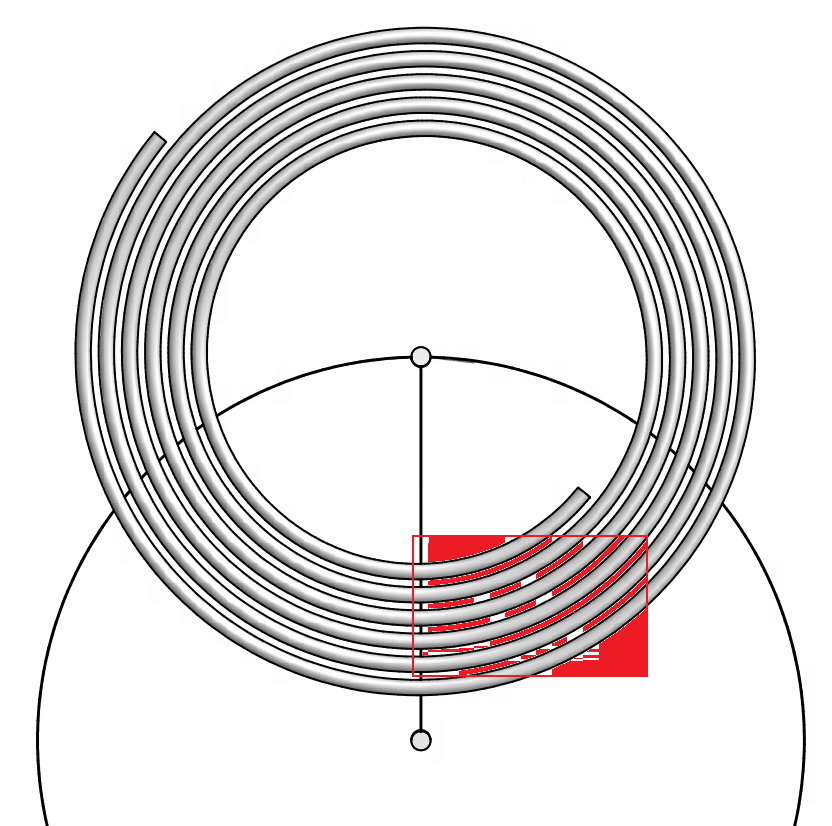 | 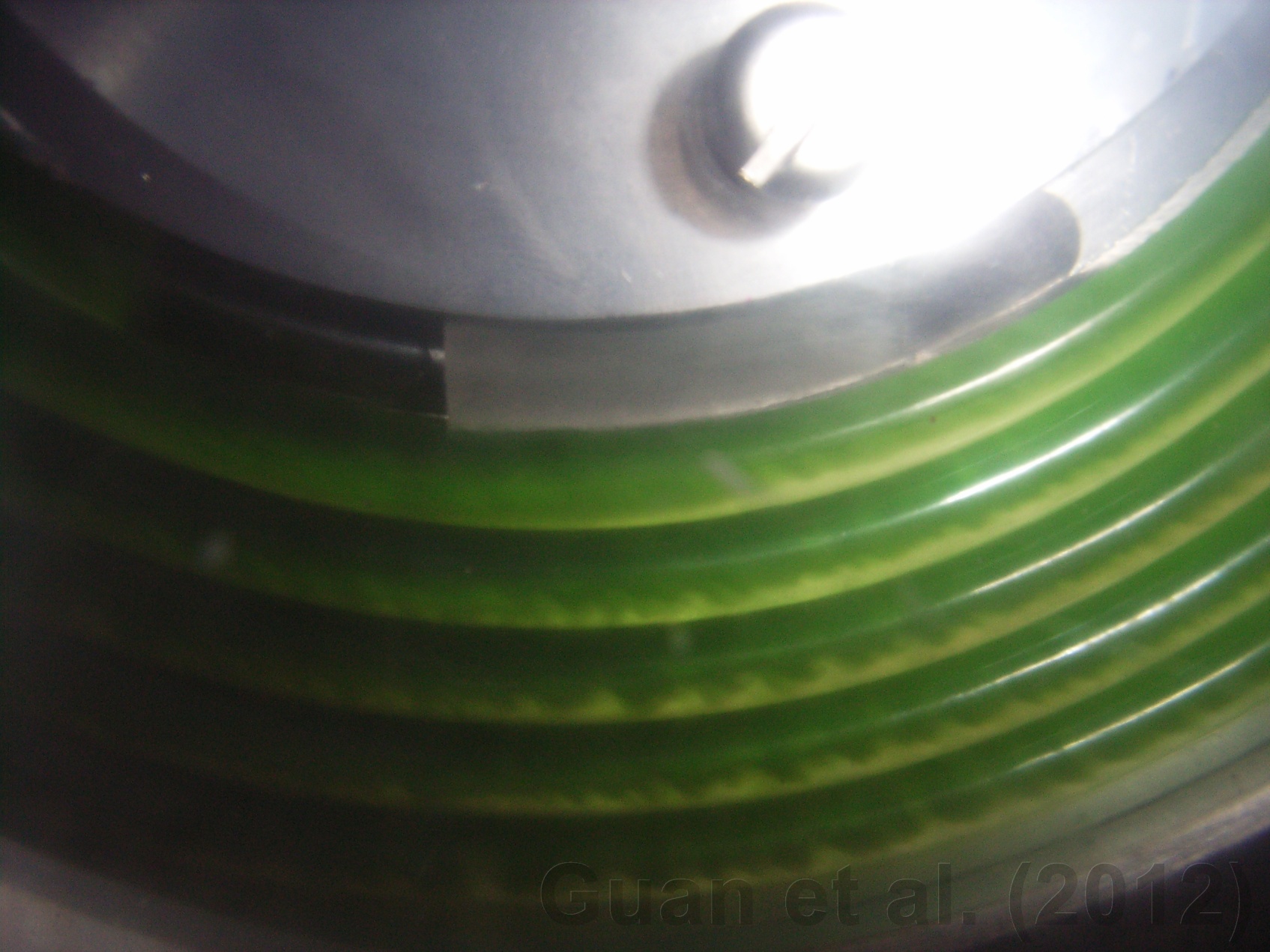 |
| S9-20  A dynamic image for the focused part of the column (shown below)  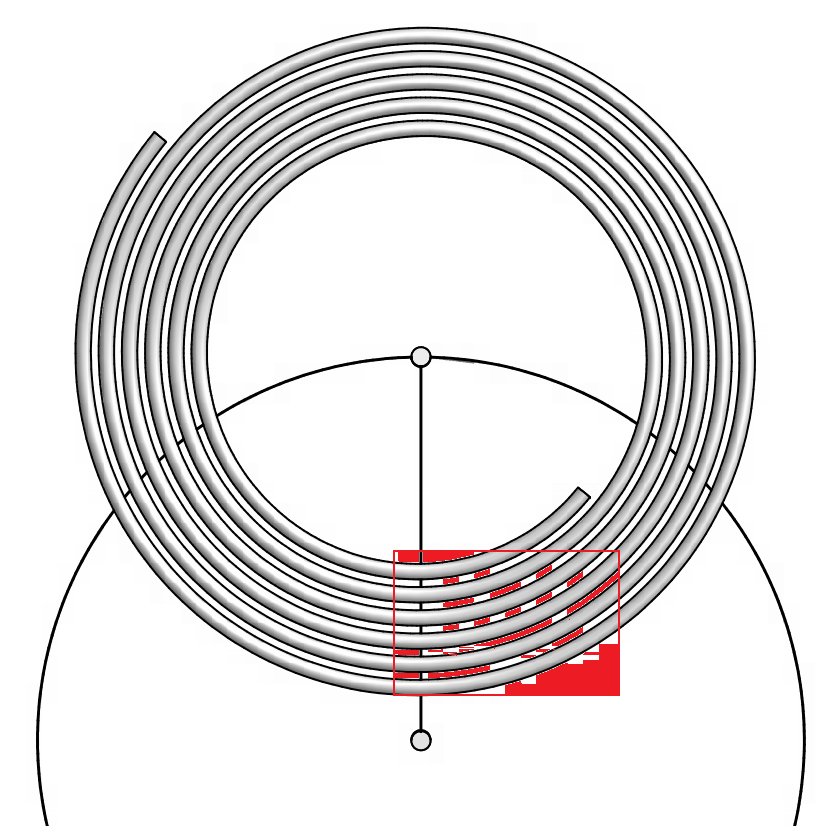 | 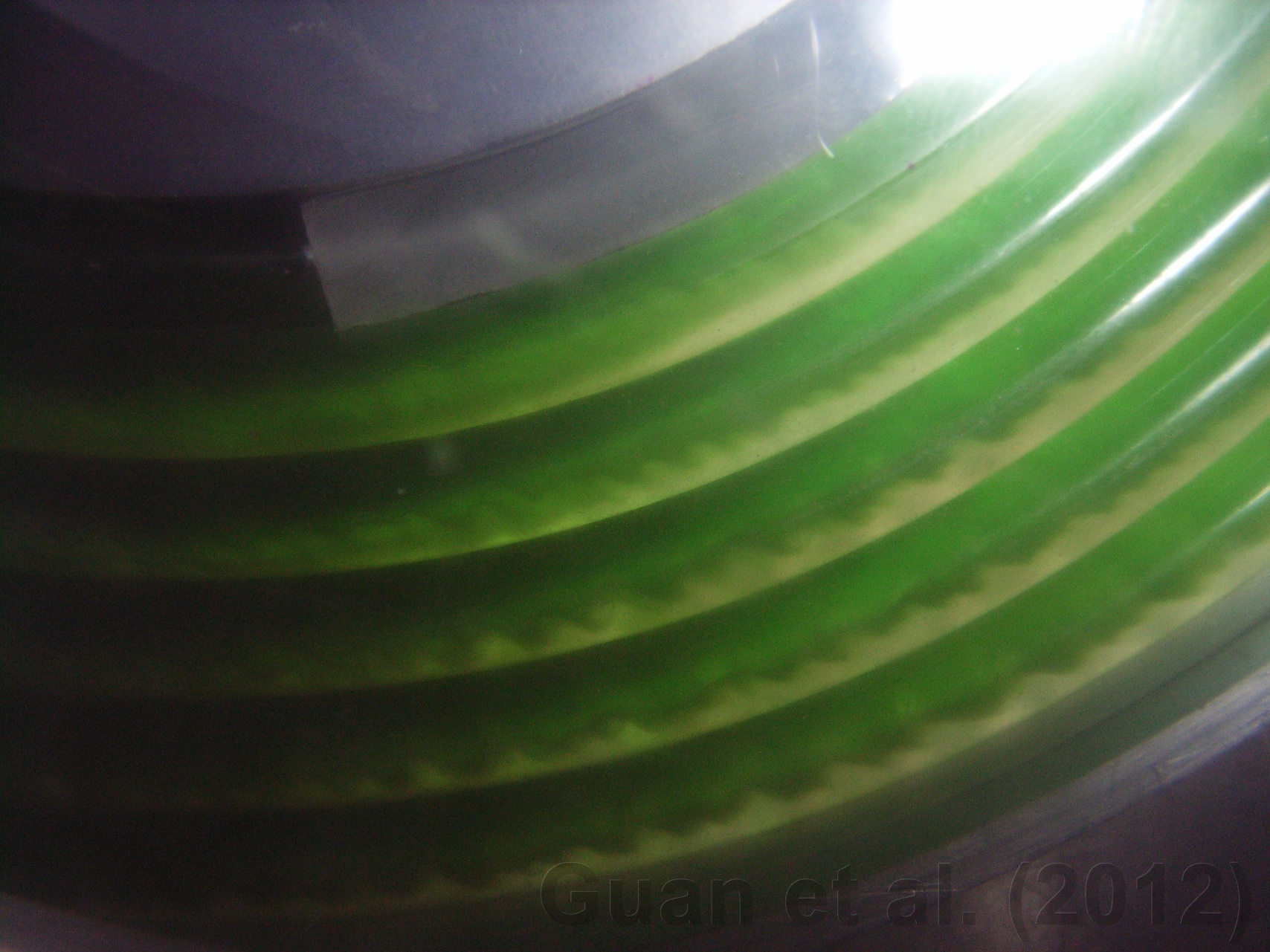 |
| S9-21  A dynamic image for the focused part of the column (shown below)  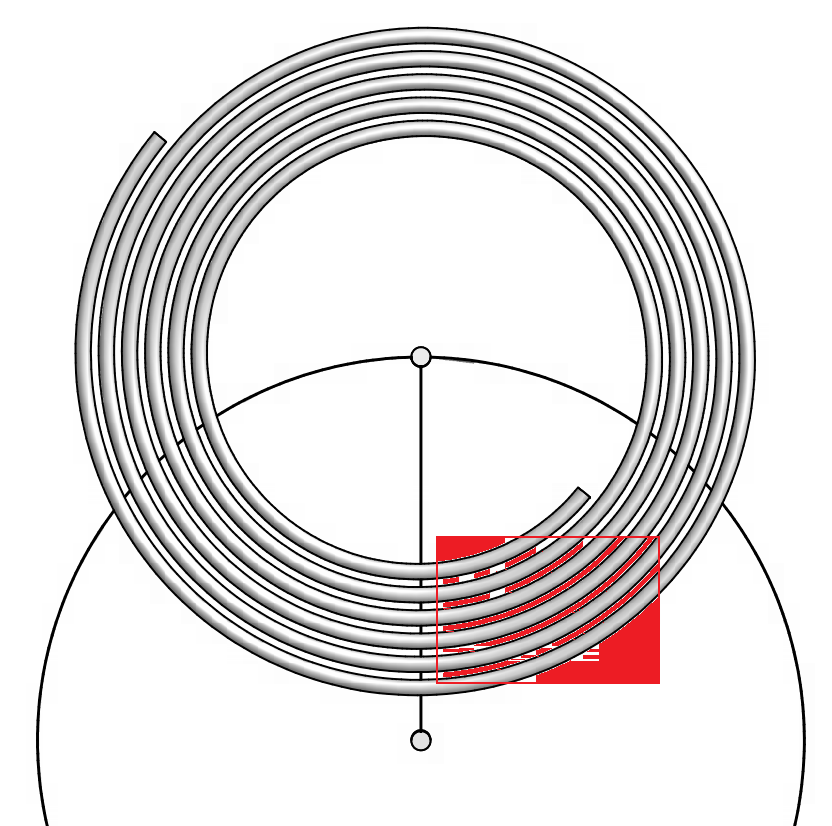 | 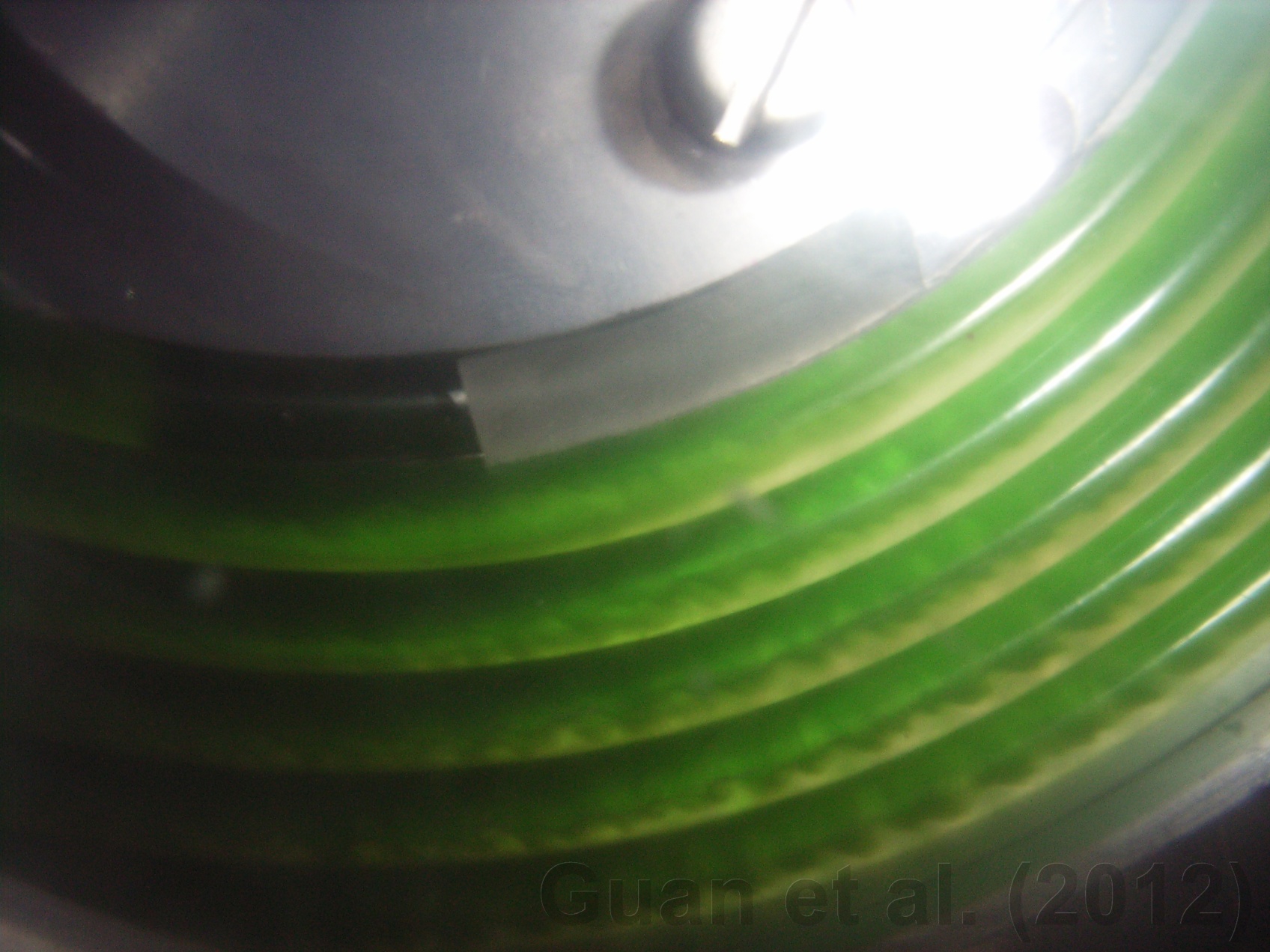 |
| S9-22  A dynamic image for the focused part of the column (shown below)  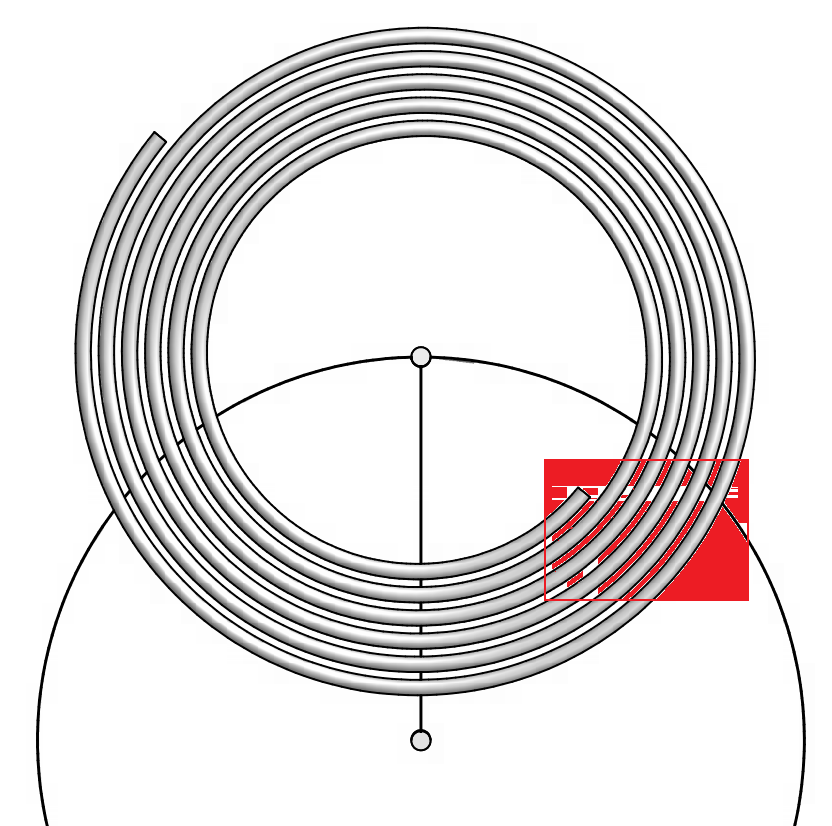 | 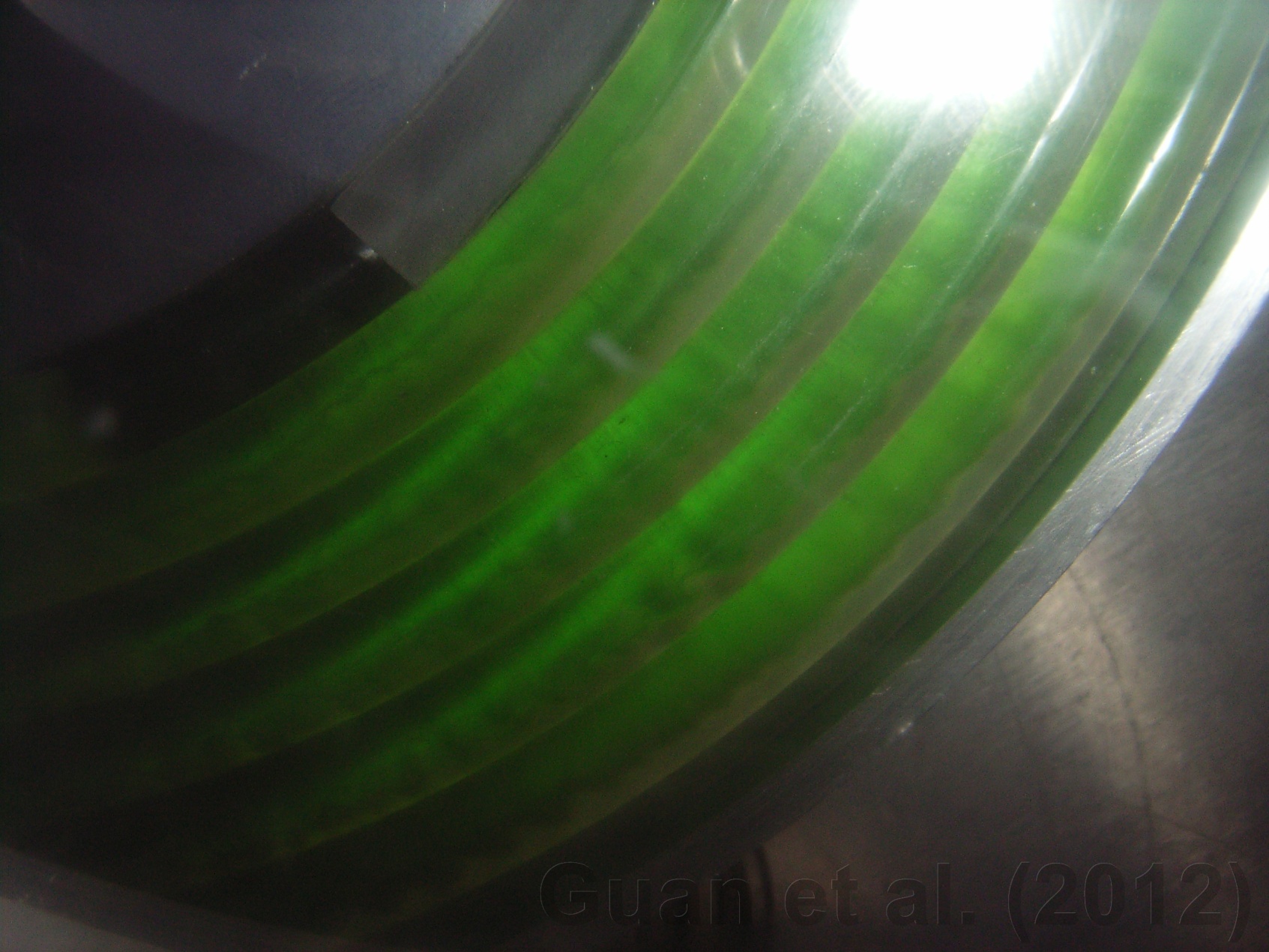 |
| S9-23  A dynamic image for the focused part of the column (shown below)  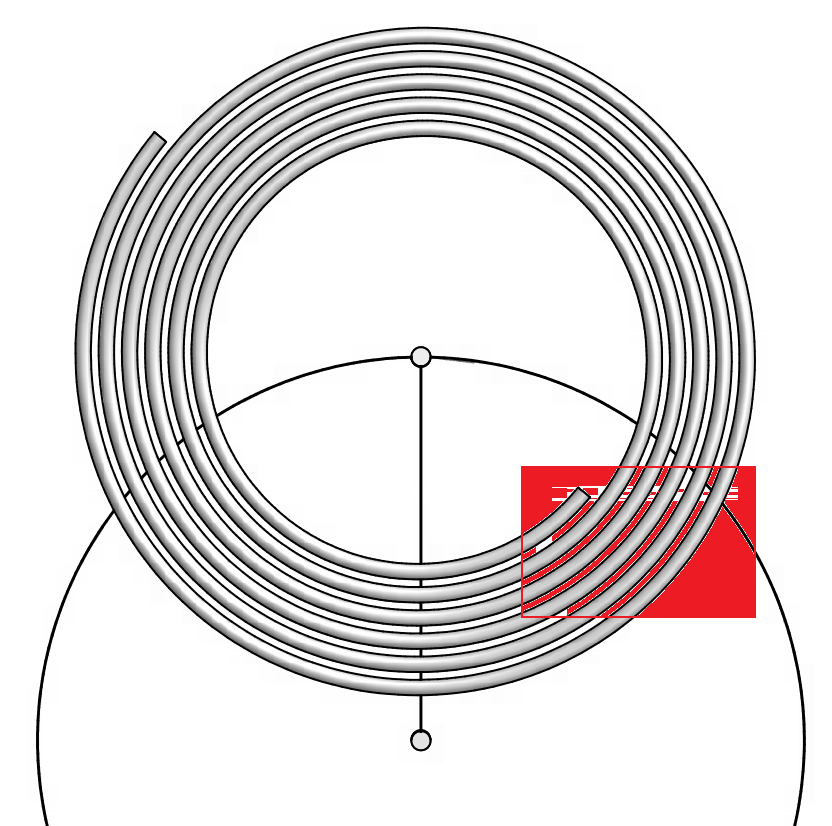 | 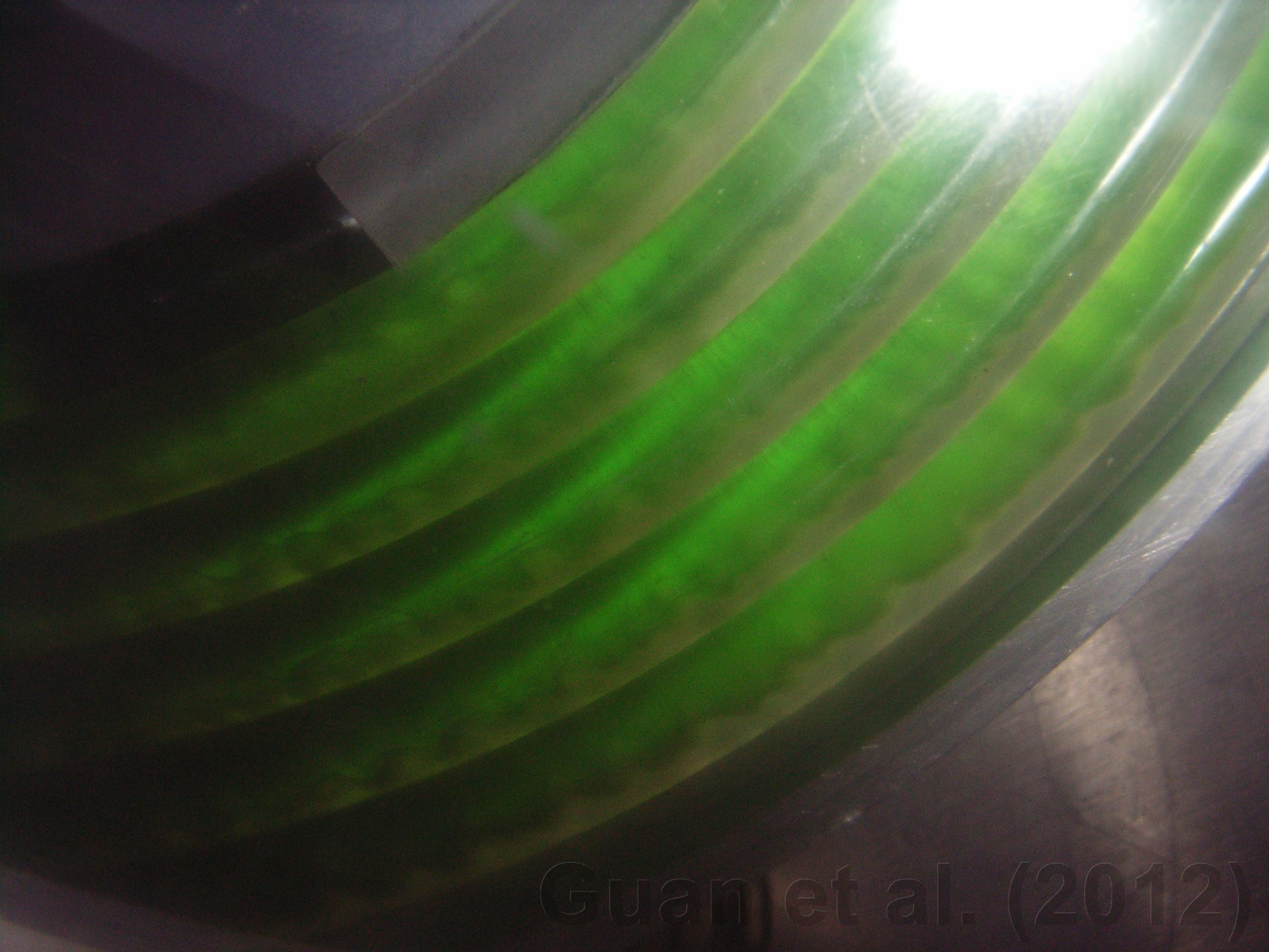 |
| S9-24  A dynamic image for the focused part of the column (shown below)  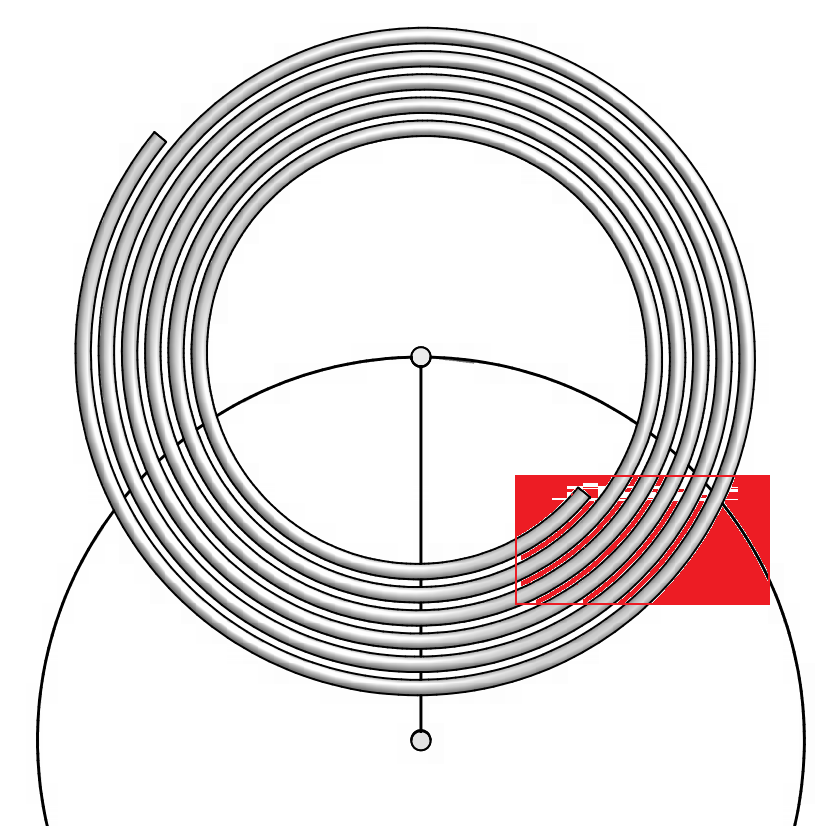 | 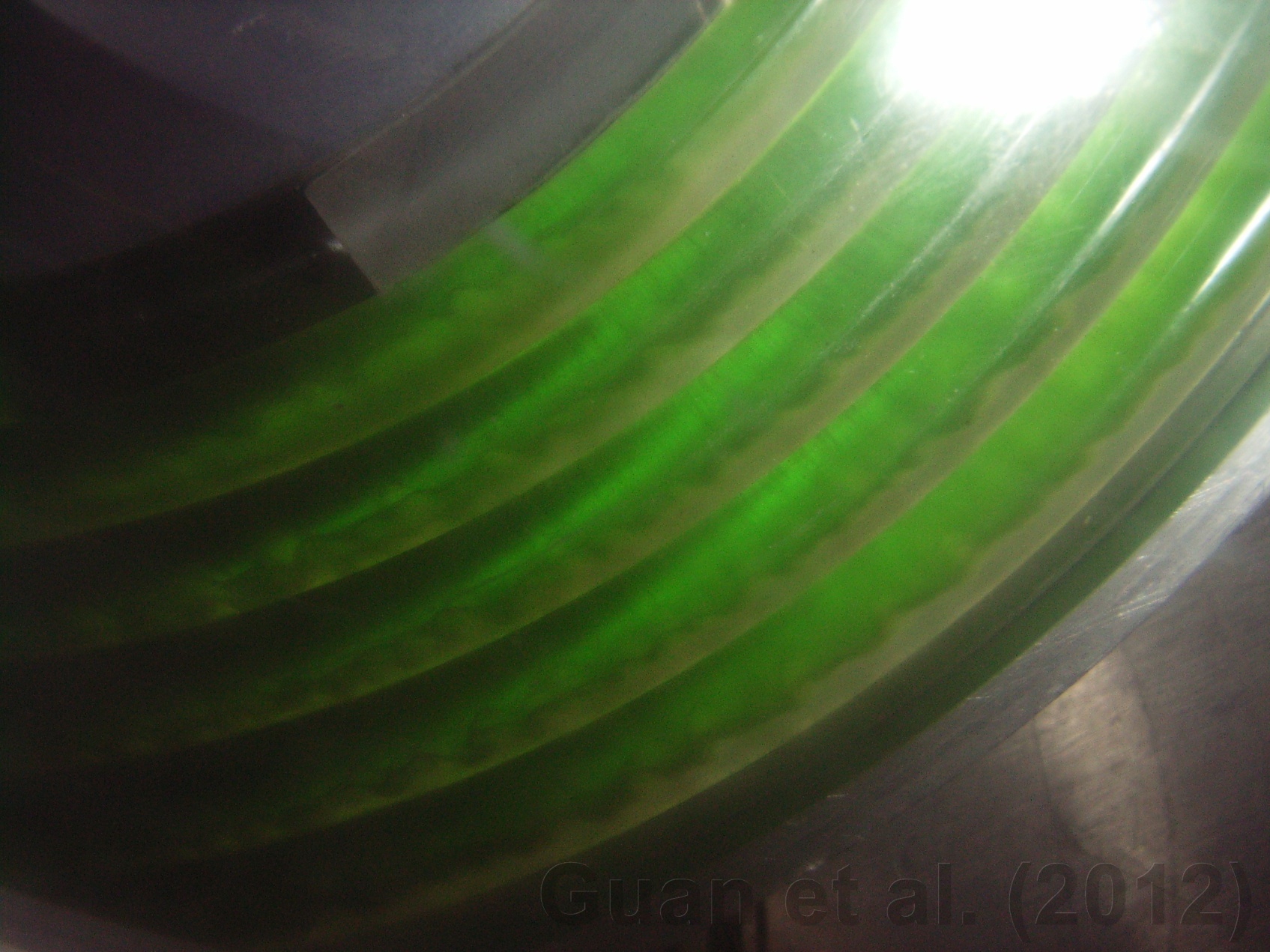 |
| S9-25  A dynamic image for the focused part of the column (shown below)  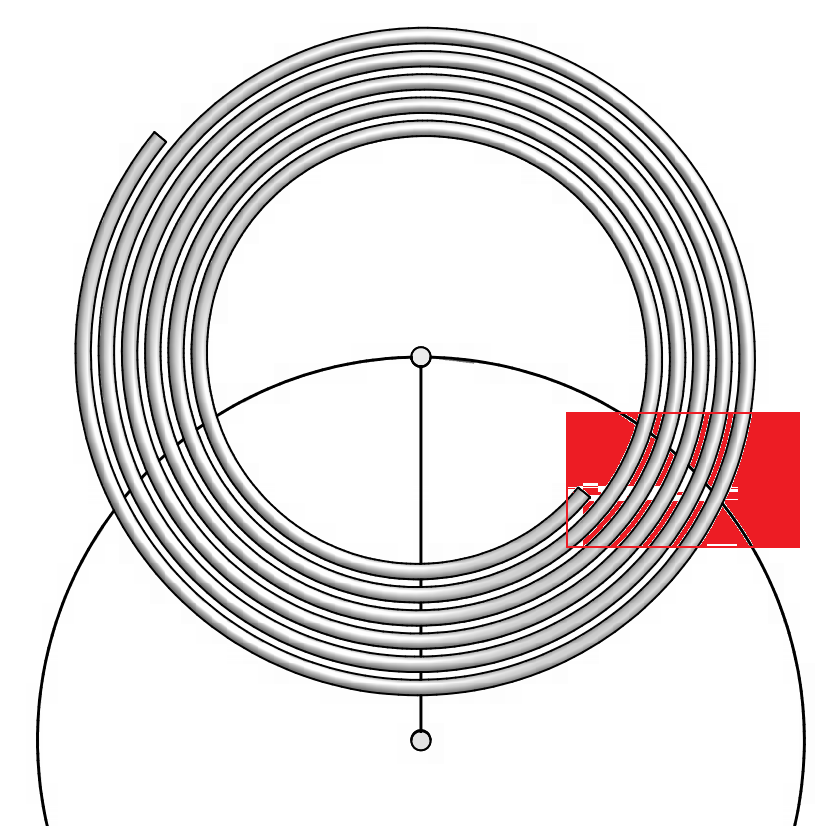 | 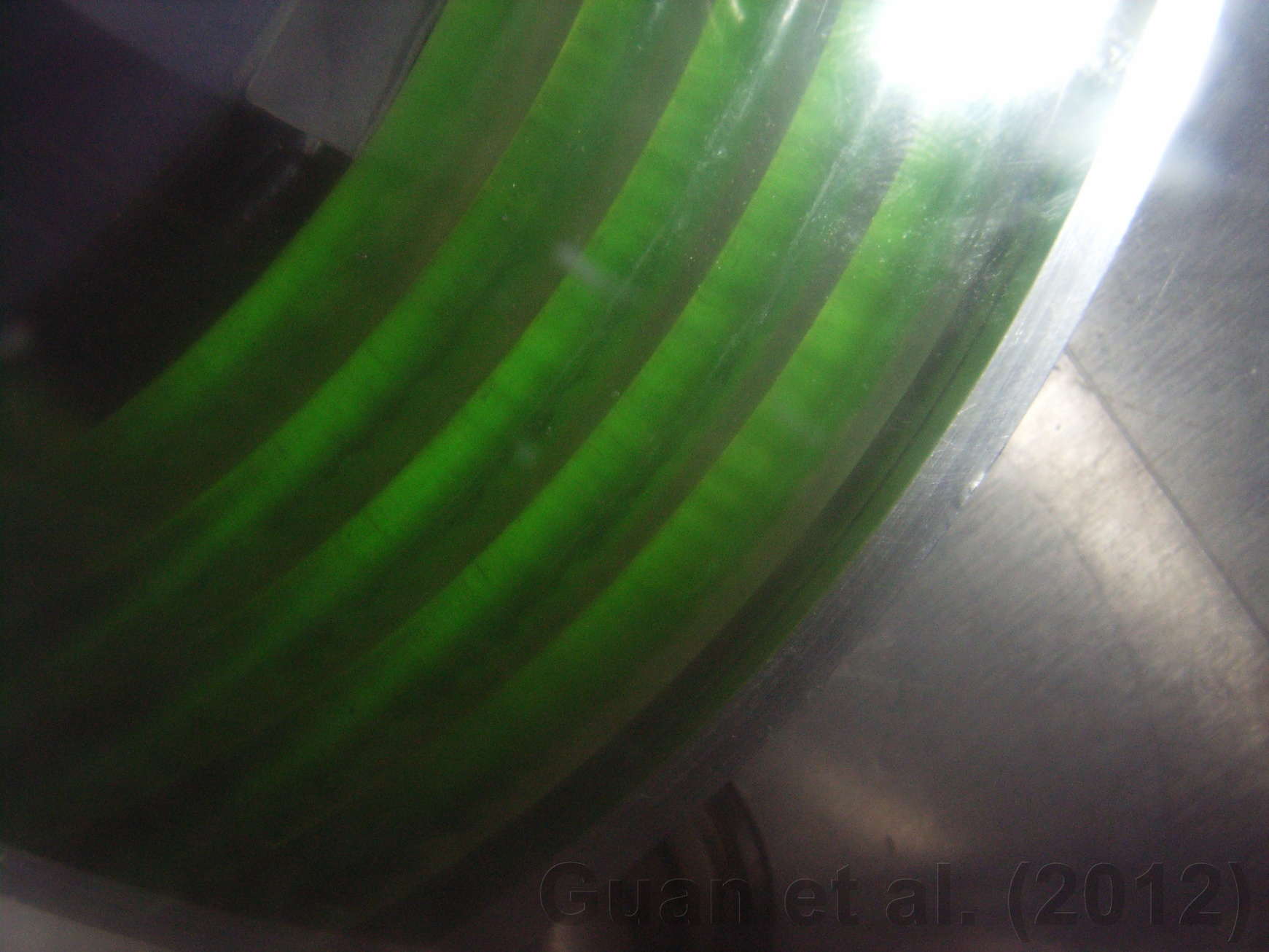 |
| S9-26  A dynamic image for the focused part of the column (shown below)  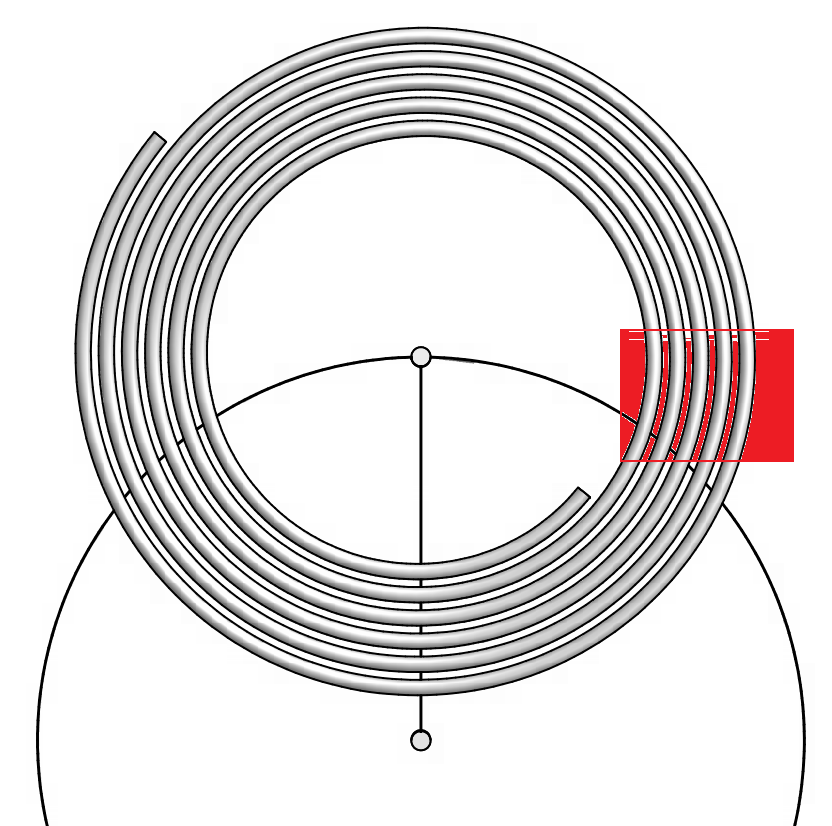 | 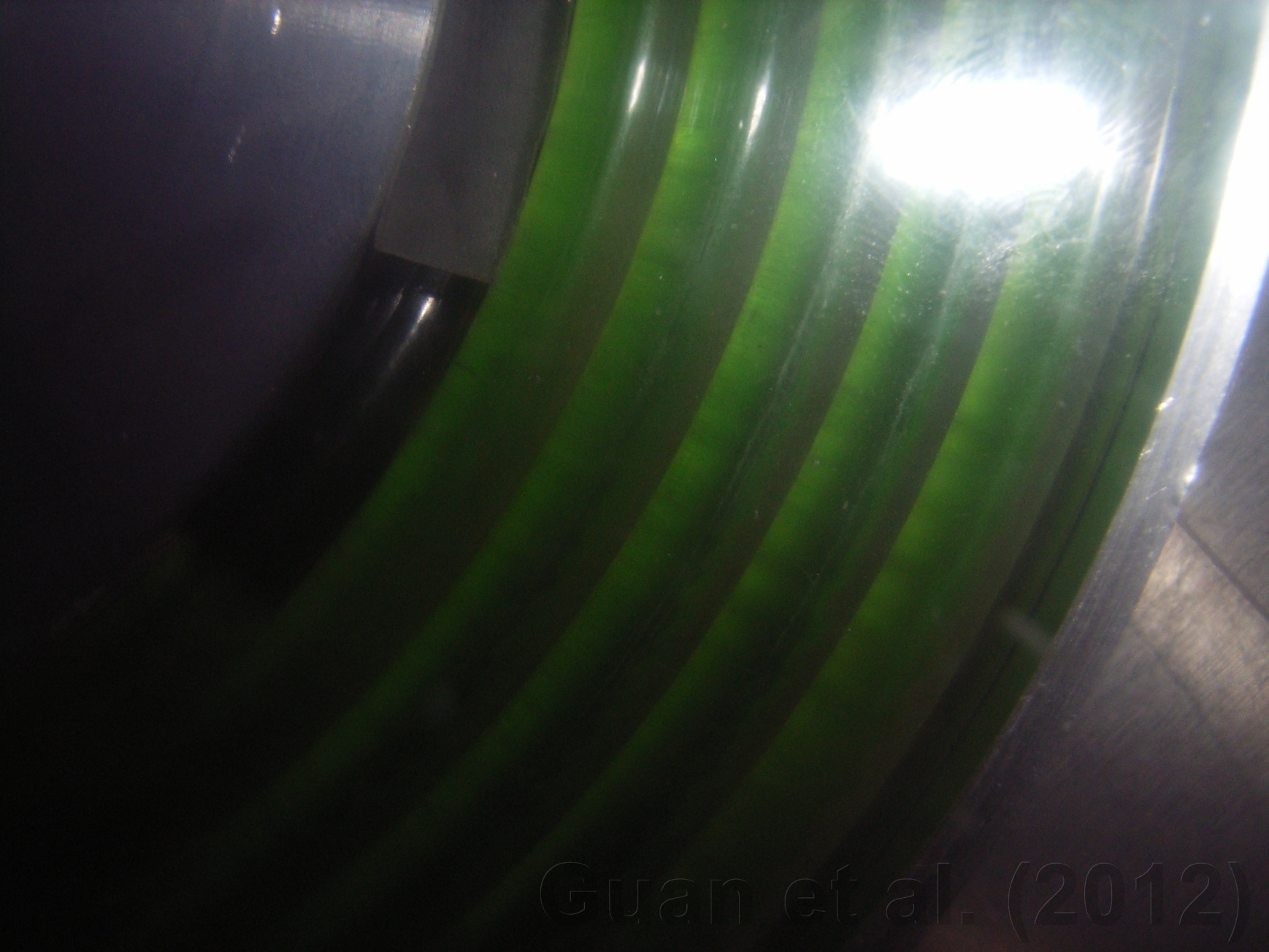 |
| S9-27  A dynamic image for the focused part of the column (shown below)  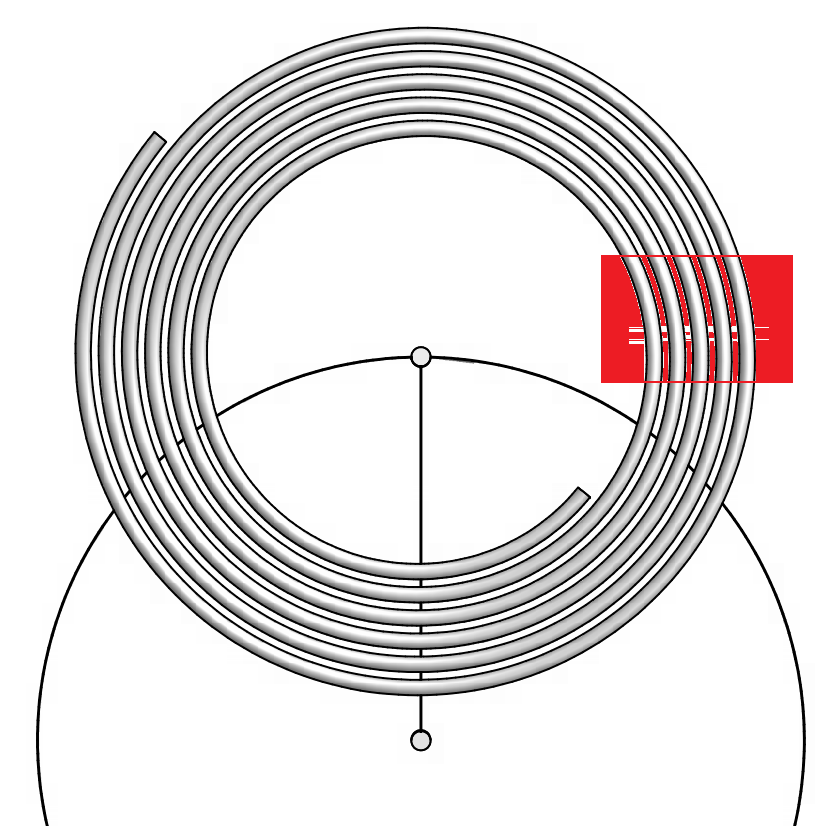 | 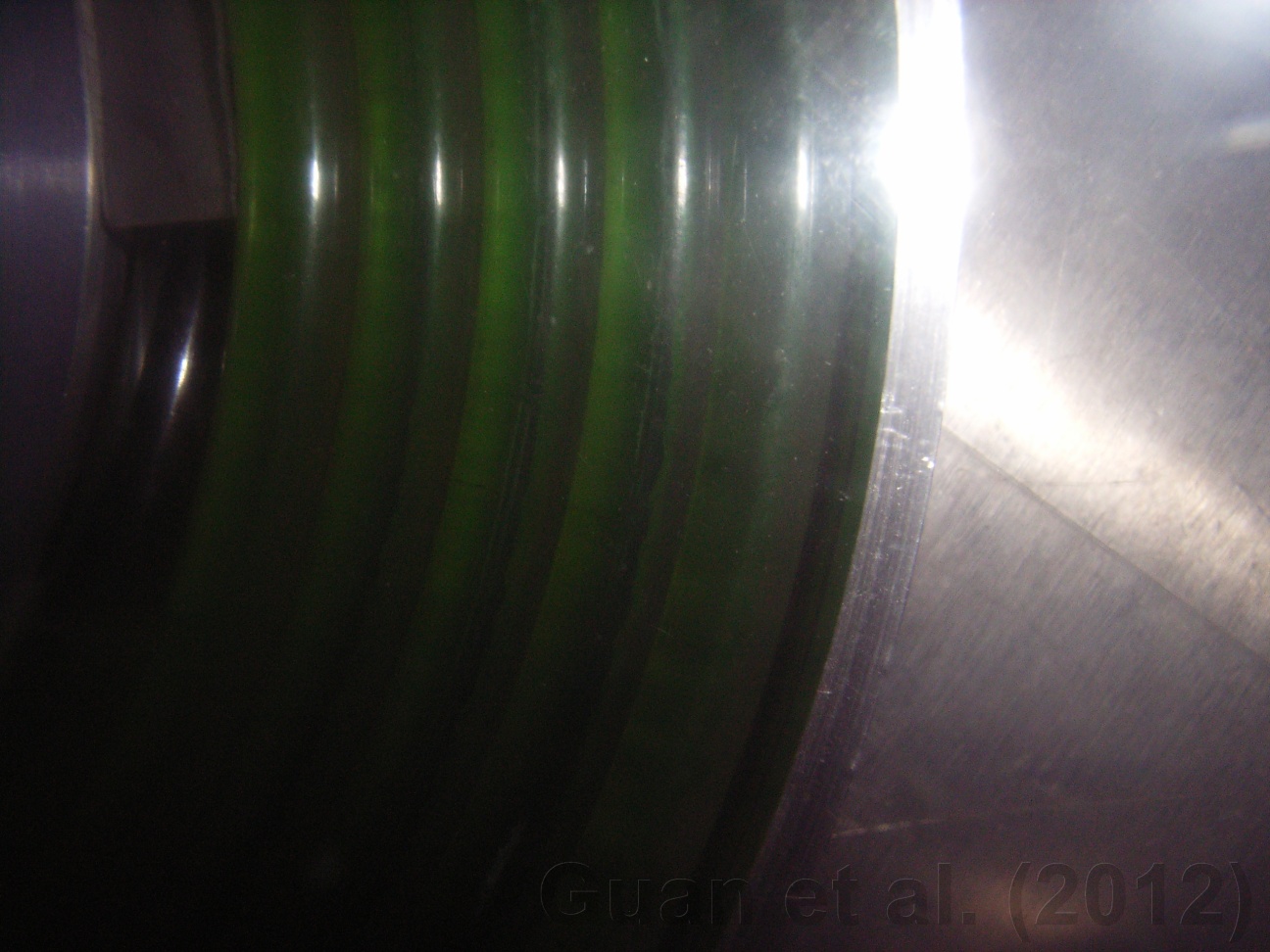 |
| S9-28  A dynamic image for the focused part of the column (shown below)  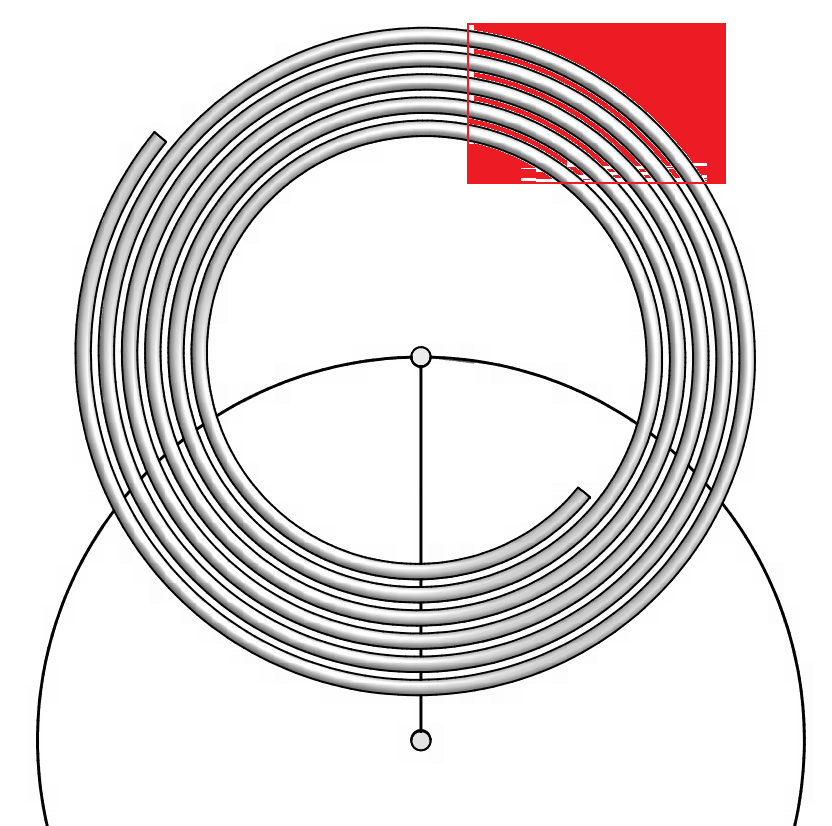 | 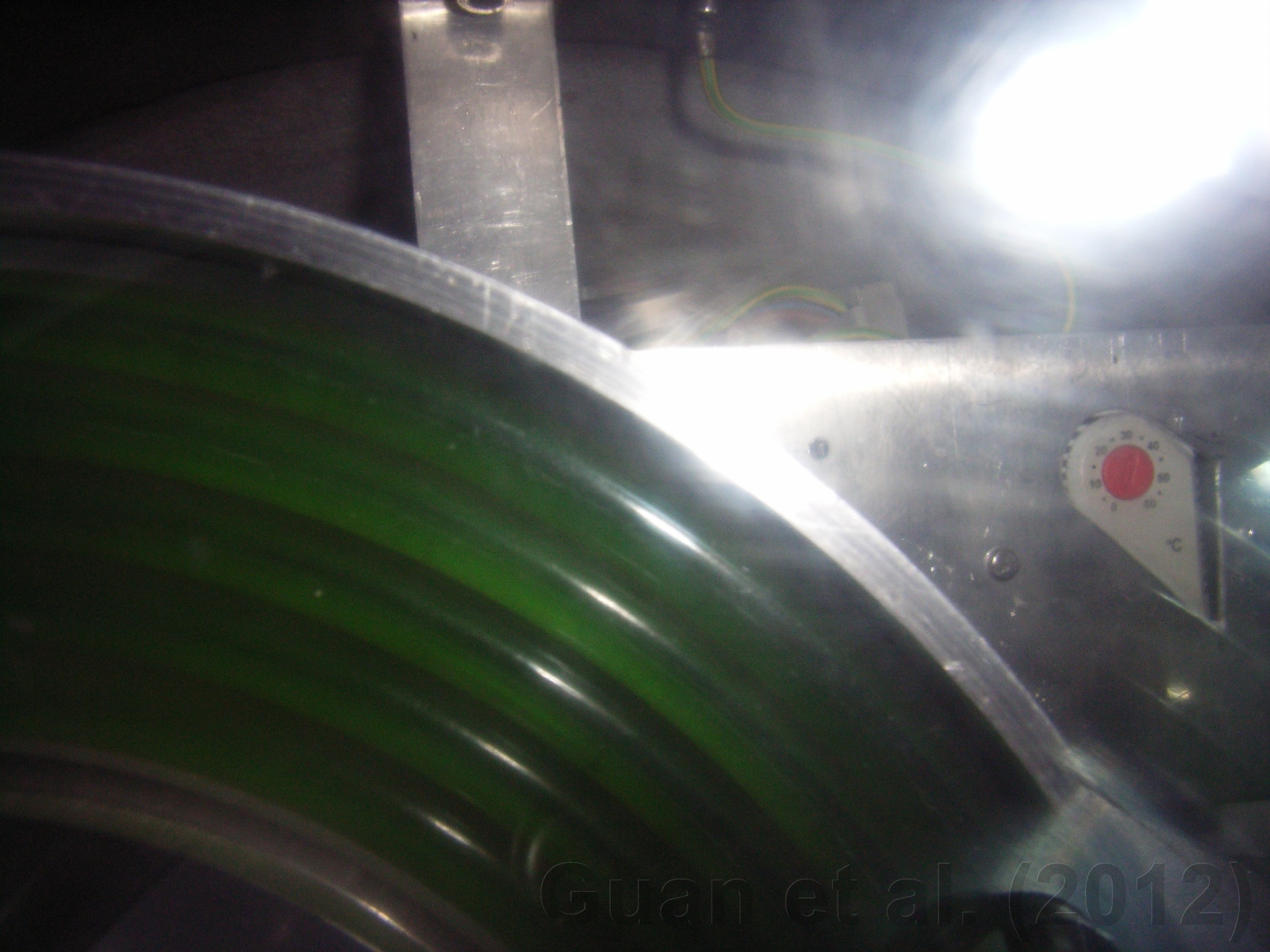 |
| S9-29  A dynamic image for the focused part of the column (shown below)  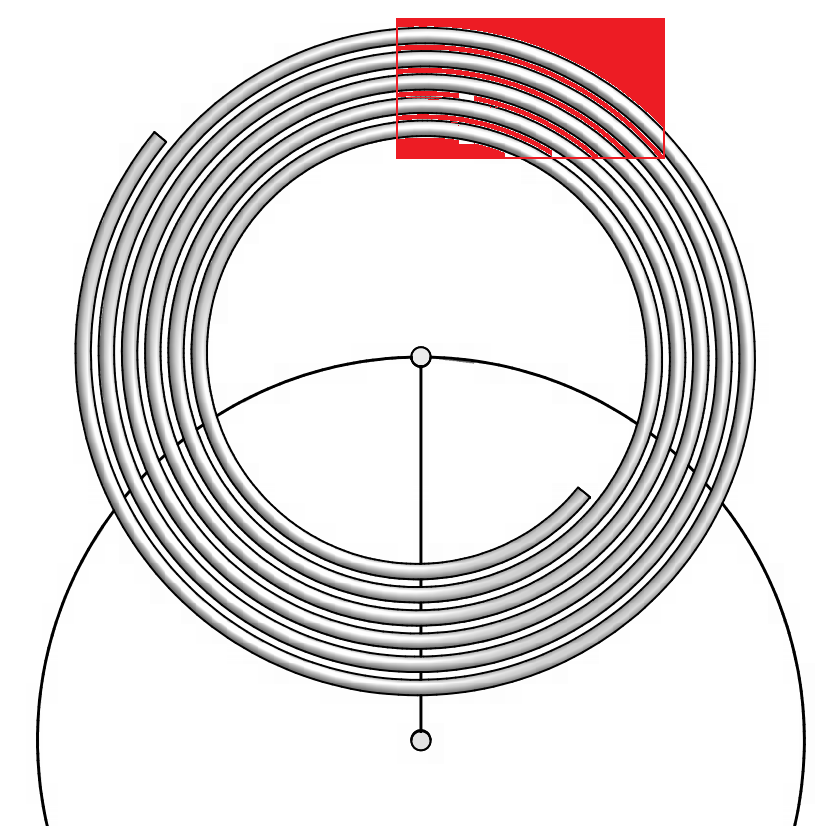 | 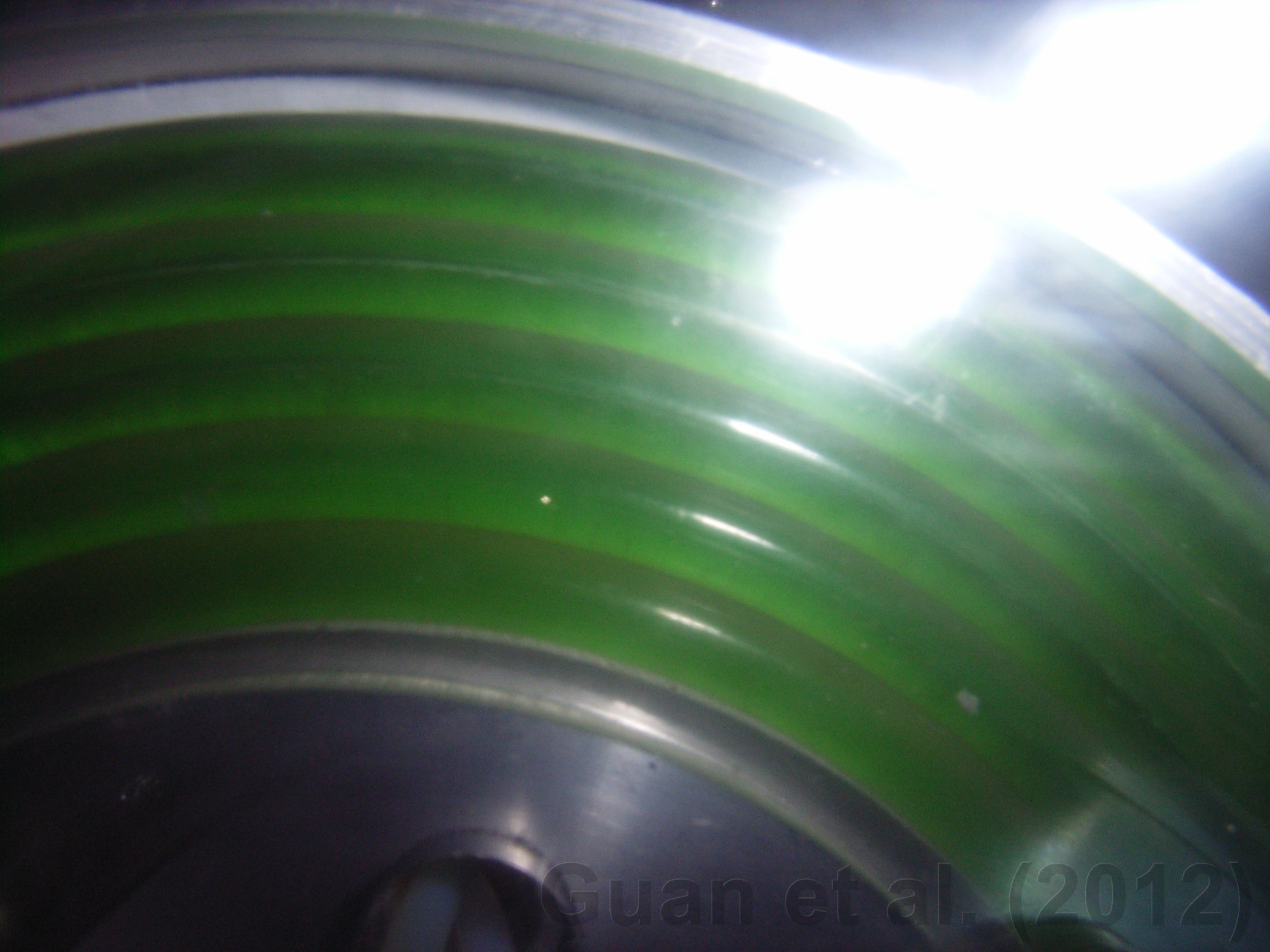 |
| S9-30  A dynamic image for the focused part of the column (shown below)  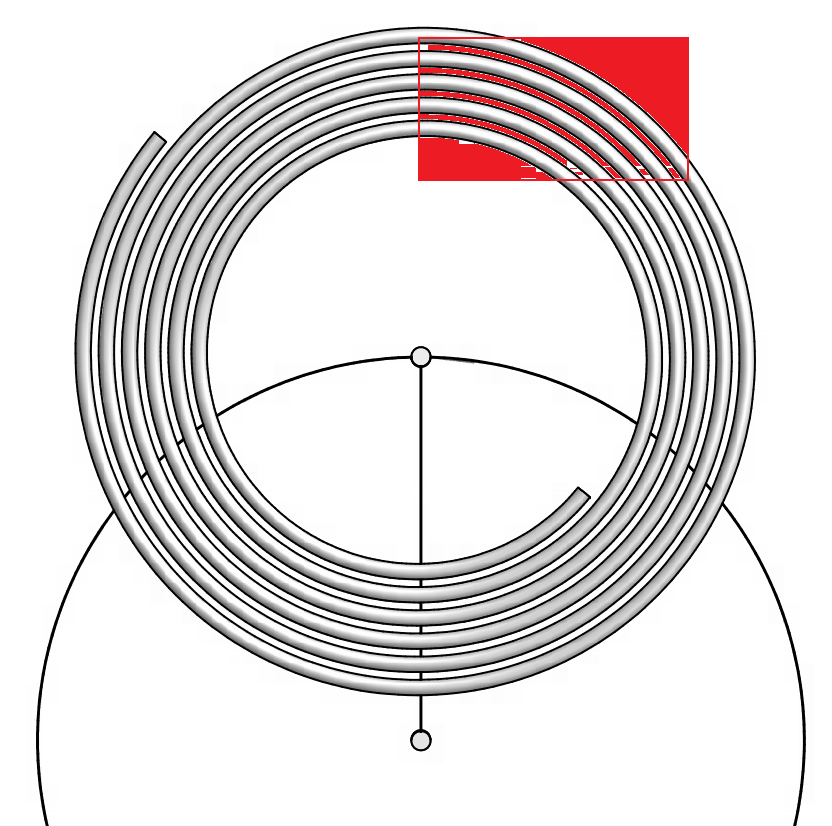 | 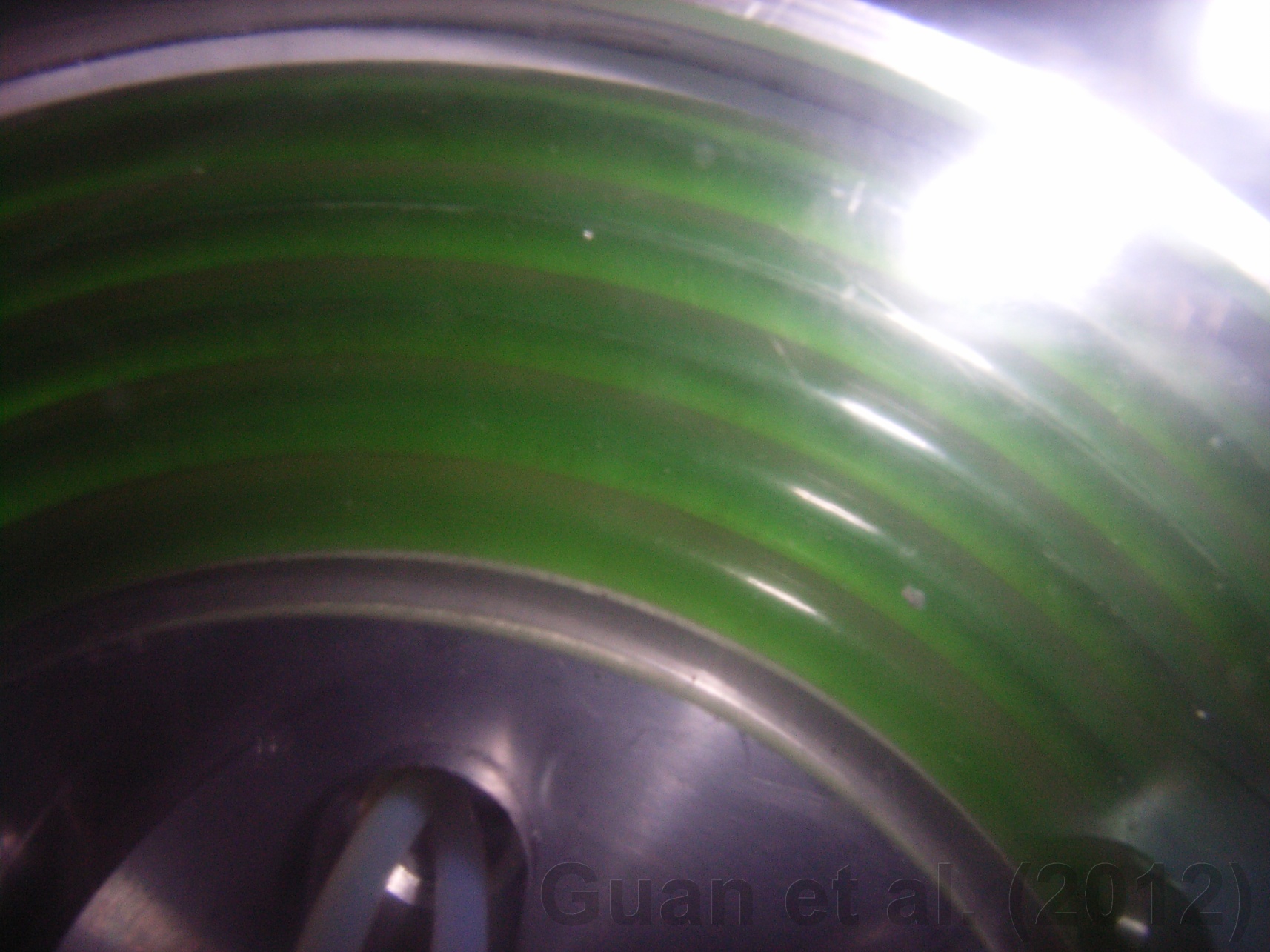 |
| S9-31  A dynamic image for the focused part of the column (shown below)  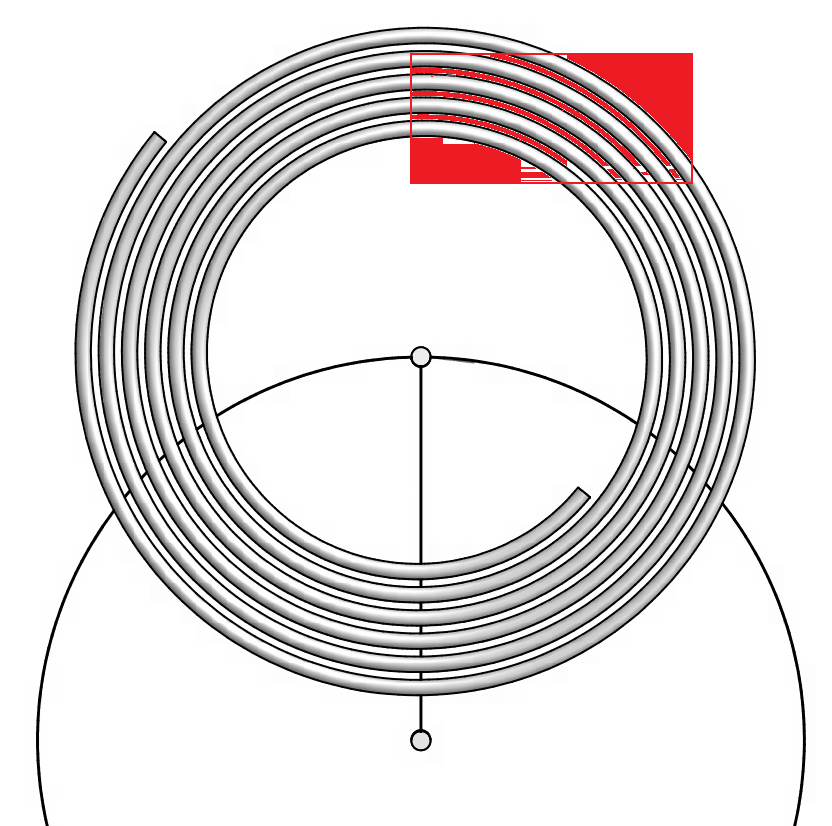 | 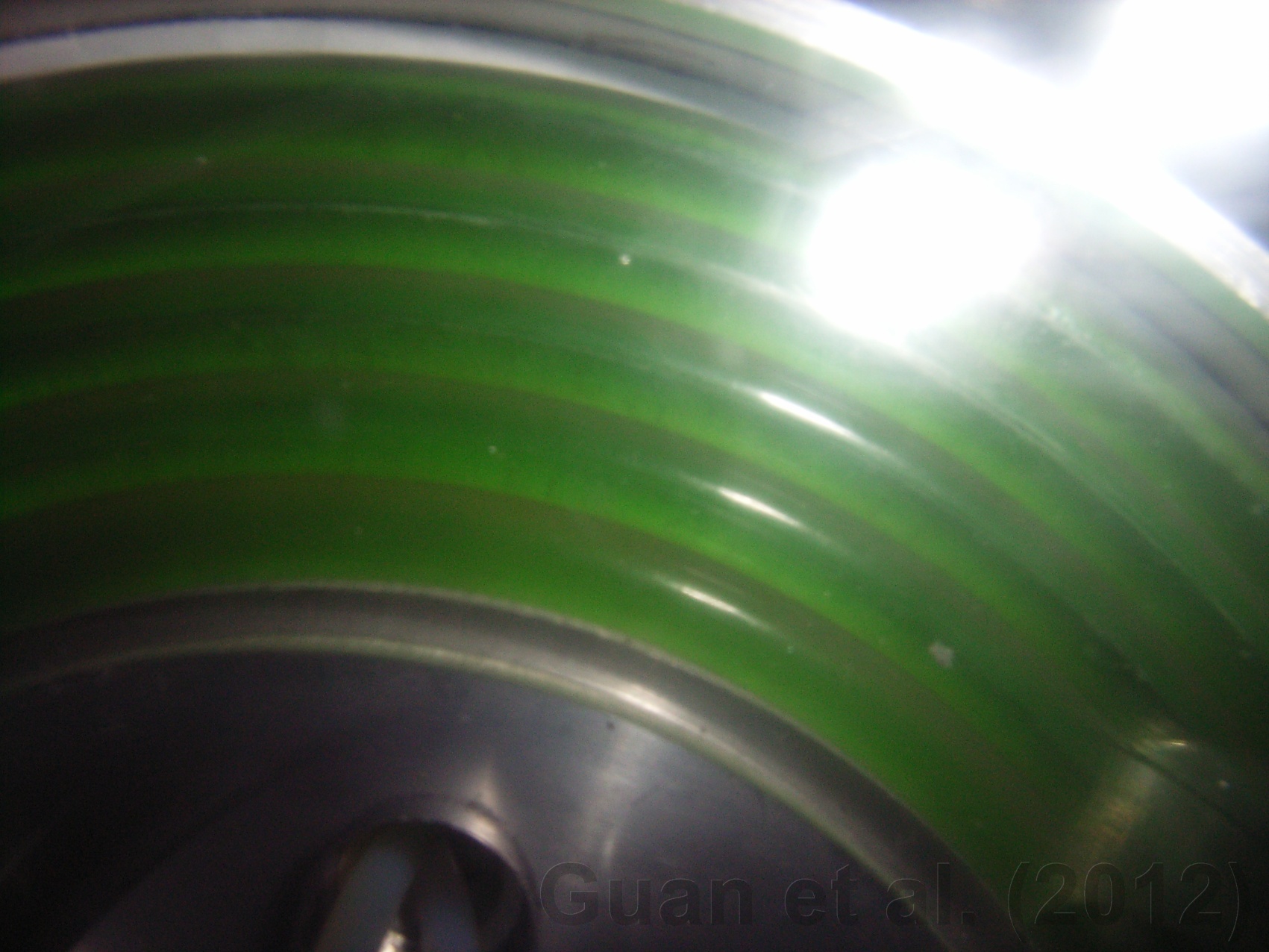 |
| S9-32  A dynamic image for the focused part of the column (shown below)  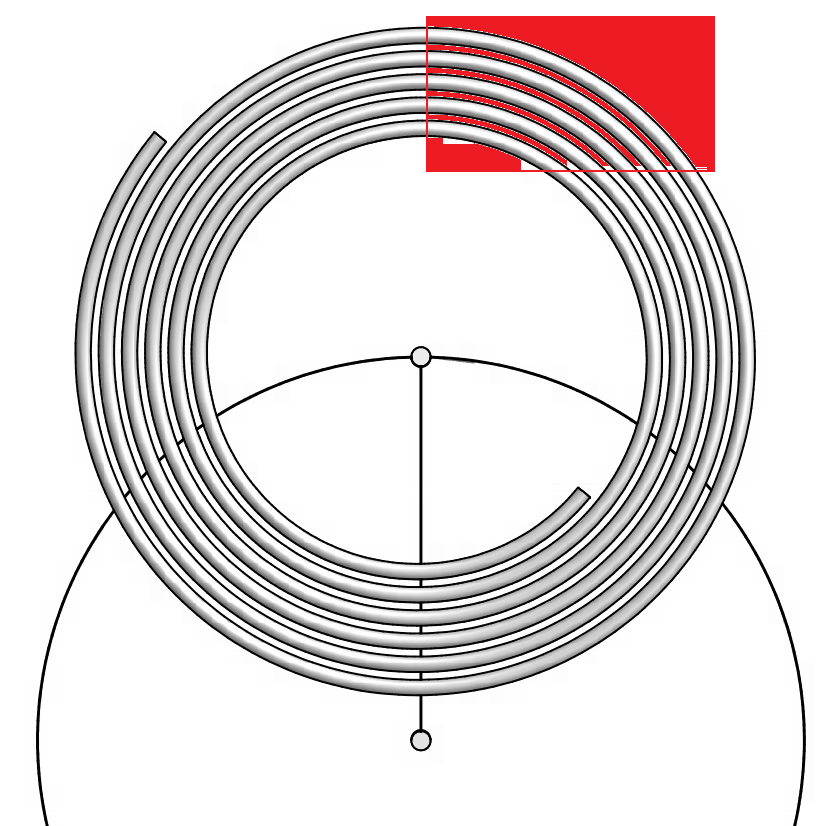 | 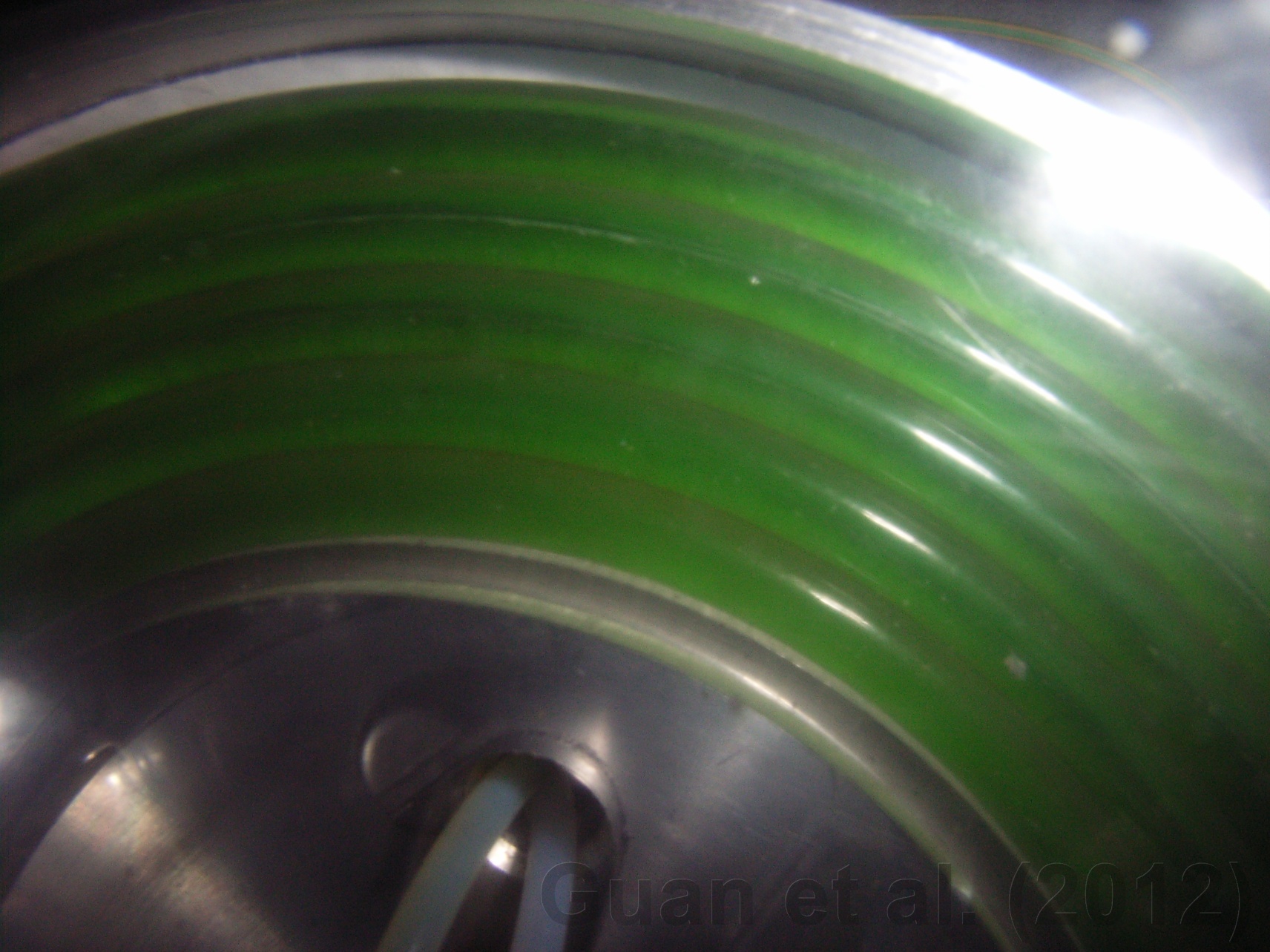 |
| S9-33  A dynamic image for the focused part of the column (shown below)  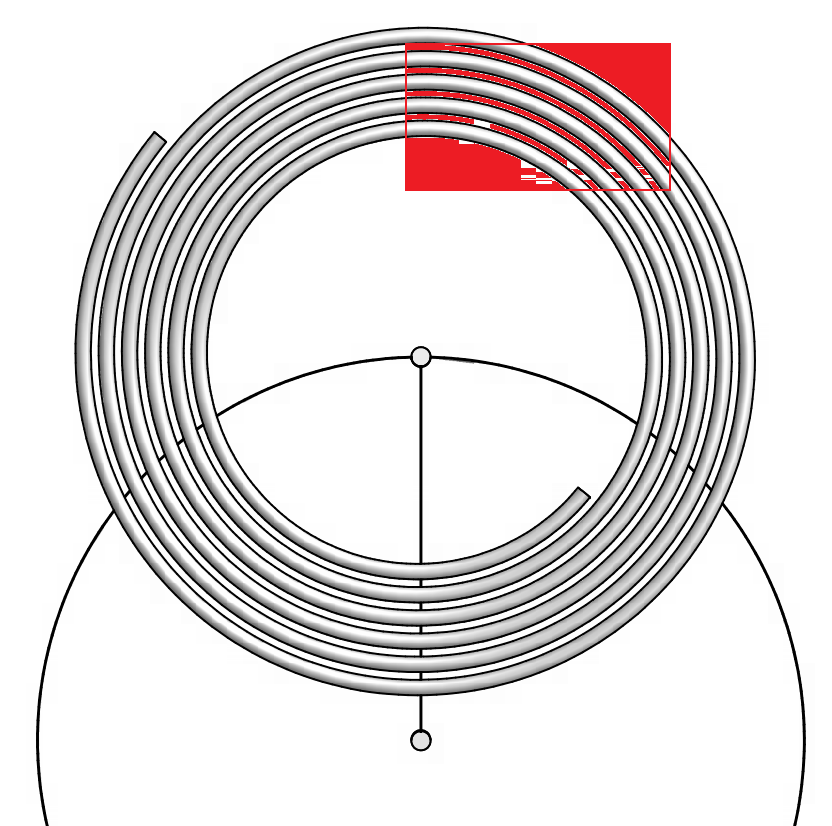 | 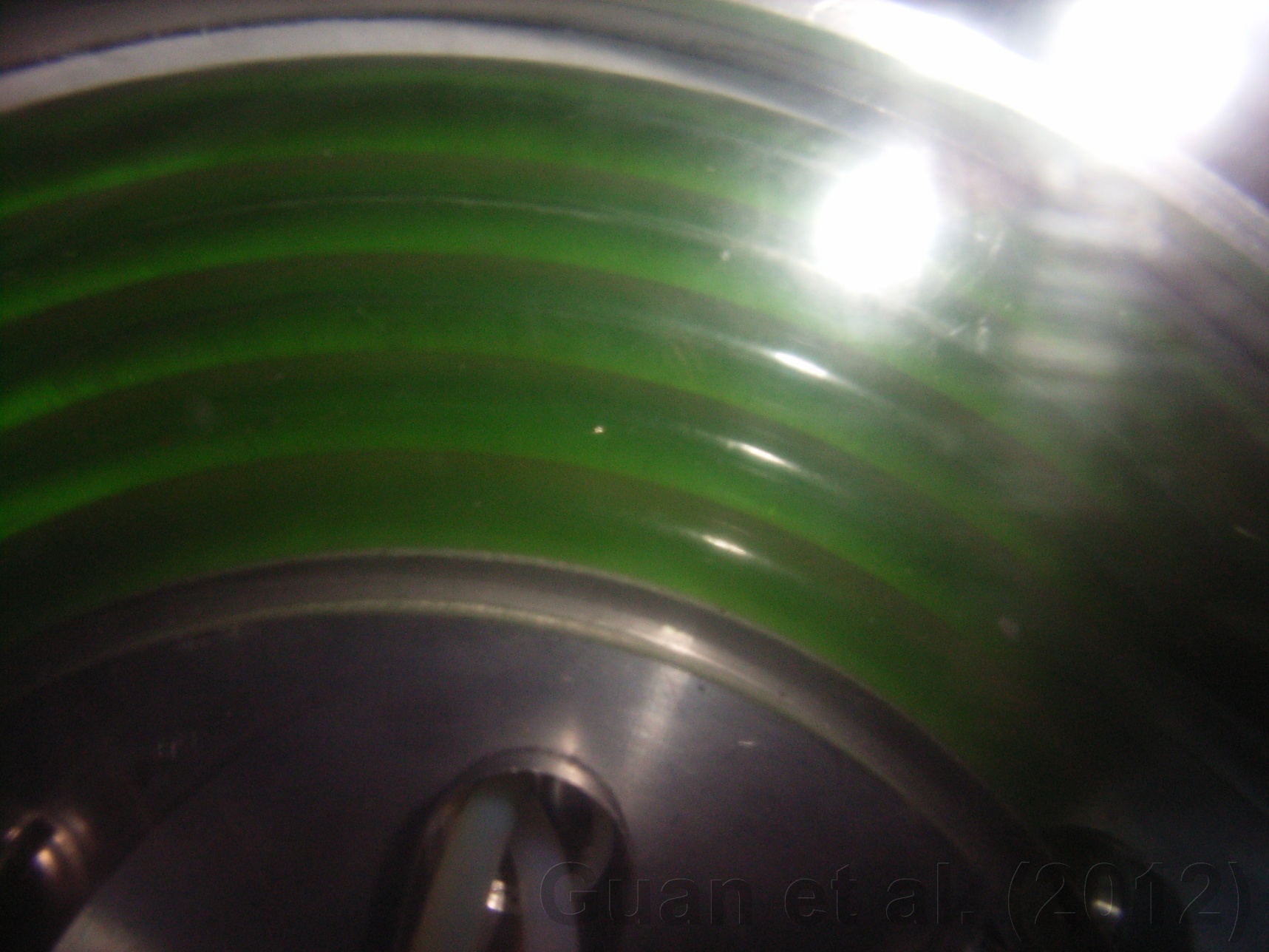 |
